# Mechanistic insights into transcriptional regulation of ARHGAP36 expression identify a factor predictive of neuroblastoma survival

**DOI:** 10.7554/eLife.108827

**Published:** 2026-07-06

**Authors:** Serhiy Havrylov, Armin M Gamper, Ordan J Lehmann

**Affiliations:** 1 Department of Medical Genetics, University of Alberta Edmonton Canada; 2 Department of Ophthalmology, University of Alberta Edmonton Canada; 3 Department of Oncology, Cross Cancer Institute, University of Alberta Edmonton Canada; University of California, San Francisco United States; University of Oxford United Kingdom

**Keywords:** forkhead transcription factor, Hedgehog signaling, protein kinase A, Arhgap36, neoplasia, Human, Mouse, Viruses

## Abstract

Cancer repeatedly exploits attributes fundamental for morphogenesis to advance malignancy and metastasis. This is illustrated by lineage-specific transcription factors that regulate neural crest migration, representing frequent drivers of malignancy. One such example is the *forkhead* transcription factor FOXC1, where gain of function is a feature of diverse cancers that is associated with an unfavorable prognosis. Using RNA-, ChIP-sequencing and CRISPR interference, we show that Foxc1 binds a locus in a region of closed chromatin to induce expression of Arhgap36, a tissue-specific inhibitor of protein kinase A. Because PKA is a core Hedgehog (Hh) pathway inhibitor, Foxc1’s induction of Arhgap36 expression increases Hh activity. The function of Sufu, a PKA substrate, and a second essential Hh pathway inhibitor, is likewise impaired. The resulting increased Hh pathway output is resistant to pharmacological inhibition of *Smoothened*, a phenotype of more aggressive cancers. The Foxc1–Arhgap36 relationship identified in murine cells was further evaluated in neuroblastoma, a neural crest-derived pediatric malignancy. This demonstrated in a cohort of 1348 patients that high levels of ARHGAP36 are predictive of improved 5-year survival. Accordingly, this study has identified as a novel transcription factor which enhances ARHGAP36 expression, one that induces Hh activity in multiple tissues during development. It also establishes a model by which increased levels of FOXC1 via ARHGAP36 and PKA inhibition dysregulate multiple facets of Hh signaling and provides evidence demonstrating relevance to a common neural-crest-derived malignancy.

## Introduction

The neural crest comprises a multi-potent stem cell population that contributes to the formation of diverse tissues. Through an epithelial-to-mesenchymal transition (EMT) that permits delamination from the neural plate, the acquisition of mesenchymal characteristics enables neural crest cells to transition toward a migratory phenotype. During a frequently lengthy path, neural crest cells proliferate, generating precursor cells that populate targets in multiple tissues and so contribute to the formation of diverse organs. Such attributes, essential to embryonic morphogenesis, are reminiscent of the invasive steps efficiently co-opted by cancer cells to drive malignancy and metastasis.

The lineage-specific transcription factors that regulate neural crest development represent frequent drivers of malignancy. An example of this paradigm is the *forkhead* box transcription factor *FOXC1*, which influences the migration and differentiation of neural crest cells. In addition to key roles in brain, ocular, cardiac, renal, and skeletal development (Kume et al., 1998), FOXC1 is aberrantly expressed in diverse cancers. This overexpression was first identified in basal-like breast cancer (Ray et al., 2010; Ramachandran et al., 2024), the subtype with the worst prognosis, and subsequently in at least fifteen additional tumor types, encompassing both solid tumors and hematological malignancies, such as AML (Somerville et al., 2015; Simeoni et al., 2021). FOXC1 is also a prognostic factor for metastasis and survival, with its expression increasing tumor proliferation, invasion, and dissemination (Han et al., 2017). Roles that include EMT induction, cell cycle control, angiogenesis, and regulation of stem cell populations are thought to promote FOXC1’s adverse oncological effects. However, despite such insights, the precise mechanisms behind FOXC1’s contributions to diverse malignancies remain incompletely defined.

Dysregulation of Hedgehog (Hh) signaling occurs frequently in sporadic and inherited cancers (Raleigh and Reiter, 2019; Zhang and Beachy, 2023; Cong et al., 2025; Johnson et al., 1996) and is estimated to underlie some 30% of malignancies. Hedgehog’s critical role in malignancy is linked to the pathway’s regulation of the Glioma (Gli) transcription factors and the requirement of Hh activity to maintain normal and cancer cell stemness. Notably, *FOXC1* and several of its paralogs are able to induce Hh expression and Hh pathway activity (Chang et al., 1997; Han et al., 2015; Yoshida et al., 2015; Almubarak et al., 2024; Havrylov et al., 2024). The output of the Hh pathway is tightly regulated by three equipotent inhibitors: the Patched transmembrane receptors, Suppressor of Fused (Sufu), and protein kinase A (PKA). Of these, PKA is positioned comparatively late in the signal transduction cascade and, by phosphorylating the GLI oncoproteins, directs their conversion from full-length active to truncated repressor forms (Zhang and Beachy, 2023; Wang et al., 2000; Niewiadomski et al., 2014). As a result, PKA controls the transcriptional outcome of Hh signaling. In recent years, a tissue-specific PKA antagonist has been identified. This Rho GTPase-activating protein, Arhgap36, alters the balance between activator and repressor categories of GLI by inhibiting PKA, thereby inducing strong Hh pathway activation (Rack et al., 2014; Eccles et al., 2016; Zhang et al., 2019).

In this study, RNA- and ChIP-sequencing coupled with CRISPR interference were used to demonstrate that Foxc1 binds a locus located in a region of closed chromatin to induce Arhgap36 expression. In turn, Arhgap36 depletes the levels of PKA and its catalytic subunit PKAC, both strongly activating Hh signaling and making signal transduction less dependent on regulation via *Smoothened*. The oncological relevance of the Foxc1–Arhgap36 relationship is demonstrated in neuroblastoma, a neural crest-derived pediatric malignancy where ARHGAP36 levels predict 5-year mortality. Consequently, our study identifies a mechanism through which overexpression of FOXC1 dysregulates the Hedgehog pathway and contributes to malignancy.

## Results

**Figure 1.**
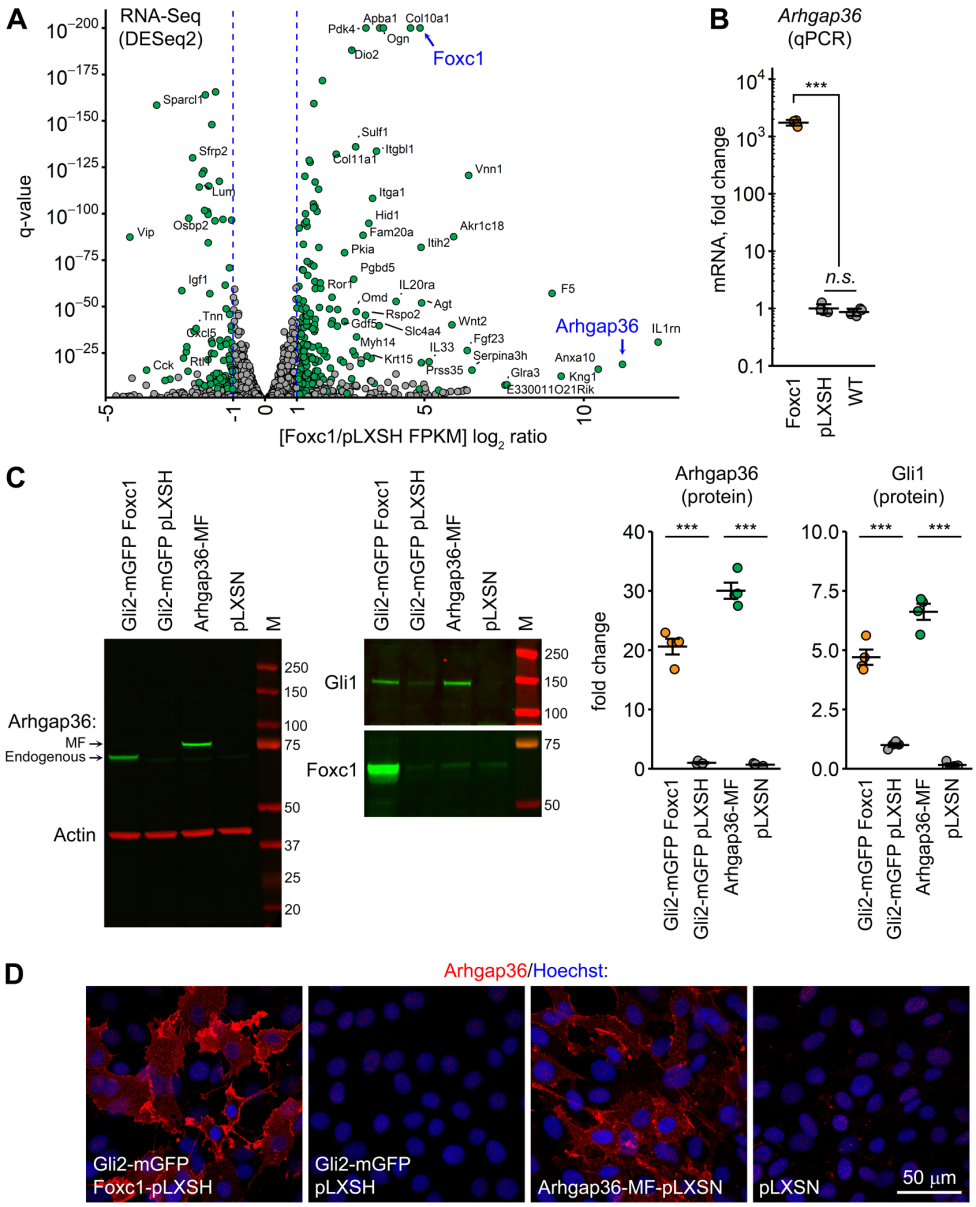
Foxc1 induces expression of Arhgap36 and activates Hedgehog signaling. (**A**) Volcano plot depicting in green differentially expressed genes with Foxc1 expression in NIH3T3 cells. (**B**) Confirmation of the robust Foxc1-induced increase in Arhgap36 mRNA in NIH3T3 cells by qPCR. (**C**) Foxc1 drives endogenous Arhgap36 protein expression, and that of Gli1, in NIH3T3-Gli2-mGFP cells to comparable levels of Myc-FLAG Arhgap36 ectopically expressed in parental NIH3T3 cells. (**D**) Immunofluorescence imaging demonstrates strong endogenous Arhgap36 expression, with the membrane staining in Foxc1-expressing cells recapitulating that of ectopically expressed Arhgap36-MF protein [RNA-seq: *n* = 3; quantitative western blots: *n* = 4 replicates; MF denotes Myc-FLAG tagged Arhgap36]. Figure 1—source data 1.PDF file containing original western blots for Figure 1, indicating the relevant bands and treatments. Figure 1—source data 2.Original files for western blot analyses displayed in Figure 1.

To discern pathways by which Foxc1 may contribute to tumorigenesis, *Foxc1* was stably overexpressed in NIH3T3 fibroblasts. A retroviral construct was used that recapitulated the high levels of FOXC1 expression observed in a range of malignancies (Havrylov et al., 2024). After RNA-sequencing, analysis using a stringent false discovery rate (*q* ≤ 0.01; log_2_fold change ≥1.0) defined a set of differentially expressed genes that were each distinguished by two independent bioinformatic workflows (DEseq2 and Cufdiff; Supplementary file 1). The majority were upregulated by Foxc1 expression (*n* = 192 genes; total 285; Figure 1A). Gene-set enrichment analysis demonstrated significant enrichment for cancer invasiveness phenotypes and malignancies that importantly included neural crest-associated malignancies such as melanoma and neuroblastoma (EMT p = 4.8 × 10^–11^; malignancies p = 10^–3^ to 10^–4^; Supplementary file 1). Also enriched were phenotypes either induced by *FOXC1* mutation (glaucoma and abnormal intraocular pressure, p ≤ 4.7 × 10^–4^) (Nishimura et al., 1998; Mears et al., 1998) or present in *Foxc1^−/−^* murine mutants (abnormal skeletal morphology and renal anomalies, p = 0.05 × 10^–3^) (Kume et al., 1998; Hong et al., 1999; Kume et al., 2000). Finally, genes relevant to Foxc1’s biological functions (angiogenesis, endochondral ossification, and hair follicle development) (Yoshida et al., 2015; Almubarak et al., 2024; Kume et al., 2001; Seo et al., 2012; Siegenthaler et al., 2013; Lay et al., 2016; Bhakuni et al., 2024; Wang et al., 2016) were also enriched in relevant cell types (vasculature p = 2.1 × 10^–5^; neural crest p = 3.0 × 10^–4^; Supplementary file 1). Together, these results support the differentially expressed genes being representative of Foxc1 functions in development and disease.

20% of the significantly dysregulated genes are implicated in Hh signaling (62 of 285) either as regulators or targets of the pathway (Supplementary file 1). Besides a cytokine that regulates inflammation and immunity (Interleukin Receptor 1 antagonist), Arhgap36 mRNA was the most upregulated in the RNA-sequencing dataset (Figure 1A). To test whether Foxc1 consistently induces Arhgap36 expression in mesenchymal-derived cells, qPCR measurements were performed and strong induction of Arhgap36 mRNA observed after Foxc1 overexpression in several relevant cell lines [C2C12 (murine myoblast) 10^5^-fold, ATDC5 (chondrogenic) 10^2^-fold, NIH3T3 10^3^-fold increase; Figure 1B and Appendix 1—figure 1]. Moreover, in a Hh signaling reporter cell line (NIH3T3-Gli2-mGFP) (Havrylov et al., 2024; Kim et al., 2009), Foxc1 overexpression induced Arhgap36 protein expression at levels comparable to ectopically expressed Myc-FLAG tagged Arhgap36 (Figure 1C). Both Foxc1 overexpression and ectopically expressed Arhgap36 elevated protein expression of Gli1, a terminal effector and major readout of Hh pathway activity (Figure 1C). Notably, the cellular localization patterns of Foxc1-induced endogenous and ectopically expressed Arhgap36 are similar (Figure 1D, Appendix 1—figure 2A). Collectively, these data demonstrate Foxc1’s ability to induce Arhgap36 expression and activate Hedgehog signaling.

**Figure 2.**
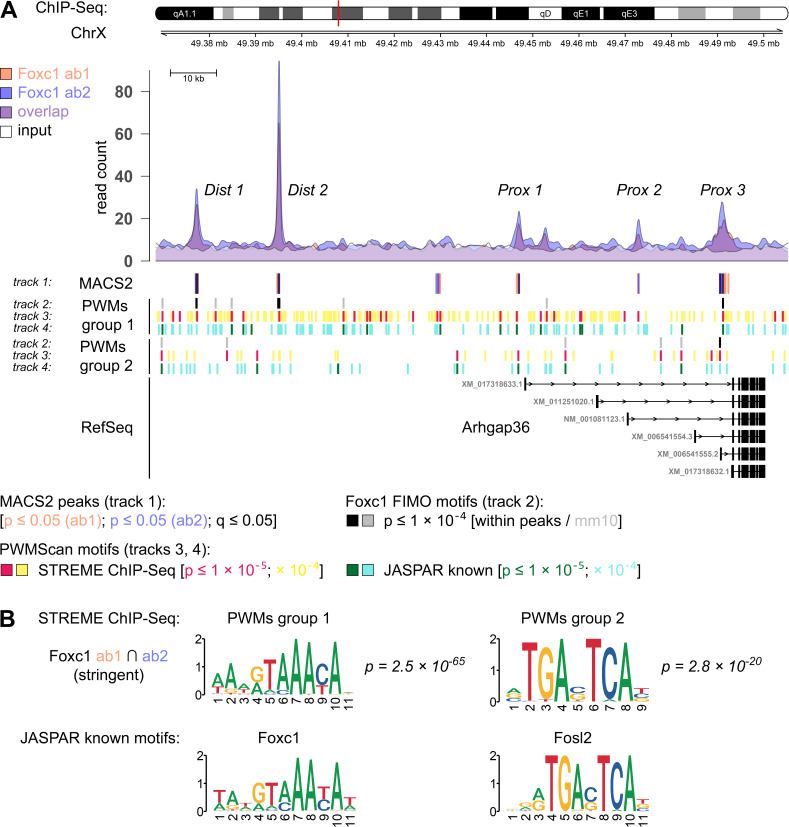
ChIP-seq identification of Foxc1-binding sites at the Arhgap36 locus. (**A**) ChIP sequencing with independent anti-Foxc1 antibodies revealed substantial peak overlap for both ChIP samples, consistent with high antibody specificity. Peak calling identified five significant ChIP signal regions within ±100 kb of *Arhgap36* [2 distal, 3 proximal; *q*-value ≤0.05]. (**B**) Within these Foxc1 ChIP peaks, the discovery algorithm STREME identified two major groups of significantly enriched motifs [p = 2.8 × 10^–20^, 2.5 × 10^–65^]. The group 1 position weight matrices are highly similar to known Foxc1 motifs, while the group 2 PWMs very closely resemble the heptanucleotide recognition sequence bound by Fos–Jun transcription factor dimers [most prominently Fosl2]. The distribution of both PWM groups in the vicinity of Arhgap36 is shown on plot A.

To characterize the functional interaction between the transcription factor Foxc1 and Arhgap36, whole genome ChIP-sequencing (ChIP-seq) was performed on Gli2-mGFP NIH3T3 cells stably expressing *Foxc1*. Two independent Foxc1 antibodies were used, together with an isotype-matched control immunoglobulin as control for Foxc1 peak specificity. The number of uniquely mapped 60 bp reads (Input >5.3 × 10^8^, Foxc1-ChIP 4.8 × 10^8^) corresponds with deep sequencing coverage. Examination of chromatin accessibility demonstrates that, with the exception of embryonic stem cells, the Arhagp36 locus is inactive (Appendix 1—figure 3), an observation consistent with Arhgap36’s highly restricted tissue expression. Within a 200-kb interval encompassing the Arhgap36 locus, five regions displayed strongly overlapping ChIP-seq peaks with both anti-Foxc1 antibodies (Figure 2A). Three (Prox-1 to Prox-3) were 0.2–2.5 kb from the transcription start sites, while two (Dist-1, Dist-2) were 55 and 71 kb upstream (Figure 2A). Two amplicons were selected within each peak, and ChIP-qPCR demonstrated strongly increased Foxc1 signal at each peak, relative to DNA precipitated using a non-specific normal IgG control (Appendix 1—figure 4). Two groups of position weight matrices were significantly enriched in the bulk ChIP-seq peak data (STREME sequence motif discovery algorithm) (Figure 2B, Appendix 1—figure 4). The first comprised consensus sequences closely resembling known Foxc1-binding motifs (p = 10^–41^ to 10^–65^), while the second corresponded closely with that of Fos–Jun transcription factor dimers (p = 10^–13^ to 10^–20^, Figure 2B, Appendix 1—figure 5). Each of the five ChIP-seq peaks contains the identified Foxc1 motifs, supporting Arhgap36 being a direct transcriptional target of Foxc1.

**Figure 3.**
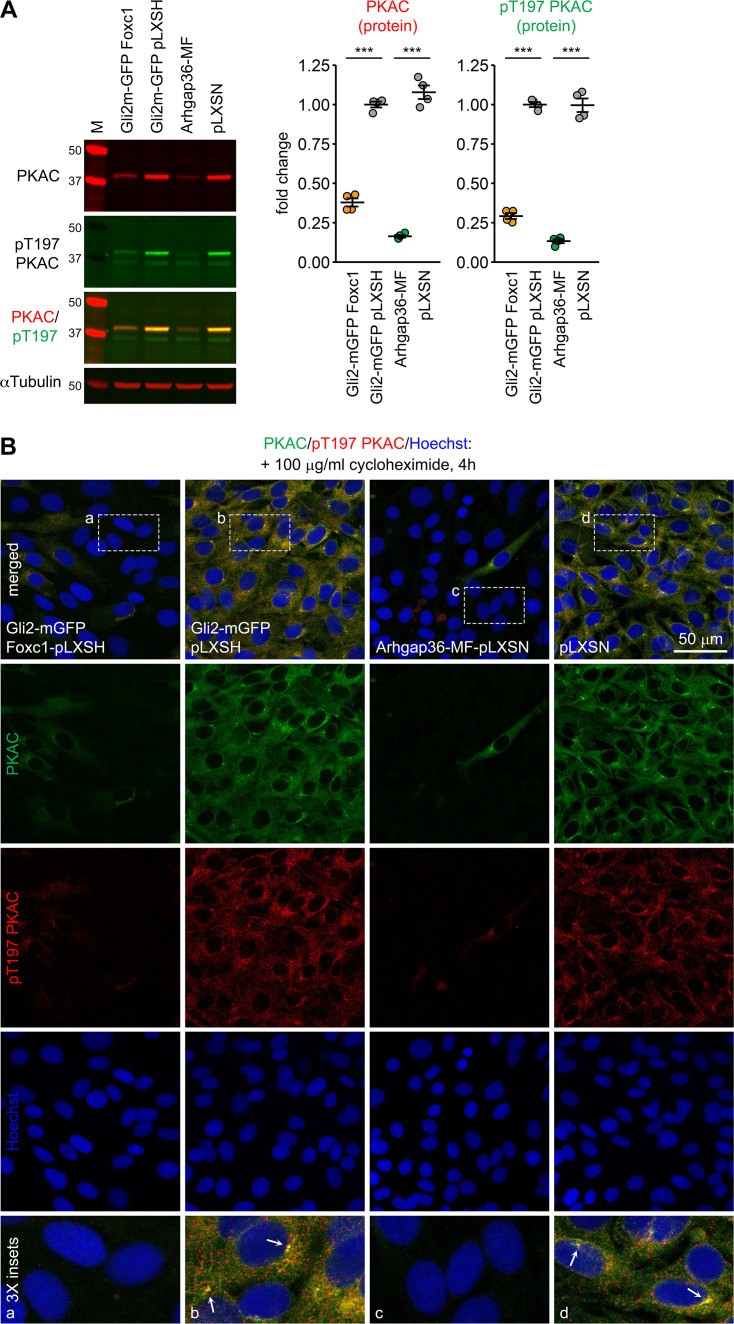
Foxc1-induced Arhgap36 reduces levels of protein kinase A catalytic subunit (PKAC). (**A**) Foxc1 expression in Gli2-mGFP NIH3T3 cells strongly reduces PKAC, and catalytically active pT197 PKAC, to comparable levels to those observed with ectopic expression of Arhgap36-MF. Quantification shows >2-fold reduction of PKAC/pT197 PKAC protein levels [western blots: *n* = 4 replicates]. (**B**) Immunofluorescent staining demonstrates equivalent reductions in PKAC/pT197 PKAC signal in the cytoplasm and at the basal bodies of cells expressing either Foxc1 or ectopic Arhgap36-MF [dashed box: 3x insets, basal body: white arrows]. Figure 3—source data 1.PDF file containing original western blots for Figure 3, indicating the relevant bands and treatments. Figure 3—source data 2.Original files for western blot analyses displayed in Figure 3.

We next investigated in a Hh reporter line (Havrylov et al., 2024) if Foxc1 overexpression recapitulated Arhgap36’s augmentation of Hh signaling, via downregulation of PKA. As expected, ectopic expression of Foxc1 depleted the level of PKAC, PKA’s catalytic subunit (63% reduction, p = 1.4 × 10^–7^; Figure 3A). Foxc1 overexpression also reduced phosphorylation of threonine 197 in PKAC’s activation loop, a residue essential to PKA enzymatic function and protein stability (Steinberg et al., 1993; Cheng et al., 1998). This *~*70% depletion of pT197 PKAC (p = 1.3 × 10^–8^) is analogous to the effect of ectopic Arhgap36 expression in parental NIH3T3 cells (Figure 3A). Immunofluorescent staining demonstrated in cells with ectopic Foxc1 or Arhgap36 expression the near complete absence of PKAC and pT197 PKAC in the cytoplasm, especially at the basal body, where compartmentalized PKA-dependent control of Hh pathway occurs (Figure 3B). Notably, in a transduced cell population with heterogeneous Foxc1 expression, PKAC signal is almost entirely depleted from cells with a high Foxc1 nuclear signal, demonstrating an inverse correlation between levels of PKAC and nuclear Foxc1 immunostaining (Appendix 1—figure 2B). Similar results were observed in a second cell model, pre-adipocyte cells (3T3-L1) where expression of Foxc1-induced expression of Arhgap36 (albeit at lower levels) and a 40–50% reduction in protein levels of PKAC and pT197 PKAC (Appendix 1—figure 2C, D). Direct overexpression of Arhgap36 led to strong downregulation of PKAC. These data, from two cell-based systems, demonstrate that Foxc1 expression depletes PKAC, the enzyme essential for promoting repressor forms of Gli, which regulate Hh target gene expression.

**Figure 4.**
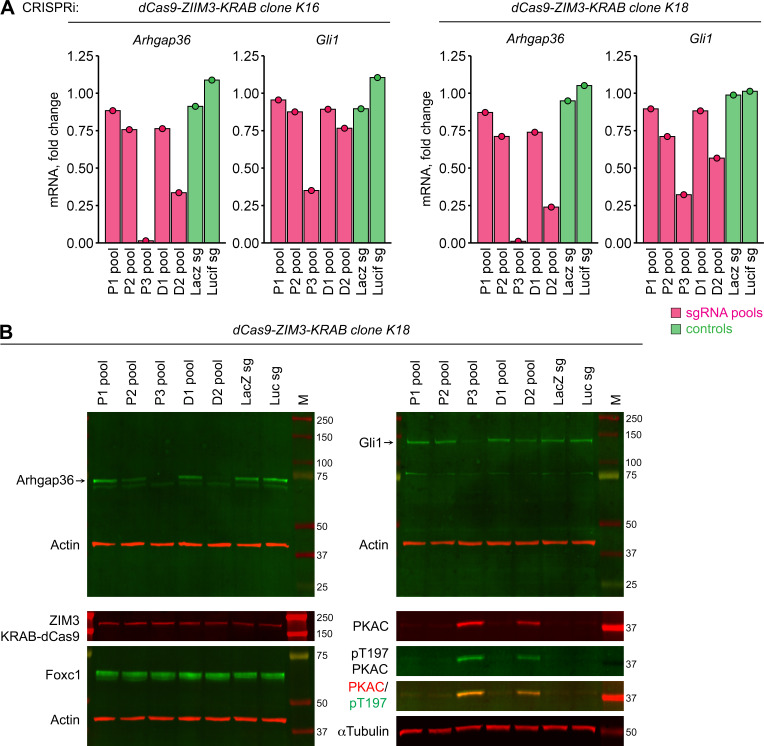
CRISPRi at potential Foxc1-binding sites diminishes Arhgap36 expression and Hedgehog activity. (**A**) In independent CRISPRi-competent clonal cell lines, pools of guide RNAs targeting the Prox-3 and Dist-2 regions robustly reduced Arhgap36 and Gli1 mRNA expression. (**B**) The reduced Arhgap36 and Gli1 protein expression with CRISPRi sgRNA targeting implicates the same two ChIP-seq identified peaks in Foxc1’s transcriptional control of the Arhgap36 locus. Figure 4—source data 1.PDF file containing original western blots for Figure 4, indicating the relevant bands and treatments. Figure 4—source data 2.Original files for western blot analyses displayed in Figure 4.

We next examined using CRISPR interference whether steric repression of the five Foxc1-binding loci affected Arhgap36 transcription. Two clones of NIH3T3-Gli2-mGFP cells that overexpress Foxc1 were created that each stably expressed an inactive form of Cas9 fused with the Krüppel-associated box (KRAB) repressor domain of ZIM3 protein. Recruitment of ZIM3 KRAB-dCas9 to each of the five Foxc1-binding regions by retroviral delivery of five separate pools each comprising three to four single-guide RNAs altered Arhgap36 expression, although to varying degrees (Figure 4A, Appendix 1—figure 6). The sgRNA pools targeting Prox-3 and Dist-2 reduced Arhgap36 mRNA expression by 98–99% and 67–76%, respectively (Figure 4A). In both cases, reduced expression of Arhgap36 was associated with lower levels of Gli1 mRNA, and the degree of Arhgap36 mRNA reduction correlated with the decrease in Gli1 mRNA levels. Western immunoblot analysis confirmed that silencing either the Prox-3 or Dist-2 regions decreased Arhgap36 protein expression. This, in turn, correlated with an increase in PKAC and pT197 PKAC levels and a reduction in Gli1 protein levels (Figure 4B). Since these data support Prox-3 and Dist-2 functioning, respectively, as promoter and distal enhancer for murine *Arhgap36*, evolutionary conservation of the Foxc1-binding loci was assessed. Multiple alignment across 60 vertebrates (Multiz) and measurements of evolutionary conservation (PhastCons) demonstrated that Prox-3 and Dist-2 are both conserved in placental mammals, but not in marsupial mammals, nor in other vertebrates (Appendix 1—figure 7). Multiple potential binding motifs for Foxc1 and Fos–Jun family transcription factors were identified in both regions, as illustrated by 16 Foxc1-binding motifs in both Prox-3 (8 motifs) and Dist-2 (8 motifs) of the murine locus that were conserved or partially conserved in humans (Appendix 1—figure 7; Supplementary file 1). Consistent with this finding, luciferase reporter assays demonstrate that mutation of predicted Foxc1-binding motifs in the Prox-3 and Dist-2 regions abrogates Foxc1-dependent transcriptional activity (Appendix 1—figure 8). Taken together, the data presented support the Prox-3 and Dist-2 regions functioning as cis-regulatory elements for murine *Arhgap36* and other placental mammals, including humans.

**Figure 5.**
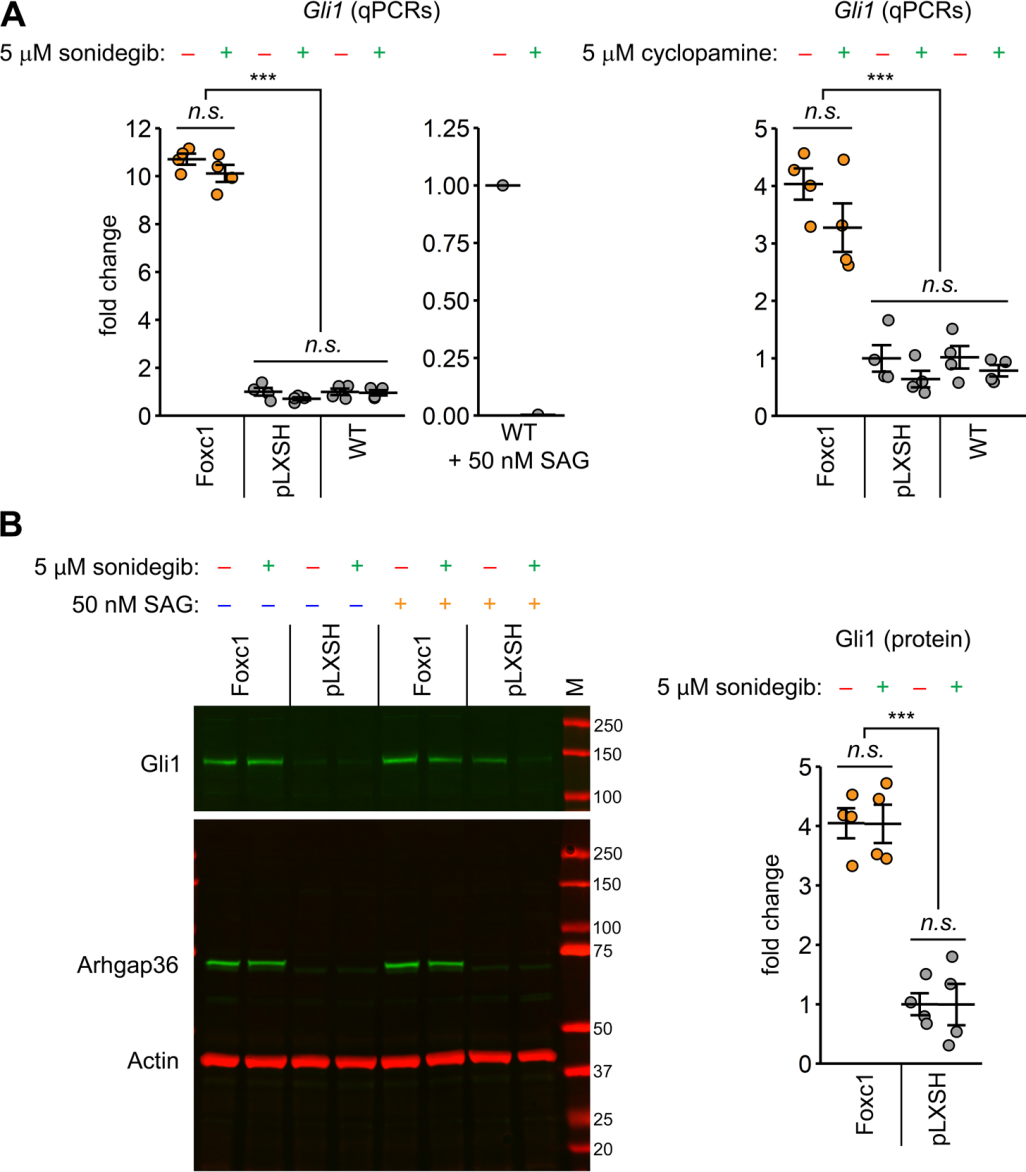
Foxc1-induced Hh signaling has reduced dependence on *Smoothened*. (**A**) Elevated levels of Gli1 mRNA in Foxc1-expressing NIH3T3 cells are resistant to inhibition by the *Smoothened* antagonists sonidegib and cyclopamine. Wild-type NIH3T3 cells stimulated with *Smoothened* agonist (SAG) and treated with sonidegib provide a control for inhibitor efficiency. (**B**) Resistance to *Smoothened* inhibition is supported by the elevated Gli1 protein levels in Foxc1-expressing Gli2-mGFP NIH3T3 cells treated with sonidegib. Note that expression of Foxc1 induces comparable Gli1 protein levels to vector control cells [pLXSH] treated with SAG; and that levels of Arhgap36 protein itself are unaffected by either sonidegib or SAG treatment [qPCRs, quantitative western blots: *n* = 4 replicates]. Figure 5—source data 1.PDF file containing original western blots for Figure 5, indicating the relevant bands and treatments. Figure 5—source data 2.Original files for western blot analyses displayed in Figure 5.

Since PKA depletion de-represses Hh signaling, the pathway activation is predicted to become less dependent on Hh ligand, which pharmacologically would manifest as tumor resistance to inhibition of *Smoothened* (*Smo*). To test whether Foxc1 expression induces such ligand-independent Hh signal transduction, Gli1 levels were measured with and without two *Smoothened*-specific antagonists (Sonidegib and Cyclopamine). The results revealed that the Foxc1-induced increase of Gli1 mRNA and protein levels is resistant to inhibition by either antagonist (Figure 5A, B). Furthermore, the Foxc1-induced resistance to Sonidegib is phenocopied by the ectopic expression of Arhgap36 (Appendix 1—figure 9A). In both cases, Gli1 upregulation, whether by Foxc1 or by Arhgap36 overexpression, is not significantly reduced by co-treatment with Sonidegib. Indeed, Foxc1 overexpression leads to a similar level of Gli1 upregulation in NIH3T3 cells as treatment with a *Smoothened* agonist (SAG), and co-treatment of cells with SAG does not significantly enhance Gli1 levels beyond Foxc1-induced upregulation alone (Figure 5B). Consequently, these data demonstrate that Foxc1 induces strong non-canonical activation of Hh signaling, a characteristic of multiple malignancies, including advanced and metastatic tumors.

**Figure 6.**
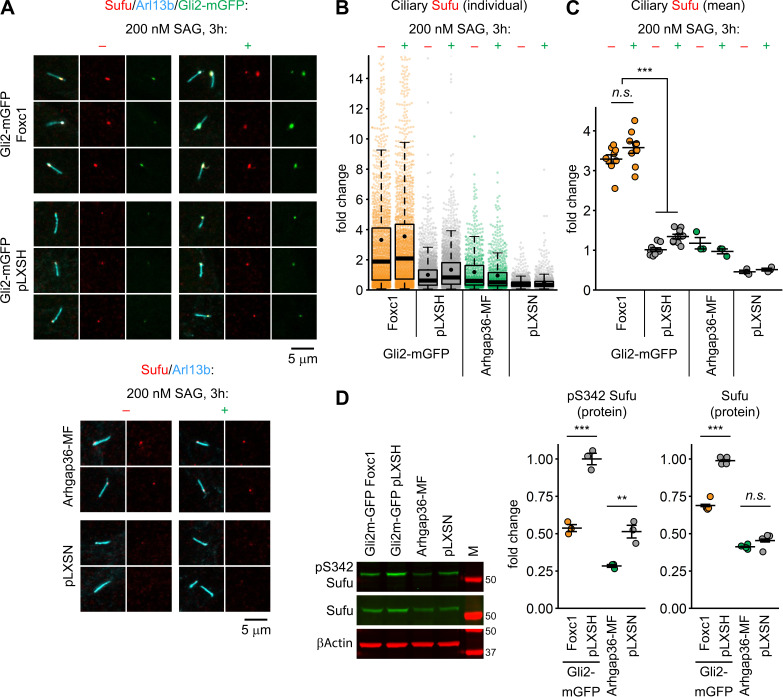
Foxc1 promotes ciliary accumulation and decreases phosphorylation of Sufu. (**A**) Representative immunofluorescence images demonstrate increased Sufu accumulation at axonemal tips of Foxc1-expressing cells and NIH3T3 cells that express Myc-FLAG Arhgap36. Note, SAG stimulation per se does not substantially affect ciliary accumulation of Sufu. (**B**) Distribution of Sufu intensity in individual cilia (**C**) Mean ciliary Sufu intensity values. These demonstrate that the ciliary Sufu signal in cells expressing Foxc1 and separately Arhgap36 is substantially increased relative to empty vector controls [pLXSH and pLXSN; *n* = 9 combined experiments]. (**D**) Decreased phosphorylation of Sufu at the S342 residue in Gli2-mGFP NIH3T3s expressing Foxc1 and NIH3T3 cells expressing Myc-FLAG Arhgap36, relative to vector controls. Expression of Foxc1 also significantly impacts the total protein levels of Sufu, in contrast to the non-significant effect of Arhgap36 [quantitative western blots: *n* = 4 replicates]. Figure 6—source data 1.PDF file containing original western blots for Figure 6, indicating the relevant bands and treatments. Figure 6—source data 2.Original files for western blot analyses displayed in Figure 6.

PKA’s roles regulating the Hh pathway extend beyond repressing Gli activity. PKA also phosphorylates key serine and threonine residues within Sufu. This inhibits ciliary trafficking of Sufu–Gli complexes, resulting in retention of Sufu at the tip of the primary cilium (Chen et al., 2011; Tukachinsky et al., 2010). We found that in addition to substantially increased axonemal tip accumulation of Gli2-mGFP (Figure 6A, Appendix 1—figure 9B, C), expression of Foxc1 also increases Sufu levels at the tip of the axoneme threefold (p ≤ 2 × 10^–10^) while a similar twofold increase is induced by ectopic expression of Arhgap36 (Figure 6A–C). The status of Sufu’s PKA-dependent phosphorylation sites is known to influence Sufu activity. Two of these adjacent serine residues comprise a classical dual phosphorylation site for PKA (pS346) and GSK3β (pS342) (Chen et al., 2011). Consequently, the ~50% reduction in levels of Sufu pS342 after Foxc1 (p = 6.4 × 10^–5^) or Arhgap36 expression (Figure 6D) provides further support for Hh pathway regulation by Foxc1. Together these data demonstrate that the effects of Foxc1 expression on Hh signal transduction involve several core regulators of Hh pathway activity.

**Figure 7.**
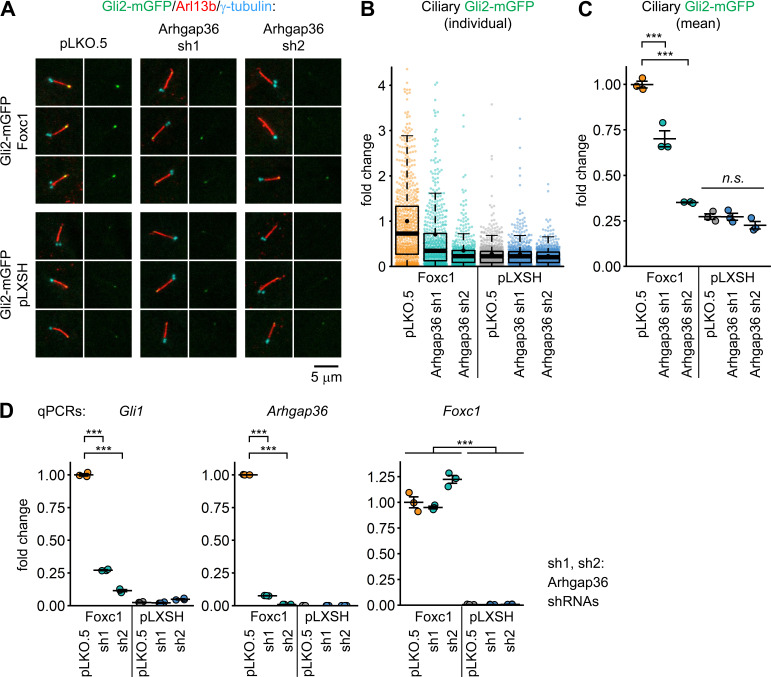
Arhgap36 is required for Foxc1-induced activation of Hh signaling. (**A**) Immunofluorescence images demonstrate that two Arhgap36-targeting shRNAs each substantially reduce axonemal tip accumulation of Gli2 in cells that express Foxc1. (**B, C**) Individual and mean ciliary Gli2 intensity values demonstrate the decrease of ciliary Gli2 signal with Arhgap36 shRNA inhibition [*n* = 3 combined experiments]. (**D**) qPCR analyses demonstrate a strong decrease in Gli1 mRNA levels in Foxc1-expressing cells treated with Arhgap36 shRNAs, relative to pLKO.5 control. Note, concordant Gli1 and Arhgap36 expression levels across all conditions.

The presented data support a model by which Foxc1-dependent Arhgap36 expression attenuates PKA activity and Sufu function. To verify that the observed changes in Sufu and Gli2 ciliary accumulation induced by Foxc1 overexpression are mediated by Arhgap36, shRNA silencing of Arhgap36 was performed in Foxc1-expressing Gli2-mGFP cells (Figure 7). Two different Arhgap36-targeting shRNAs each substantially diminished ciliary Gli2-mGFP accumulation (shRNA1: 35%, shRNA2: 64% reduction; p = 4.9 × 10^–7^ and 4.5 × 10^–10^; Figure 7A–C) while Gli2-mGFP levels in control cells that express very low levels of Arhgap36 were unchanged. The decrease in axonemal Gli2-mGFP correlated with the reduction in Gli1 mRNA (Figure 7D), and the shRNA that more efficiently inhibited Arhgap36 induced a greater decrease in both Gli2-mGFP accumulation and Gli1 expression. The specificity of Arhgap36 inhibition is apparent from marked reductions in Gli1 and Arhgap36 mRNA expression, while levels of Foxc1 were unaltered (Figure 7D). Analysis of Gli1 expression in parental Foxc1-expressing NIH3T3 cells treated with the same Arhgap36-targeting shRNA recapitulated the prior findings (Appendix 1—figure 10). Taken together, these data demonstrate that the effects of Foxc1 expression on Hh pathway activity are Arhgap36-mediated.

**Figure 8.**
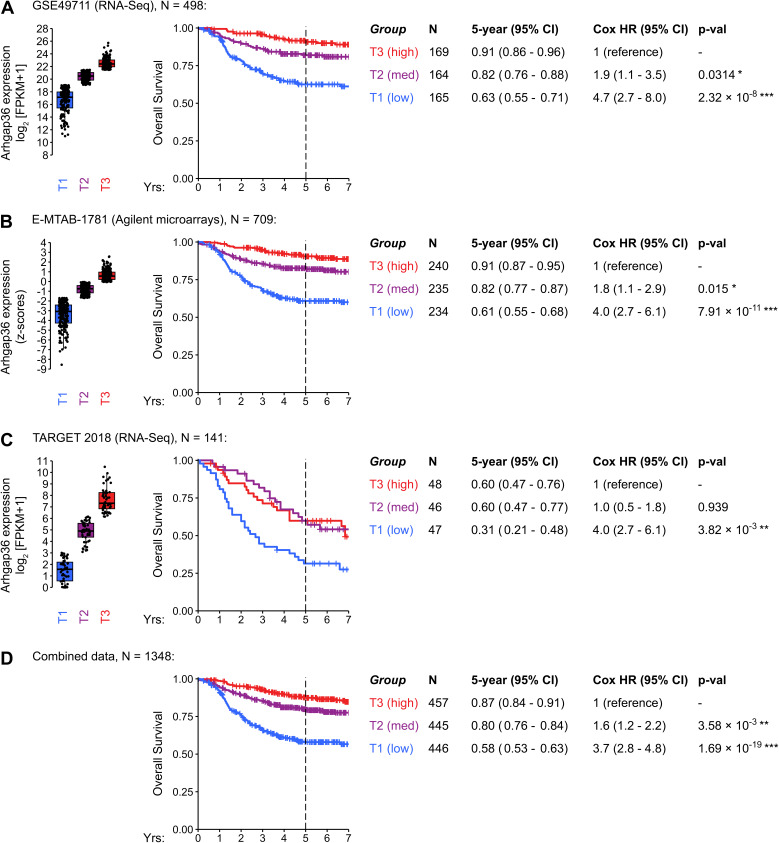
Overall survival of neuroblastoma patients stratified by Arhgap36 expression levels. (**A–C**) Kaplan–Meier plots show poor overall survival with low Arhgap36 expression across three independent neuroblastoma datasets. The significant reduced 5-year survival for cases with low levels of Arhgap36 expression is evident from the range of hazard ratios (2.7–8.0). Panel (**D**) shows the same analysis once the data are merged into a single dataset comprising *n* = 1348 individuals (HR 2.8–4.8) [cases stratified into terciles of Arhgap36 expression as shown in boxplots: first tercile (T1) ‘low’; second (T2) ‘medium’; third (T3) ‘high’].

To test the oncologic relevance of the Foxc1–Arhgap36 relationship, *ARHGAP36* mRNA levels were first surveyed in cancer expression datasets [CCLE, TCGA, TARGET, PCAWG (Supplementary file 1)]. This revealed high expression in a common pediatric malignancy, neuroblastoma, as well as specific CNS, breast, lung, and neuroendocrine tumors. Due to neuroblastoma’s neural crest origin and Foxc1’s roles in this stem cell population, we focused on neuroblastoma data. Survival was evaluated in three independent neuroblastoma patient cohorts using Kaplan–Meier analyses after stratification into terciles of high, medium, and low ARHGAP36 mRNA expression. The highest tercile of *ARHGAP36* expression correlated with a favorable overall survival compared to the lowest: GSE49711, 91 vs 63% 5-year survival; E-MTAB-178191, 91 vs 61%; TARGET study, 60 vs 31%, (Figure 8A–C; SEQC/MAQC-III Consortium, 2014; Oberthuer et al., 2015; Ma et al., 2018). The effect was consistent across all three cohorts, despite differences in cancer staging – the first two cohorts primarily comprise patients with early disease, while TARGET mainly consists of advanced or metastatic neuroblastoma cases. Merging the data into a single dataset of 1348 patients narrowed the confidence intervals to provide more precise estimates. The highest tercile of *ARHGAP36* expression was associated with 87% 5-year survival (range 86–92%) in contrast to 58% (53–63%) for the lowest tercile (p = 1.7 × 10^–19^). Illustrating these findings in the context of *MYCN*, whose amplification represents the primary genetic marker for high-risk and prognostically poor neuroblastoma, high ARHGAP36 expression was associated with an 89% 5-year survival compared to 37% with *MYCN* amplification (Appendix 1—figure 11).

## Discussion

Here, we show that the PKA antagonist Arhgap36 is a central gene dysregulated by Foxc1 overexpression, and that this linkage provides new perspectives in Foxc1’s contributions to malignancy. Foxc1’s transcriptional regulation of Arhgap36, and the consequent inhibition of PKA, alters the equilibrium between full-length transcriptional GLI activators and truncated transcriptional repressors, weakening regulation and increasing activity of a pathway fundamental to development. It is therefore logical that a transcription factor identified to enhance Arhgap36 expression is one that induces Hh activity in multiple tissues during development (Yoshida et al., 2015; Almubarak et al., 2024; Havrylov et al., 2024). In some contexts, Hedgehog functions as a morphogen that confers positional information, with a gradient of activity specifying individual cell types and behaviors required for the establishment of complex tissue patterns (Heemskerk and DiNardo, 1994; Helms et al., 1994; Martí et al., 1995). Increased gene dosage from *FOXC1* copy number variation causes phenotypes indicative of impaired Hedgehog signaling, including cerebellar hypoplasia and anomalous facial skeletal development (Aldinger et al., 2009; French et al., 2014; Haldipur et al., 2014; Haldipur et al., 2017). Consequently, Foxc1’s ability to induce Arhgap36 expression establishes a new mechanism by which the transcription factor may modulate Hh pathway activity. This has particular relevance for oncology, where dysregulated Hh expression makes major contributions and larger fold increases in FOXC1 expression occur.

A second finding concerns the breadth of alteration to Hh signaling. Foxc1’s induction of Arhgap36 depletes an evolutionarily conserved negative regulator of Hh signaling (PKA) from the cytoplasm and base of the primary cilium. Such wide loss of PKA activity should release Gli transcription factors from phosphorylation-induced direct inhibition (in case of Gli2) and proteolytic processing (in case of Gli2 and Gli3). In parallel, Foxc1-induced depletion of PKAC predictably dysregulates the PKA substrate Sufu: a second major negative regulator of the Hh pathway, whose roles encompass development, disease, and notably tumor suppression. Sufu acts as a scaffold between PKA and Gli proteins that facilitates their phosphorylation and proteolytic processing, while also directly inhibiting the transcriptional activity of active Gli forms (Tukachinsky et al., 2010). The reduced phosphorylation of Sufu and increased translocation of Sufu and Gli2 to the tip of the cilium represent alterations to fundamental features of Hh signal transduction. As Arhgap36 acts independently of Smo (Rack et al., 2014), another consequence of Foxc1-driven Arhgap36 expression observed in our experiments is acquired resistance of Hh pathway activity to Smo antagonists. Consequently, by altering the activity of PKA, Foxc1 overexpression impairs central elements of the Hh pathway, resulting in increased transcription of Hh-target genes and, via augmented Smo-independent activity, tumor resistance to *Smoothened* antagonists. Collectively, such properties are consistent with the overexpression of FOXC1 in multiple tumors being associated with more aggressive cancer phenotypes, drug resistance, and metastasis.

Forkhead box transcription factors’ ability to access areas of closed chromatin is mediated by a conserved winged-helix DNA-binding domain that is structurally similar to histones H1 and H5 (Cirillo et al., 1998). This capacity to directly bind DNA targets in condensed chromatin enables pioneer factors to activate enhancers and, by opening condensed chromatin, provide access to transcription factors that lack pioneering ability. The closely correlated ChIP-seq peaks identified in this study, which contain consensus sequences for Foxc1 and Fos–Jun dimers, support coordinated transcription factor binding at the Arhgap36 locus. The consistent co-localization of these transcription factors’ binding motifs, including in other placental mammals, accords with the enrichment of FOSL2 and JUNB motifs at the FOXC1 ChIP-seq peaks noted in a previous study (Ramachandran et al., 2024). Together, such data support a model in which the overexpression of FOXC1, by initiating local opening of chromatin (Xu et al., 2021), makes the ARHAGP36 locus accessible to other transcription factors to induce gene expression. Such combinatorial control of Arhgap36’s transcriptional regulation is predicted to have heterogeneous effects on Hh pathway activity (Appendix 1—figure 12), varying in tissue and cell-specific manners and being determined by levels of FOXC1 and FOS–JUN dimers.

FOXC1’s induction of Hh signaling via ARHGAP36 and PKA inhibition is expected to modify processes in both development and disease. Since our data were derived with retroviral constructs, where 10- to 20-fold increases in Foxc1 protein levels reiterate overexpression in FOXC1-associated tumors (Ray et al., 2010), we evaluated the relevance of the Foxc1–Arhgap36 interaction in human malignancy. Analyses in neuroblastoma, a heterogeneous, life-threatening, and common pediatric malignancy that originates from sympathoadrenal neural crest progenitors, revealed higher levels of ARHGAP36 expression were comparatively protective. The 2.8- to 8-fold greater 5-year survival identified in three independent patient cohorts suggests ARHAGP36 may represent an informative prognostic factor. Because neuroblastoma treatment regimens frequently impact the development of surviving children (Sainero-Alcolado et al., 2024), the ability to stratify by prognosis may facilitate treatment choice and minimize morbidity for a portion of patients. Although the mechanism(s) mediating this effect remain to be explored, this observation is consistent with the requirement of Hh activity for many tumors to proliferate (Dahmane et al., 1997; Duman-Scheel et al., 2002) and for maintenance of stem cell self-renewal, including that of cancer stem cells (Beachy et al., 2004).

In summary, dysregulated expression of *FOXC1* is well-implicated in malignancy, with expanding evidence that elevated *FOXC1* levels are associated with increased mortality in breast and hepatic cancer and malignancies as diverse as AML and low-grade glioma (Somerville et al., 2015; Ray et al., 2011; Xia et al., 2013; Cao et al., 2019). In contrast with such progress, determining how FOXC1 promotes malignancy has proved more elusive. This work establishes that overexpression of FOXC1, at levels consistent with FOXC1-associated malignancies, transcriptionally activates Arhgap36 expression which in turn dysregulates multiple fundamental aspects of Hh pathway activity. The presented data further demonstrate that ARHGAP36 levels are predictive of 5-year survival in a common pediatric malignancy and identify a biological characteristic that merits further investigation. Overall, this study provides additional mechanistic insight into FOXC1’s capacity for stimulating malignancy.

## Materials and methods

### Plasmids, antibodies, and other reagents

Retroviral vector for stable expression of MycFLAG-tagged mouse *Arhgap36* was created by InFusion HD subcloning into pLXSN backbone. pLX303-ZIM3-KRAB-dCas9 and pLCKO plasmids for ZIM3 KRAB-dCas9 CRISPRi platform (Alerasool et al., 2020) were obtained from Addgene. The sgRNA sequences targeting various regions within the Arhgap36 locus were generated by cloning of annealed primer duplexes into pLCKO vector using InFusion HD. All lentiviral shRNA plasmids from TRC mouse MISSION shRNA libraries were purchased from Millipore Sigma. Retroviral pLXSH vector for stable expression of *Foxc1* ORF was previously described (Havrylov et al., 2024). Comprehensive lists of plasmids, antibodies, primers, as well as other reagents used in the study are provided in Supplemental methods.

### Cell lines

NIH3T3 mouse fibroblasts and Gli2-mGFP NIH3T3 reporter cells stably expressing *Foxc1* ORF or pLXSH vector control were generated and maintained in the presence of Geneticin and/or Hygromycin (Havrylov et al., 2024). NIH3T3 cells stably expressing Arhgap36 MycFLAG-tagged protein or empty pLXSN vector control were generated by transduction with retroviral particles, followed by selection in 1200 µg/ml Geneticin. Resulting cell populations were maintained in media containing 400 µg/ml Geneticin. NIH3T3 and Gli2-mGFP NIH3T3 Foxc1/pLXSH cell lines stably expressing *Arhgap36* shRNAs were generated via lentiviral transduction and next selection in 2.5 µg/ml Puromycin. All NIH3T3 derivative cell lines were cultured in DMEM supplemented with 10% FBS. All cells were kept at 37°C in 5% CO_2_, in growth media containing 100 U/ml penicillin and 100 µg/ml streptomycin.

The identity of all the cell lines used in the study (NIH3T3, C2C12, and ATDC5) was confirmed by STR profiling at the Centre for Applied Genomics (SickKids, Toronto, ON). Negative mycoplasma status was periodically confirmed using fluorescent dyes (Hoechst/DAPI).

### RNA-sequencing

RNA was isolated from NIH3T3 cells stably expressing *Foxc1* ORF or empty vector control using RNeasy Plus Mini Kit (QIAGEN). Sample quality was checked using the Agilent 2100 Bioanalyzer to ensure high RNA integrity. Samples were then prepped following the standard protocol for the NEBnext Ultra II Stranded mRNA (New England Biolabs). Sequencing was performed at the BRC Sequencing Core (University of British Columbia, Vancouver, BC) on the Illumina NextSeq500 with paired-end 43 × 43 bp reads. Obtained data were de-multiplexed using Illumina’s bcl2fastq2. De-multiplexed read sequences were then aligned to the *Mus musculus* (mm10) reference sequence and analyzed for differential expression using DESeq2 and Cufflinks, through bioinformatics apps available on Illumina Sequence Hub (https://basespace.illumina.com/).

### ChIP-sequencing

Cross-linked chromatin was prepared and immunoprecipitated from Gli2-mGFP NIH3T3 reporter cells stably expressing *Foxc1* ORF using Simple ChIP Plus Enzymatic Chromatin IP Kit (New England Biolabs), with two different anti-Foxc1 antibodies. Enriched chromatin was reverse-crosslinked, purified, and sequenced together with input control on Illumina MiSeq 300 with paired-end 61 × 61 bp reads. Sequencing was performed at the BRC Sequencing Core (University of British Columbia, Vancouver, BC). Peak calling was done with MACS2 software on Galaxy platform (https://usegalaxy.org/). De novo motif detection in the ChIP peaks was conducted using STREME workflow from MEME Suite (https://meme-suite.org/meme/). Detailed ChIP protocol is provided in Supplemental methods.

### CRISPRi experiments

K16 and K18 cell lines expressing ZIM3 KRAB domain fused with N terminus of dCas9 (clones K16 and K18) used in CRISPRi experiments were derived from Gli2-mGFP *Foxc1*-expressing NIH3T3 cells via lentiviral delivery of ZIM3-KRAB-dCas9 construct, followed by clonal selection in media containing 1.25 µg/ml Blasticidin. Resulting clonal cell lines were maintained in the presence of µg/ml Blasticidin, 80 µg/ml Hygromycin, and 500 µg/ml Geneticin. To achieve CRISPR interference at various regions of *Arhgap36* locus, pools of 3–4 sgRNAs per each targeted peak region identified in ChIP-seq experiments, alongside non-targeting *Lacz* and *Luc* sgRNA controls, were packaged into lentiviral particles and delivered via transduction into K16 and K18 clonal cell lines expressing ZIM3-KRAB-dCas9. Following a week of transient selection for sgRNA expression in 2 µg/ml Puromycin, cells were processed for either mRNA isolation and subsequent qPCR analyses or preparation of cell lysates for western blotting.

### RT-qPCR analyses

Total RNA was isolated with RNeasy Plus Mini Kit (QIAGEN), quantified, and used for cDNA synthesis with Primescript RT Master Mix (Clontech). qPCR reactions were run with TB Green Premix Ex Taq (Tli RNAse H Plus) master mix (Clontech) on LightCycler 96 Instrument and analyzed using LightCycler 96 Application (Roche Life Science). Primer sets are provided in Supplemental methods.

### Quantitative western blotting

Cells were lysed in 1.5% SDS lysis buffer (50 mM Tris pH 7.5, 150 mM NaCl, 1 mM EDTA, 1.5% SDS) supplemented with either Halt Protease Inhibitor Cocktail or Pierce Protease Inhibitor Mini Tablets (Thermo), as well as phosphatase inhibitors (0.5 mM Na_3_VO_4_, 5 mM NaF, 10 mM β-glycerophosphate). Resulting lysates were passed through QIAshredder columns (QIAGEN). Obtained protein samples were normalized using BCA Protein Assay Kit (Thermo), resolved by SDS–PAGE (NuPage 4–12% Bis-Tris gels, Invitrogen), transferred to Immobilon-FL PVDF membranes (EMD Millipore), and blocked with Intercept Blocking Buffer, TBS (Li-Cor). Membranes were next incubated with relevant primary antibodies, followed by IRDye-conjugated secondary antibodies. Resulting membranes were scanned with Odyssey Imaging System (Li-Cor). Protein levels were quantified using Odyssey Application Software (Li-Cor).

### Immunofluorescent staining and quantification

Cells seeded on optic bottom 24-well plates or 8-well slides (Ibidi; Cellvis) were treated as indicated in each experiment and fixed in Dent’s fixative for 30 min at room temperature. Following one wash with 1 ml D-PBS (Thermo Fisher), blocking was performed in a blocking solution (TBS, 0.02% or 0.2% Triton X-100, 2–5% horse serum) for 1 hr at room temperature. Cells were next incubated with primary antibodies diluted in 100–150 µl blocking solution (2% horse serum) per well (see Supplemental methods for dilutions), overnight at 4°C. Next day, cells were washed in TBSTx (TBS with 0.02% or 0.2% Triton X-100) on a rocker platform and incubated with secondary antibodies (1:1000 for all secondary antibodies used) and Hoechst 33258 (2 µg/ml) diluted in 100–150 µl blocking solution, in the darkness, for 1 hr at room temperature. Afterwards, cells were washed TBSTx, then in TBS. Finally, TBS was added to each well and cells were visualized by confocal microscopy. Images were collected using Zeiss LSM 700 laser scanning confocal microscope. Ciliary accumulation of Gli2-mGFP and Sufu, as well as numbers of cleaved-PARP1 positive cells were quantified by automated image analysis using Cell Profiler software (Stirling et al., 2021).

### Omics datasets and expression dataset analyses

RNA-seq and ChIP-seq data generated by this study have been deposited in NCBI’s Gene Expression Omnibus (GEO) and are accessible through GEO Series accession numbers GSE297719 (RNA-seq), GSE297865 (ChIP-seq). Publicly available gene expression datasets for three neuroblastoma patient cohorts were retrieved from NCBI’s GEO (GSE49711; part of GSE47792) (SEQC/MAQC-III Consortium, 2014), EMBL-EBI ArrayExpress (E-MTAB-1781) (Oberthuer et al., 2015), and cBioPortal for Cancer Genomics (Pediatric Neuroblastoma TARGET, 2018) (Ma et al., 2018). To define risk groups for each gene, normalized gene expression values within each dataset were ranked and split at 33.3% and 66.6% percentiles into three groups representing ‘low’ (tercile T1), ‘medium’ (tercile T2), and ‘high’ (tercile T3) expression levels. Subsequent Kaplan–Meier survival analyses and Cox proportional hazards analyses of the resulting data were performed using R language version 4.4.1 (‘survival’, ‘survminer’ packages).

### Statistics

Statistical analyses and plotting were performed using R language version 4.4.1 (The R Project for Statistical Computing). Analyses of statistical significance (p < 0.05) of differences between multiple groups were performed using one- or two-way ANOVA followed up by Tukey’s post hoc test, differences between two groups – using Student’s *t*-test, as indicated for specific experiments. Bar plots and line plots show: individual data points for independent experimental replicates (circles), mean values (horizontal lines) ± SEM (error bars) where applicable. Box-whisker plots (Figures 6B and 7B) show quartiles, median (black horizontal lines), and mean (black dots) values. Boxplots (Figures 8 and Appendix 1—figure 11) show quartiles, median (black horizontal lines) and individual expression data points for each sample. Significance codes ***p < 0.001, **p < 0.01, *p < 0.05.

## Figures and Tables

**Appendix 1—figure 1.**
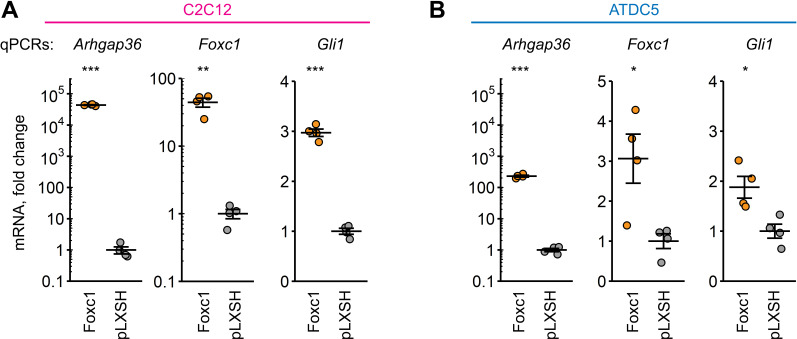
Foxc1 induces expression of Arhgap36 and activates Hedgehog signalling. (**A, B**) Stable overexpression of Foxc1 in C2C12 and ATDC5 cells induces strong increases in Arhgap36, and Gli1, mRNA levels.

**Appendix 1—figure 2.**
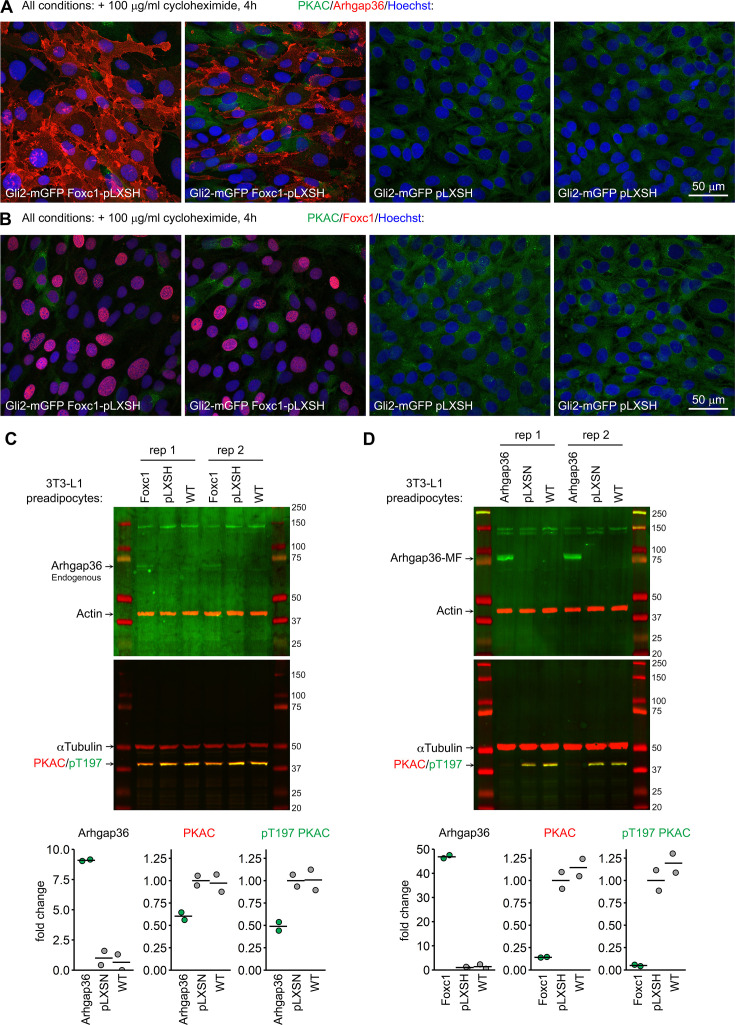
Foxc1-driven Arhgap36 reduces levels of protein kinase A catalytic subunit (PKAC) in Gli2-mGFP NIH3T3 cells and 3T3-L1 preadipocytes. (**A**) Immunofluorescent staining confirms substantial reduction of PKAC signal in Foxc1-expressing Gli2-mGFP NIH3T3 cells. Note correlation between positive Arhgap36 staining and PKAC loss in individual cells. (**B**) Immunofluorescent staining of Foxc1 and PKAC under the same conditions. Note nearly complete loss of PKAC signal in cells with high Foxc1 nuclear signal. (**C**) Foxc1 induced expression of endogenous Arhgap36 in 3T3-L1 cells is accompanied by 40-50% reduction in PKAC/pT197 PKAC protein levels. (**D**) Arhgap36-MycFLAG expression in 3T3-L1 cells strongly reduces PKAC, and catalytically active pT197 PKAC. Appendix 1—figure 2—source data 1.PDF file containing original western blots for Appendix 1—figure 2, indicating the relevant bands and treatments. Appendix 1—figure 2—source data 2.Original files for western blot analyses displayed in Appendix 1—figure 2.

**Appendix 1—figure 3.**
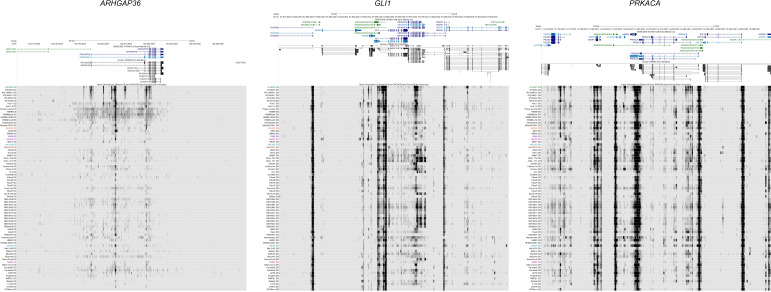
The ARHGAP36 locus is located in a region of predominantly closed chromatin. DNAse I hypersensistivity data from the UCSC genome browser for the 100 kb regions surrounding human ARHGAP36, and GLI1 and PRKACA (for comparison), from diverse human cell lines (ENCODE). DNAse I HS is a marker for open chromatin that is commonly found at active cis-regulatory sequences including promoters and enhancers. At the GLI1 and PRKACA loci, note the multiple, relatively uniform density signal peaks (appearing as dark vertical lines) for the majority of cell lines particularly immediately adjacent to transcription start sites (TSSs), illustrating “open chromatin” that is consistent with expression of both genes in a wide range of tissues. In contrast, strong DNAse I HS signal at the ARHGAP36 locus is observed in the subset of the 75 cell lines of developmental origin marked with a purple vertical bar [Embryonic stem cells (H1hESC, H7hESC) and induced pluripotent stem (iPS CWRU1, iPS NIHi11, iPS NIHi7) cell lines]. The signal density for these is highest adjacent to several ARHGAP36 TSSs, corresponding to the multiple known transcripts, with weak signal levels observed for most of the other cell lines.

**Appendix 1—figure 4.**
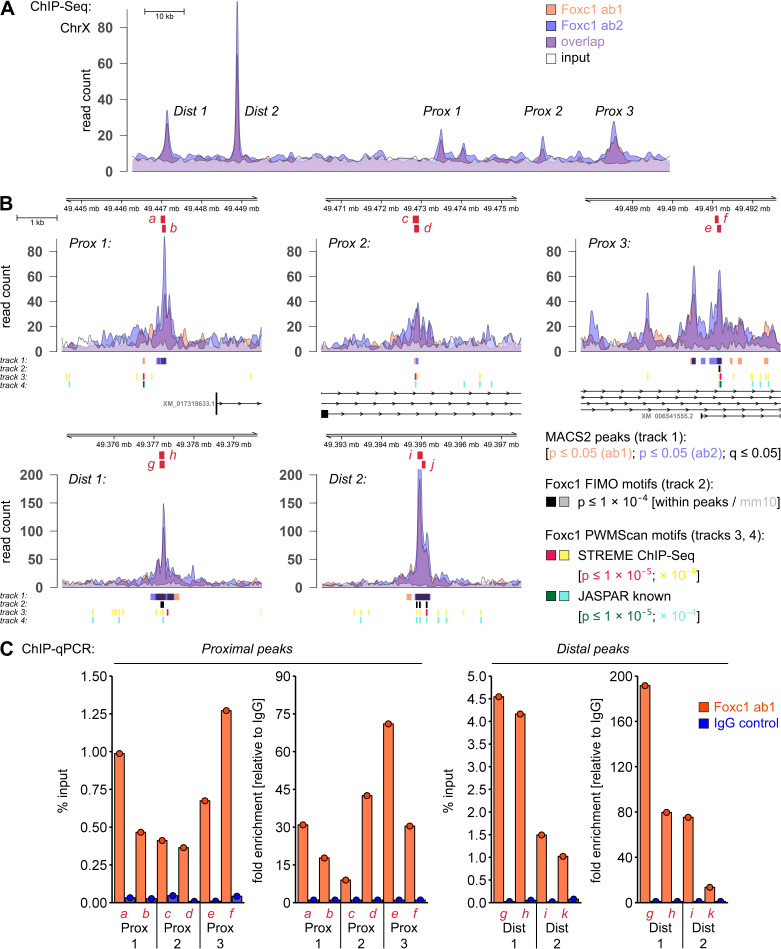
ChIP-qPCR validation of Foxc1 ChIP-Seq peaks in the vicinity of Arhgap36 gene. (**A, B**) Plots demonstrate read distribution for Foxc1 ChIP-Seq data from Figure 2 [(**A**) entire locus (within ± 100 kb of Arhgap36 ORFs); (**B**) individual peaks (5 kb regions); location of amplicons (a-j) that were analysed by ChIP-qPCR is indicated in red]. (**C**) ChIP-qPCR data­ confirm substantial enrichment of signal in all proximal and distal peaks identified by ChIP-Seq (N = 1).

**Appendix 1—figure 5.**
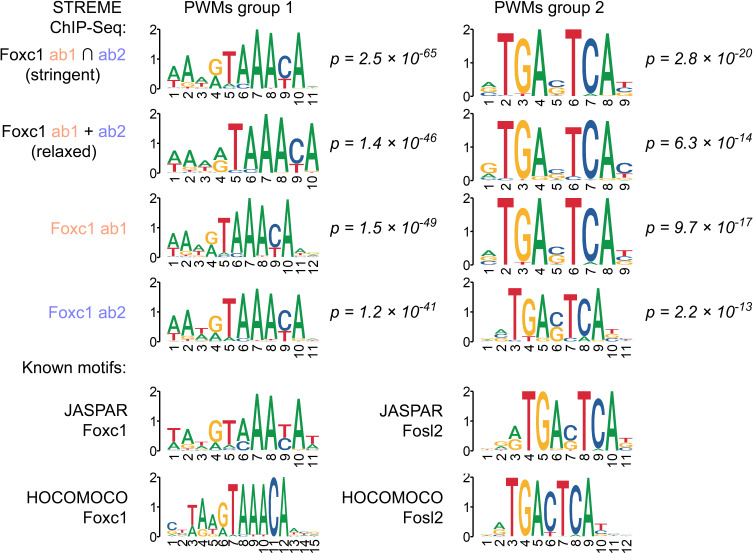
De novo motifs detected in Foxc1 ChIP-Seq peaks using STREME closely resemble known binding motifs of Foxc1 and Fosl2. De novo motif analysis conducted using STREME tool identified two major groups of significantly enriched motifs (PWMs groups 1 and 2). Group 1 PWMs (ranked top #1 for intersected peaks) share high degree of similarity with known Foxc1 motifs, while Group 2 PWMs (ranked top #2 for intersected peaks) are highly similar to motifs of Fos-Jun family TFs, most prominently Fosl2. Notably, when analysed separately, ChIP-Seq data obtained using each of two anti-Foxc1 antibodies produce highly similar de novo PWMs. Known motifs of Foxc1 and Fosl2 transcription factors were retrieved from JASPAR and HOCOMOCO databases for comparison.

**Appendix 1—figure 6.**
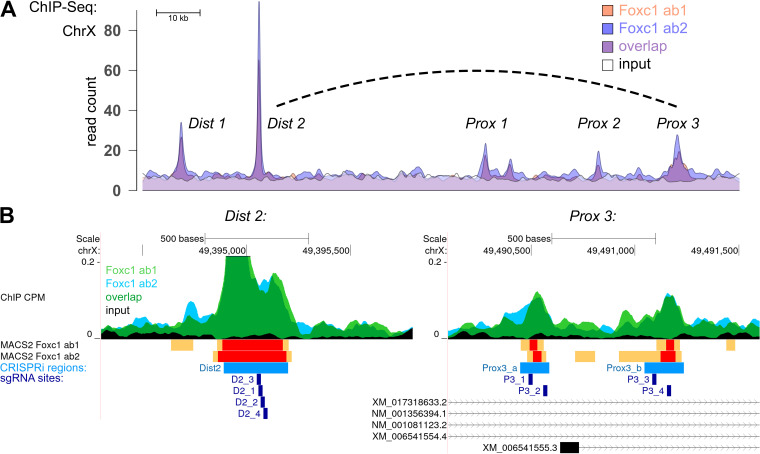
CRISPRi analysis of potential Foxc1-binding sites in the vicinity of Arhgap36 gene. (**A**) Plot shows read distribution for the ChIP samples and input chromatin control with five identified peaks (Prox-1 to Prox-3, Dist-1 and Dist-2). Dashed line demonstrates potential relationship between Prox-3 and Dist-2, as promoter and enhancer regions for murine Arhgap36. Panel (**B**) shows (1) ChIP-Seq coverage at both affected peaks; (2) nearby location of TSS for a major Arhgap36 isoform within the wide Prox-3 peak (approximately 80 – 110 bp downstream of narrow peak Prox-3a, and 500 bp upstream narrow peak Prox-3b); (3) location of individual sgRNA­ sites used for CRISPRi targeting.

**Appendix 1—figure 7.**
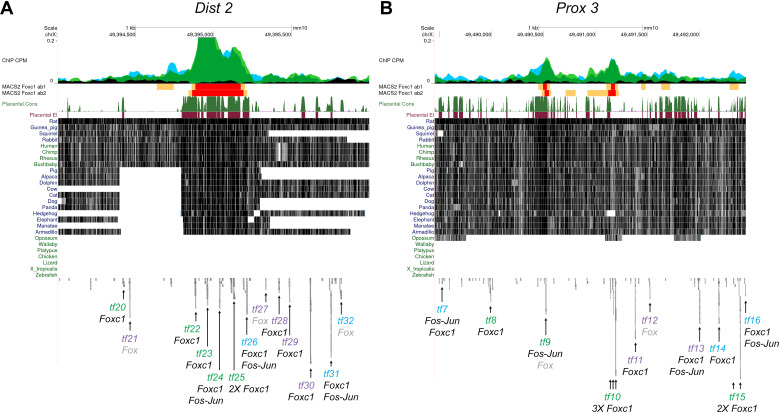
Conservation in Prox 3 and Dist 2 regions of Arhgap36 locus. (**A, B**) Representation of portions of the Arhagp36 locus corresponding to ChIP-seq peaks Prox-3 and Dist-2. Depicted in order are: ChIP-seq tracks with read coverage for two Foxc1 antibodies, UCSC-derived placental and non-placental mammal conservation data [Multiz alignment for representative vertebrate species, black lines], and distribution of predicted Fox and Fos-Jun transcriptio

**Appendix 1—figure 8.**
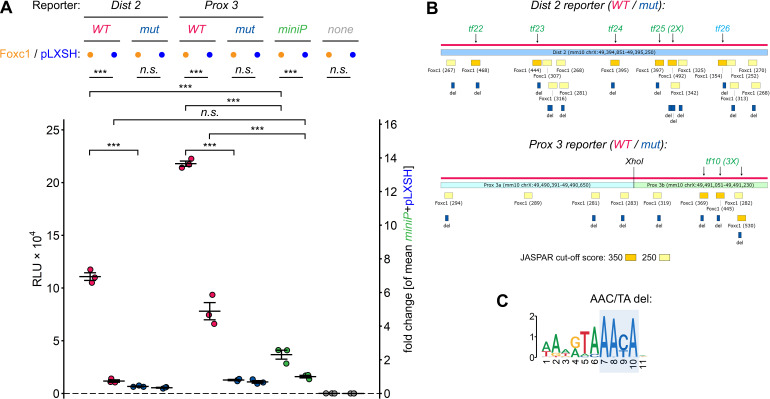
Two regions of the Arhgap36 locus sustain Foxc1-dependent transcription in vitro. (**A**) Luciferase reporter assays using wild-type or mutated Dist 2 and Prox 3 sequences upstream of a minimal promoter demonstrate that both wild-type reporters sustain significantly elevated transcriptional activity compared to the minimal reporter. Transcriptional activity is diminished by the absence of Foxc1 and abrogated by mutation of predicted Foxc1-binding motifs. (**B**) Depicts for each region locations of: (i) Foxc1-binding motifs predicted using stringent (orange) or relaxed (yellow) JASPAR criteria, (ii) the stringent Foxc1-binding motifs from Appendix 1—figure 7, and (iii) in blue, the core AAC/TA sequences in Foxc1’s binding motif (**C**) that were deleted in the mutated reporter constructs. [Wild-type (WT), mutated (mut), minimal promoter (miniP)]

**Appendix 1—figure 9.**
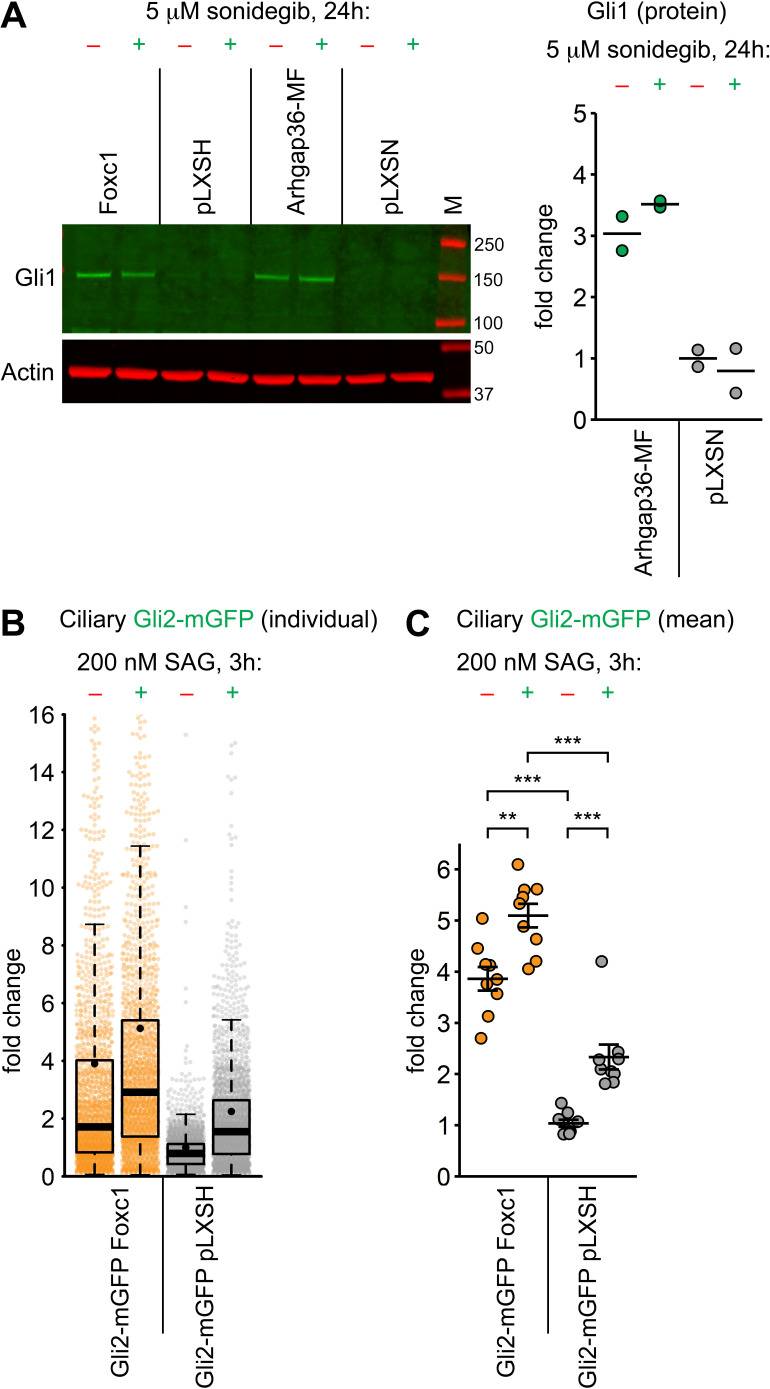
Foxc1 expression phenocopies Arhgap36-induced resistance to Smoothened inhibition and facilitates ciliary accumulation of Gli2. (**A**) Quantitative western blotting demonstrates that resistance to sonidegib inhibition observed in Foxc1-expressing Gli2-mGFP NIH3T3 cells recapitulates observations in NIH3T3 cells with ectopic expression of Myc-FLAG Arhgap36 [Arhgap36-MF]. (**B, C**) Accumulation of Gli2-mGFP at the tips of primary cilia in Gli2-mGFP NIH3T3 cells that express Foxc1, relative to relevant empty vector control [pLXSH]. Cells serum-starved for 2 Appendix 1—figure 9—source data 1.PDF file containing original western blots for Appendix 1—figure 9, indicating the relevant bands and treatments. Appendix 1—figure 9—source data 2.Original files for western blot analyses displayed in Appendix 1—figure 9.

**Appendix 1—figure 10.**
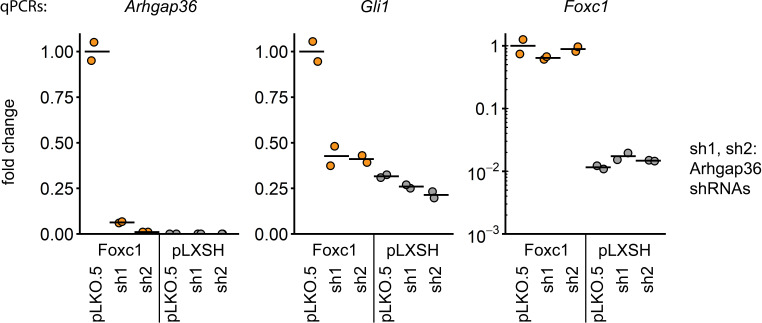
Foxc1-induced Gli1 expression is attributable to Arhgap36. qPCR analyses demonstrate substantial increase in Gli1 mRNA levels in NIH3T3 cells expressing Foxc1. This effect is reversed by shRNA-mediated knock-down of Arhgap36 expression.

**Appendix 1—figure 11.**
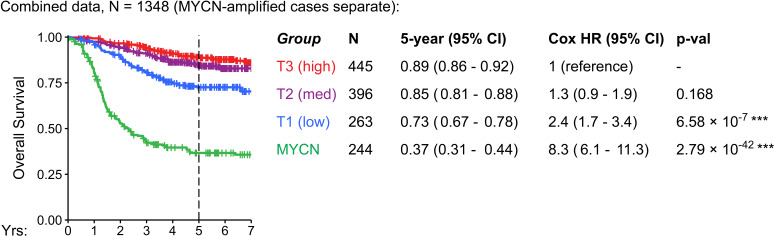
Overall survival of neuroblastoma patients stratified by Arhgap36 expression levels and MYCN amplification status. Analysis was performed on the GSE49711, E-MTAB-1781 and TARGET 2018 data merged into a single dataset comprising n=1348 individuals (Figure 8D). MYCN-amplified cases were segregated into a separate group. Kaplan-Meier plots illustrate three features: (i) best overall survival for patients with high Arhgap36 expression and without MYCN amplification, (ii) preserved lower overall survival rate among patients with low Arhgap36 expression and without MYCN amplification (HR 1.7 – 3.4), and (iii) predictably poor survival among high-risk MYCN-amplified cases (HR 6.1 – 11.3 compared to Arhgap36 high reference).

**Appendix 1—figure 12.**
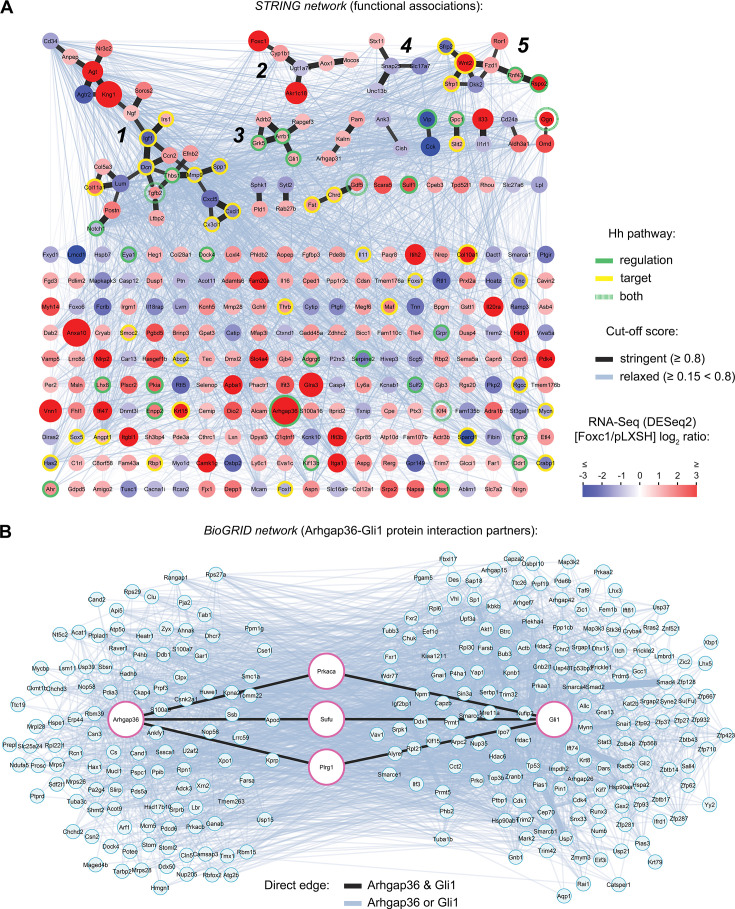
Network analysis of Foxc1-driven differential gene expression patterns. (**A**) Five highly functionally-associated clusters of 4 or more genes were identified using the STRING database [black lines; cutoff score ≥ 0.8]. Notably, cluster #3 contains Hh components Gli1, ­Grk5 and Arrb1. The individual node colour reflects differential gene expression (Figure 1A); genes regulating or targeted by Hh signalling, depicted in green / yellow circles. (**B**) Protein interaction partners of Arhgap36 and Gli1 retrieved from BioGRID database reveals multiple interactions between those binding Gli1 or Arhgap36, however PKAC, Sufu and Plrg1 are the only proteins to directly interact with both.

## Data Availability

The RNA-sequencing and ChIP-sequencing data have been deposited to the NCBI GEO database with the following accession numbers: GSE297719 (RNA-seq), GSE297865 (ChIP-seq). Additional experimental data have been deposited at the Bio Image Archive (accession number S-BSST3066). The following datasets were generated: HavrylovS LehmannO 2025Gene expression changes associted with overexpression of Foxc1 transcription factor in NIH3T3 fibroblastsNCBI Gene Expression OmnibusGSE297719 HavrylovS LehmannO 2025Foxc1 ChIP-seq in Gli2-mGFP NIH3T3 cells stably expressing Foxc1 ORFNCBI Gene Expression OmnibusGSE297865 HavrylovS 2026Mechanistic insights into transcriptional regulation of ARHGAP36 expression identify a factor predictive of neuroblastoma survivalBioStudiesS-BSST306610.7554/eLife.108827PMC1333677742405753

## Additional files

Supplementary file 1. Four datasets providing additional information. Table A. List of differentially expressed genes in NIH3T3 cells expressing Foxc1 or pLXSH empty vector control. Complete list of differentially expressed genes identified in NIH3T3 cells expressing Foxc1 or empty vector control [pLXSH] by RNA-sequencing (*n* = 3) and used for volcano plot (Figure 1A). *n* = 292 differentially expressed genes; 195 upregulated, 97 downregulated. Selection criteria: *q*-value (FDR-adjusted p-value) ≤0.01, absolute log_2_ ratio ≥1, signal cut-off ≥1 FPKM in either condition. Table B. Functional profiling of the list of differentially expressed genes from File S1. Functional profiling of the list of genes differentially expressed in Foxc1-expressing NIH3T3 cells vs empty vector control [pLXSH] was performed using gene set enrichment analysis (GSEA) platform Enrichr [https://maayanlab.cloud/Enrichr/]. The list was analyzed against multiple gene set libraries and databases. Results demonstrate significant associations between differentially expressed genes and molecular pathways/biological processes/pathological conditions with previous implication of Foxc1, as well as Foxc1-specific cell and tissue expression. Overall, these results indicate that ectopic expression of Foxc1 in NIH3T3 affects functionally relevant targets. Examples include association with ocular and vascular disease [glaucoma p = 5.8 × 10^–4^; intraocular pressure p = 5.9 × 10^–3^; hypertension p = 5.7 × 10^–4^; myocardial ischemia p = 2.4 × 10^–4^; coronary artery disease p = 0.01], vascular development [regulation of angiogenesis p = 9.4 × 10^–5^], bone development [endochondral ossification 2.8 × 10^–4^], a number of mouse skeletal and renal phenotypes [MGI phenotypes]; multiple cancers and cancer invasiveness phenotypes [epithelial mesenchymal transition p = 7.7 × 10^–11^]. Relevant cell and tissue expression signatures [mesenchyme p = 3.4 × 10^–8^; mesenchymal stem cells p = 5.8 × 10^–4^; (mesenchymal) stromal cells p = 1.8 × 10^–6^; vasculature p = 2.7 × 10^–5^; neural crest p = 3.8 × 10^–4^; several skeletal tissues; differentiating osteoblasts p ≤ 1.4 × 10^–6^]. p-values are FDR-adjusted. Table C. List of predicted Fox and Fos–Jun transcription factor-binding sites in Proximal 3 and Distal 2 regions of the murine Arhgap36 locus. Table D. Data derived from online resources for expression of ARHGAP36 in multiple tumor types.

MDAR checklist

## Appendix 1

### Supplemental methods

#### ChIP-qPCR analyses

Preparation of enriched chromatin samples for ChIP-PCR analyses was performed the same way, except immunoprecipitation was performed with anti-Foxc1 antibody alongside non-specific normal IgG control. Resulting samples were analyzed by qPCR using pairs of primers designed to individual peak regions identified by ChIP-sequencing.

#### Detailed ChIP protocol

Cross-linked chromatin was prepared and immunoprecipitated from Gli2-mGFP NIH3T3 reporter cells stably expressing *Foxc1* ORF using Simple ChIP plus Enzymatic Chromatin IP Kit (New England Biolabs). Unless indicated otherwise, formulation of all reagents used was according to the manufacturer’s instructions. In detail, 3 million cells were seeded per 15 cm dish and grown for 72 hr in DMEM media supplemented with 10% FBS, 80 µg/ml Hygromycin, and 500 µg/ml Geneticin. 24 hr prior to cross-linking, media were replaced with 30 ml fresh DMEM supplemented with 10% FBS. To crosslink proteins to DNA, 1873 µl of 16% paraformaldehyde (PFA) solution was added to each plate. After 10 min incubation at room temperature, 3 ml of 10X glycine solution was added to each plate and incubated for an additional 5 min at room temperature to quench the remaining PFA. Following two washes with 20 ml ice-cold PBS per plate, cells were scraped in 2 ml ice-cold PBS solution supplemented with 10 µl of 200X Protease Inhibitor Cocktail (PIC) and centrifuged at 2000 × *g* for 5 min, at 4°C. For preparation of nuclei and chromatin digestion, the cell pellets were re-suspended in 2 ml of 1X buffer A supplemented with 1 µl 1 M DTT and 10 µl of 200X PIC and incubated for 10 min on ice, with mixing every 3 min. Nuclei were next pelleted by centrifugation at 2000 × *g* for 5 min, at 4°C. Resulting nuclear pellet was resuspended in 500 µl of 1X buffer B with 0.25 µl of 1 M DTT, and 2.5 µl of Micrococcal Nuclease (MNase) was added to each tube to fragment DNA, and samples were incubated for 20 min at 37°C with frequent mixing [optimization of MNase quantities was performed beforehand according to the manufacturer’s protocol for optimization of chromatin digestion, to achieve DNA fragmentation range of 200–800 bp]. Digestion was stopped by addition of 50 µl of 0.5 M EDTA to each sample following 2 min incubation on ice. Next, nuclei were pelleted by centrifugation at 16,000 × *g* for 1 min, at 4°C, and re-suspended in 325 µl of ChIP buffer per sample. A total of 650 µl sample lysates were combined, kept for 10 min on ice, and further sonicated with 4 sets of 10-s pulses, separated by 20-s breaks, using Model 60 Sonic Dismembrator (Fisher Scientific), set at ~10 W power output. Lysates were clarified by centrifugation at 9400 × *g* for 10 min, at 4°C, and chromatin-containing supernatants were transferred to new tubes. After analysis of digestion, chromatin concentration was normalized to 150 µg/ml, in ChIP buffer with PIC. Chromatin immunoprecipitation was performed by mixing 100 µl chromatin sample, 400 µl of 1X ChIP buffer with PIC and antibodies (Foxc1 CST – 3 µl/sample; Foxc1 Abcam – 2 µl [2 µg], Normal IgG – 2 µl/sample [2 µg], H3 positive control – 10 µl), and incubation overnight at 4°C with rotation. Next day, 30 µl of ChIP-grade Protein G Magnetic Beads were added to each tube, and samples were incubated for an additional 2 hr at 4°C with rotation. Beads were pelleted using a magnetic separation rack, washed three times for 5 min at 4°C in 1 ml of 1X ChIP buffer (low salt wash), and then one time for 5 min at 4°C in 1 ml of high salt wash (1X ChIP buffer with extra 350 mM NaCl). Following the washes, chromatin was eluted in 150 µl of 1X ChIP Elution buffer per sample for 30 min at 65°C, with occasional gentle vortexing. After pelleting the beads, resulting immunoprecipitated supernatant containing eluted chromatin was transferred into new tubes. Reversal of cross-links in immunoprecipitated chromatin, as well as equal volumes of 2% chromatin input samples, was done by addition of 6 µl of 5 M NaCl and 2 µl Proteinase K, followed by 2 hr digestion at 65°C and DNA purification using spin columns according to kit manufacturer’s instructions.

#### List of plasmids

**Table:**

Name	Notes	Source
Arhgap36-MF-pLXSN	Retroviral vector for expression of Myc-FLAG-tagged mouse Arhgap36. In-Fusion HD cloning from mouse Arhgap36-Myc-DDK ORF clone (Origene MR222487); pLXSN linearized by EcoRI/BamHI digestion; PCR cloning primers: F: TTCTCTAGGCGCCGGAATTCATGGCGTGGATGCTGGACTG R: ACATTCCACAGCCGGATCCTTAAACCTTATCGTCGTCATCC	This study
Foxc1-pLXSH (mouse)	Retroviral vector for expression of mouse Foxc1. Previously generated in our laboratory.	PMID:39217245
FOXC1-pLXSH (human)	Retroviral vector for expression of human FOXC1. In-Fusion HD cloning from human pENTR221-FOXC1 WT ORF (generous gift from Dr. Mike Walter, University of Alberta, Edmonton, AB); pLXSH linearized by BamHI digestion; PCR cloning pairs: F: TCGTTAACTCGAGGATCCATGCAGGCGCGCTACTCCGTG R: CTTGTCGACAGATCTGGATCCTCAAAACTTGCTACAGTCGTAGACG	This study
pLXSN, pLXSH, vPak-VGV,	Empty pLXSN and pLXSH vector backbones, vPak-VGV helper plasmid (and 293-VSV-G packaging cell line) were gifts from Dr. Morag Park (McGill University, Montreal, QC).	McGill University
pLX303-ZIM3-KRAB-dCas9	Lentiviral vector for mammalian expression of ZIM3 KRAB domain fused to the N terminus of dCas9. Used in CRISPRi.	Addgene #154472
pLCKO, pLCKO-LacZ-sgRNA, pLCKO-Luc-sgRNA	Empty pLCKO lentiviral backbone and pLCKO vectors for expression of non-targeting LacZ and Luciferase sgRNAs under U6 promoter. Used for CRISPRi. pLCKO, pLCKO-LacZ-sgRNA and pLCKO-Luciferase-sgRNA vectors were gifts from Dr. Jason Moffat (University of Toronto, Toronto, ON).	Addgene #73311
pLCKO-Arhgap36-sgRNA vectors	Lentiviral pLCKO vectors for expression sgRNA pools targeting various regions in vicinity of Arhgap36 locus: Dist 1 (D1 vector pool), Dist 2 (D2 vector pool), Prox l (P1 vector pool), Prox 2 (P2 vector pool), Prox 3 (P3 vector pool). Used for CRISPRi. In-Fusion HD cloning of annealed primer duplexes; pLCKO vector was linearized by BfuAI digestion. Primer duplex pairs (F/R): D1_1F: aaggacgaggtaccg ACATACCCTAGGATGCAATA gttttagagctagaa D1_1R: ttctagctctaaaac TATTGCATCCTAGGGTATGT cggtacctcgtcctt D1_2F: aaggacgaggtaccg GTGGTACAGCAAGTAAATAA gttttagagctagaa D1_2R: ttctagctctaaaac TTATTTACTTGCTGTACCAC cggtacctcgtcctt D1_3F: aaggacgaggtaccg AGCGCCCTTATTGCATCCTA gttttagagctagaa D1_3R: ttctagctctaaaac TAGGATGCAATAAGGGCGCT cggtacctcgtcctt D1_4F: aaggacgaggtaccg GTACCACTCTATCCACAAGG gttttagagctagaa D1_4R: ttctagctctaaaac CCTTGTGGATAGAGTGGTAC cggtacctcgtcctt D2_1F: aaggacgaggtaccg TTTCTCTAGTGGTATTCGGA gttttagagctagaa D2_1R: ttctagctctaaaac TCCGAATACCACTAGAGAAA cggtacctcgtcctt D2_2F: aaggacgaggtaccg GTATTCGGATGGTTGTATTT gttttagagctagaa D2_2R: ttctagctctaaaac AAATACAACCATCCGAATAC cggtacctcgtcctt D2_3F: aaggacgaggtaccg CCACTAGAGAAAATATTGTT gttttagagctagaa D2_3R: ttctagctctaaaac AACAATATTTTCTCTAGTGG cggtacctcgtcctt D2_4F: aaggacgaggtaccg TGTATTTGGGGGACTTGATT gttttagagctagaa D2_4R: ttctagctctaaaac AATCAAGTCCCCCAAATACA cggtacctcgtcctt P1_1F: aaggacgaggtaccg AGCCAAGAGAGTGTGTGTTT gttttagagctagaa P1_1R: ttctagctctaaaac AAACACACACTCTCTTGGCT cggtacctcgtcctt P1_2F: aaggacgaggtaccg GATACTCCAGAACTGTGCTT gttttagagctagaa P1_2R: ttctagctctaaaac AAGCACAGTTCTGGAGTATC cggtacctcgtcctt P1_3F: aaggacgaggtaccg TTGACATGCTCCTAATTTAA gttttagagctagaa P1_3R: ttctagctctaaaac TTAAATTAGGAGCATGTCAA cggtacctcgtcctt P2_1F: aaggacgaggtaccg CAACTGTGTCGAATTTGCAT gttttagagctagaa P2_1R: ttctagctctaaaac ATGCAAATTCGACACAGTTG cggtacctcgtcctt P2_2F: aaggacgaggtaccg ACACTGACTAAACAGAAAAT gttttagagctagaa P2_2R: ttctagctctaaaac ATTTTCTGTTTAGTCAGTGT cggtacctcgtcctt P2_3F: aaggacgaggtaccg TCGAATTTGCATTGGTGTGT gttttagagctagaa P2_3R: ttctagctctaaaac ACACACCAATGCAAATTCGA cggtacctcgtcctt P3_1F: aaggacgaggtaccg ACAATTCCGAGGGAAATCAA gttttagagctagaa P3_1R: ttctagctctaaaac TTGATTTCCCTCGGAATTGT cggtacctcgtcctt P3_2F: aaggacgaggtaccg TCATCAGCCCAAAAAGGAGA gttttagagctagaa P3_2R: ttctagctctaaaac TCTCCTTTTTGGGCTGATGA cggtacctcgtcctt P3_3F: aaggacgaggtaccg TAGATATAAGAAATGCTTGT gttttagagctagaa P3_3R: ttctagctctaaaac ACAAGCATTTCTTATATCTA cggtacctcgtcctt P3_4F: aaggacgaggtaccg AAAAAGAAAATATACTTCTT gttttagagctagaa P3_4R: ttctagctctaaaac AAGAAGTATATTTTCTTTTT cggtacctcgtcctt	This study
psPAX2, pMD2.G	Retroviral packaging plasmids used to package pLCKO, pLKO.1, pLKO.5 vectors. These plasmids were obtained through RNAi Screening Core, Li Ka Shing Institute of Virology, University of Alberta, Edmonton, AB.	University of Alberta
pLKO.1	Empty shRNA vector control. Obtained through RNAi Screening Core, Li Ka Shing Institute of Virology, University of Alberta, Edmonton, AB.	University of Alberta
pLKO.5	Empty shRNA vector control. Cat. #SHC201.	Millipore-Sigma
Arhgap36 sh1 (mouse)	Mouse Arhgap36 shRNA targeting vector (pLKO.5 backbone). TRC clone ID: TRCN 0000 268750	Millipore-Sigma
Arhgap36 sh2 (mouse)	Mouse Arhgap36 shRNA targeting vector (pLKO.5 backbone). TRC clone ID: TRCN 0000 268695	Millipore-Sigma
Arhgap36 sh1 (human)	Human Arhgap36 shRNA targeting vector (pLKO.5 backbone). TRC clone ID: TRCN 0000 421375	Millipore-Sigma
Arhgap36 sh2 (human)	Human Arhgap36 shRNA targeting vector (pLKO.5 backbone). TRC clone ID: TRCN 0000 421905	Millipore-Sigma
Arhgap36 sh3 (human)	Human Arhgap36 shRNA targeting vector (pLKO.5 backbone). TRC clone ID: TRCN 0000 416368	Millipore-Sigma
Arhgap36 sh4 (human)	Human Arhgap36 shRNA targeting vector (pLKO.1 backbone). TRC clone ID: TRCN 0000 147647	Millipore-Sigma
Arhgap36 sh5 (human)	Human Arhgap36 shRNA targeting vector (pLKO.1 backbone). TRC clone ID: TRCN 0000 148948	Millipore-Sigma

#### List of antibodies

**Table:**

Target	Cat. #	Description	Source	Figure
Arhgap36/ARHGAP36	HPA002064	Anti-ARHGAP36 rabbit polyclonal antibody; WB dilution 1:1000–1:5000 (recommended 1:5000; non-specific binding in western blots can be reduced by addition of 0.25% Triton X-100 to TBST); IF dilution 1:500–1:1200.	Millipore-Sigma, Atlas Antibodies	1, 4, 5, S4, S9, S11
β-Actin	# 3700	β-Actin (8H10D10) mouse monoclonal antibody; WB dilution 1:10,000–1:20,000.	Cell Signaling	1, 4–6, S4, S7, S9, S11
Gli1/GLI1	# 2534	Gli1(V812) rabbit polyclonal antibody; WB dilution 1:500–1:1000.	Cell Signaling	1, 5, S7
Foxc1/FOXC1	# 8758	Foxc1 (D8A6) rabbit monoclonal antibody; WB dilution 1:500–1:1000; IF dilution 1:250–1:500.	Cell Signaling	1, 2, S3–S5, S9
Foxc1	Ab227977	Foxc1 (EPR20685) rabbit monoclonal antibody.	Abcam	2, S3, S5
IgG control	# 2729	Normal rabbit IgG	Cell Signaling	S3
PKAC	610980	Purified Mouse Anti-PKA[C]; WB dilution 1:2000; IF dilution: 1:500–1:2000.	BD Trans-duction	3, S4, S9, S11
pT197 PKAC	# 5661	Phospho-PKA C (Thr197) (D45D3) rabbit mAb; WB dilution 1:2000; IF dilution: 1:250.	Cell Signaling	3, S4, S9
α-tubulin	# 3873	α-Tubulin (DM1A) mouse mAb; WB dilution 1:10,000–1:20,000.	Cell Signaling	3, 4, S4, S9, S11
dCas9	# 14697	Cas9 (*S. pyogenes*) (7A9-3A3) mouse mAb. WB dilution 1:1000.	Cell Signaling	4
Sufu	# 2520	SUFU (C54G2) rabbit mAb; WB dilution 1:1000; IF dilution 1:250.	Cell Signaling	6
pS342 Sufu	# 11552	Sufu (Phospho-Ser342) rabbit polyclonal antibody. WB dilution 1:1000.	SAB Signalway	6
Arl13b	Ab136648	Anti-ARL13B [N295B/66] mouse monoclonal antibody. IF dilution 1:250.	Abcam, Neuromab	6
Arl13b/ARL13b	17711-1-AP	Arl13b rabbit polyclonal antibody; IF dilution 1:500; same antibody conjugated with CF555 dye using Mix-n-Stain antibody labeling kit (Biotium, Cat. # 92254) can be used at IF dilution 1:200.	Proteintech	7
γ-Tubulin	T5326	γ-Tubulin mouse monoclonal antibody, clone GTU-88; IF dilution 1:500.	Millipore-Sigma	7
AF555 donkey anti-rabbit	A-31572	Donkey anti-rabbit highly cross-adsorbed secondary antibody, Alexa Fluor 555; IF dilution 1:500–1:1000.	Thermo Fisher	1, 3, 6, 9, S4
AF647 goat anti-mouse	A-21237	F(ab′)2-Goat anti-mouse cross-adsorbed secondary antibody, Alexa Fluor 647; IF dilution 1:500–1:1000.	Thermo Fisher	3, 6, S4
800CW goat anti-rabbit	926-32211	IRDye 800CW goat anti-rabbit secondary antibody; WB dilution 1:15000	Li-Cor	1, 3–6, S4, S7, S9, S11
680RD donkey anti-mouse	925-68072	IRDye 680RD donkey anti-mouse secondary antibody; WB dilution 1:15000	Li-Cor	1, 3–6, S4, S7, S9, S11

#### List of ChIP-PCR primers

**Table:**

Target	Pair	Sequence	Figure
*Prox 1 (a)*	F R	CCAAAGCACAGTTCTGGAGTA GAGCCAAGAGAGTGTGTGTT	S3
*Prox 1 (b)*	F R	CCATTATTACAAGGCAATCGCTAAA AAATGTTACCAGGCCAGAGC	S3
*Prox 2 (c)*	F R	CGACACAGTTGACAGATATCCA GGAAATGCAGACTTTGTGTTTGT	S3
*Prox 2 (d)*	F R	TTCAGGGAAAGCTACTTAAA GCTGGAAATGCAGACTT	S3
*Prox 3 (e)*	F R	AAGGTATGTGAGGAGAAGAGAA GTAAACAAATGAGCAATGCTGT	S3
*Prox 3 (f)*	F R	CTCATGCTGTGCCTCAGTTA TTCTCTTCTCCTCACATACCTTAAT	S3
*Dist 1 (g)*	F R	GCACAGAAATGTGGAAGGAATC CCTTGTGGATAGAGTGGTACAG	S3
*Dist 1 (h)*	F R	GAGCTTGCAGCACAGAAATG GGATAGAGTGGTACAGCAAGTAAA	S3
*Dist 2 (i)*	F R	CATTCTGCTTCCTACTGTTCAAA GCTGATTCATCTCACTAATCATCAC	S3
*Dist 2 (j)*	F R	AGCTGTGATGATTAGTGAGATGA CCAAATACAACCATCCGAATACC	S3

All primers were from IDT.

#### List of qPCR primers

**Table:**

Target	Pair	Sequence	Figure
*Arhgap36* (mouse)	F R	GAT CCA GAG TGC TCG CAT AAA TTC AAG ACC TCG TGC ACA TC	1, 4, 7, S1
*Gli1* (mouse)	F R	GACTTTCTGGTCTGCCCTTT AGGAGGAAAGAGAGATCCTTCA	4, 5, 7, S1
*Foxc1* (mouse)	F R	CTCAACGAGTGCTTCGTCAA CTTCACTGCGTCCTTCTTCTT	7, S1
*ARHGAP36* (*human*)	F1 R1	GATCCAGAGTGCACGCATAA CTTCATCAGAAGGCTTGCTTTG	S9
*ARHGAP36* (*human*)	F2 R2	ATTCCTCCTGACAGCAACTTTA CCTCGCAGTTCTCAGTGATT	S9 (SK-N-FI)

All primers were from IDT.

#### Other reagents

**Table:**

Reagent	Cat. #	Description	Source	Figure
Sonidegib	S2151	Specific antagonist of *Smoothened* (Smo)	Selleckchem	5, S7
Cyclopamine	S1146	Specific antagonist of *Smoothened* (Smo)	Selleckchem	5
SAG	S7779	Specific agonist of *Smoothened* (Smo)	Selleckchem	5, 6, S7
Cycloheximide	S7418	Eukaryote protein synthesis inhibitor	Selleckchem	S5
Doxycycline hyclate	D5207	Tetracycline group antibiotic, used for Tet-inducible shRNA expression	Millipore-Sigma	S11
