# Supplementary figures and images for: Mechanistic insights into transcriptional regulation of ARHGAP36 expression identify a factor predictive of neuroblastoma survival
Source: eLife. 2026 Jul 6;14:RP108827. doi: 10.7554/eLife.108827 (PMC13336777; doi:10.7554/eLife.108827)

**Figure 1C:**

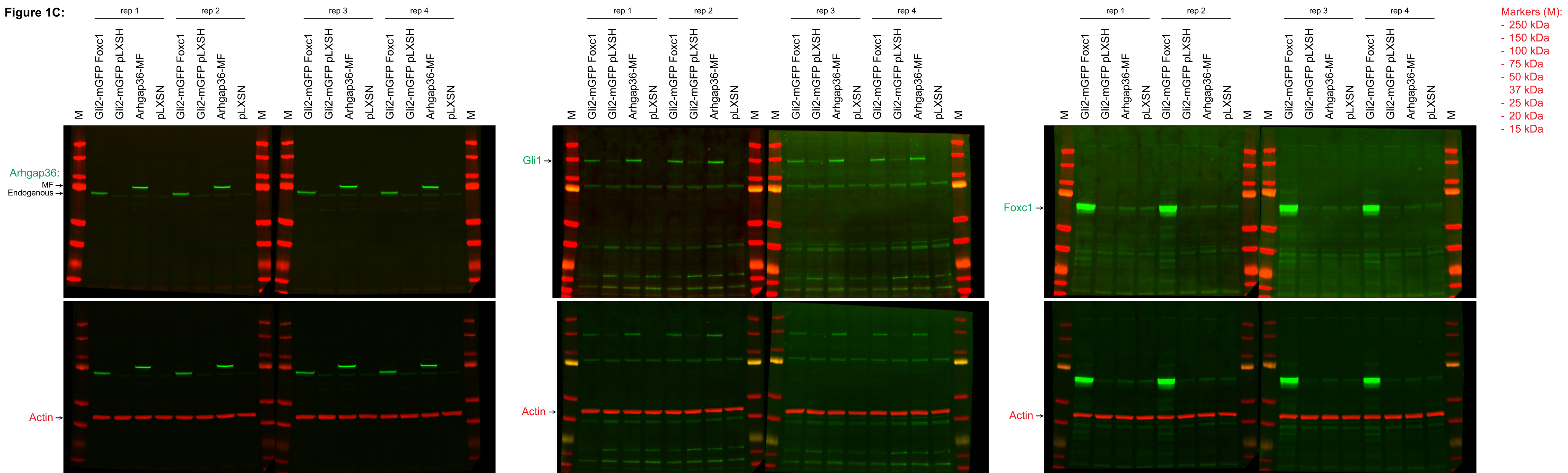

Supplement: Figure 1—source data 1. [file elife-108827-fig1-data1.zip › Figure 1-source data 1.pdf]

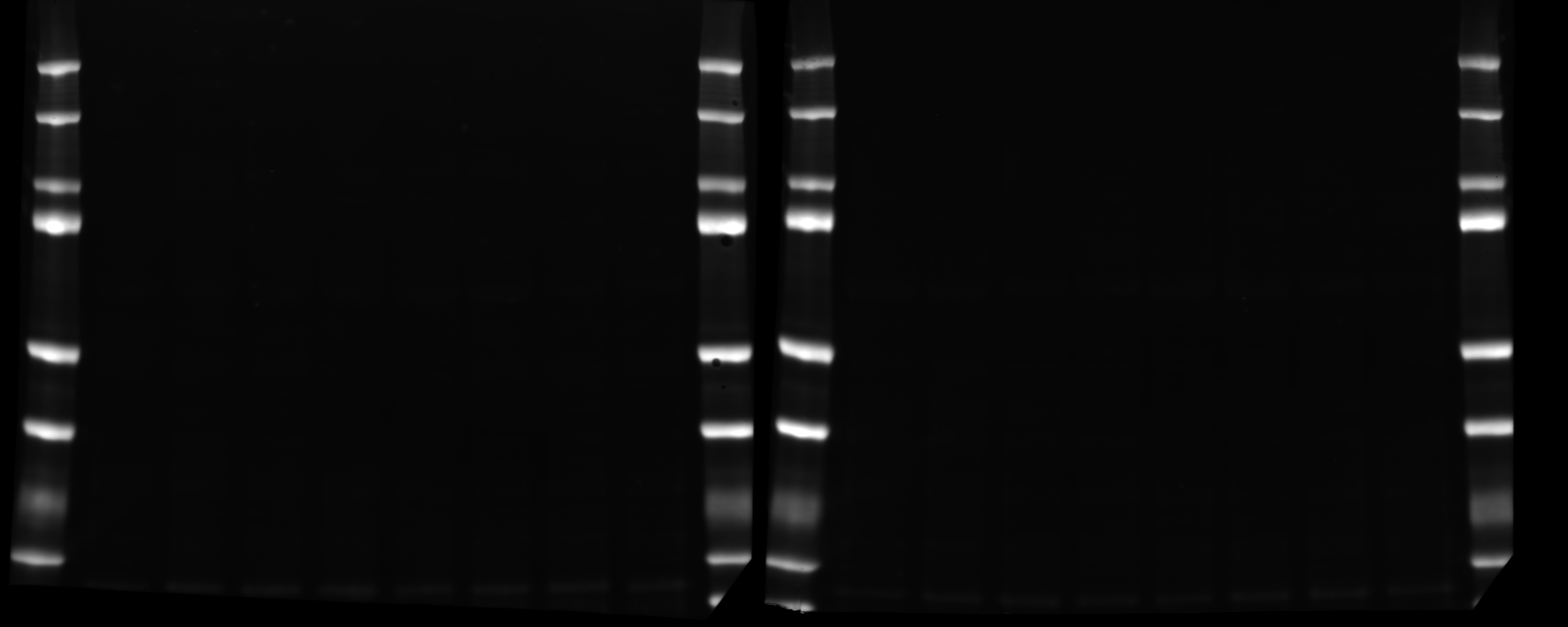

Supplement: Figure 1—source data 2. [file elife-108827-fig1-data2.zip › Figure 1C Arhgap36 16 bit 700.TIF]

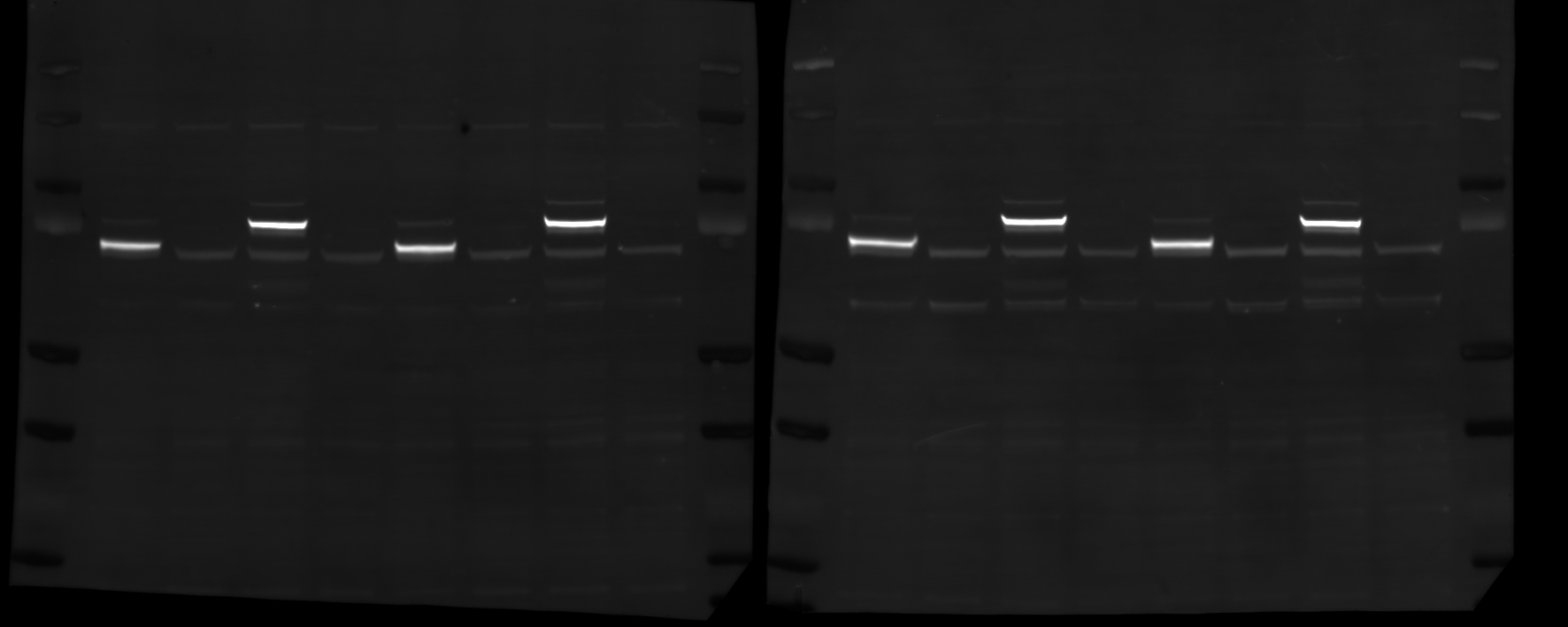

Supplement: Figure 1—source data 2. [file elife-108827-fig1-data2.zip › Figure 1C Arhgap36 16 bit 800.TIF]

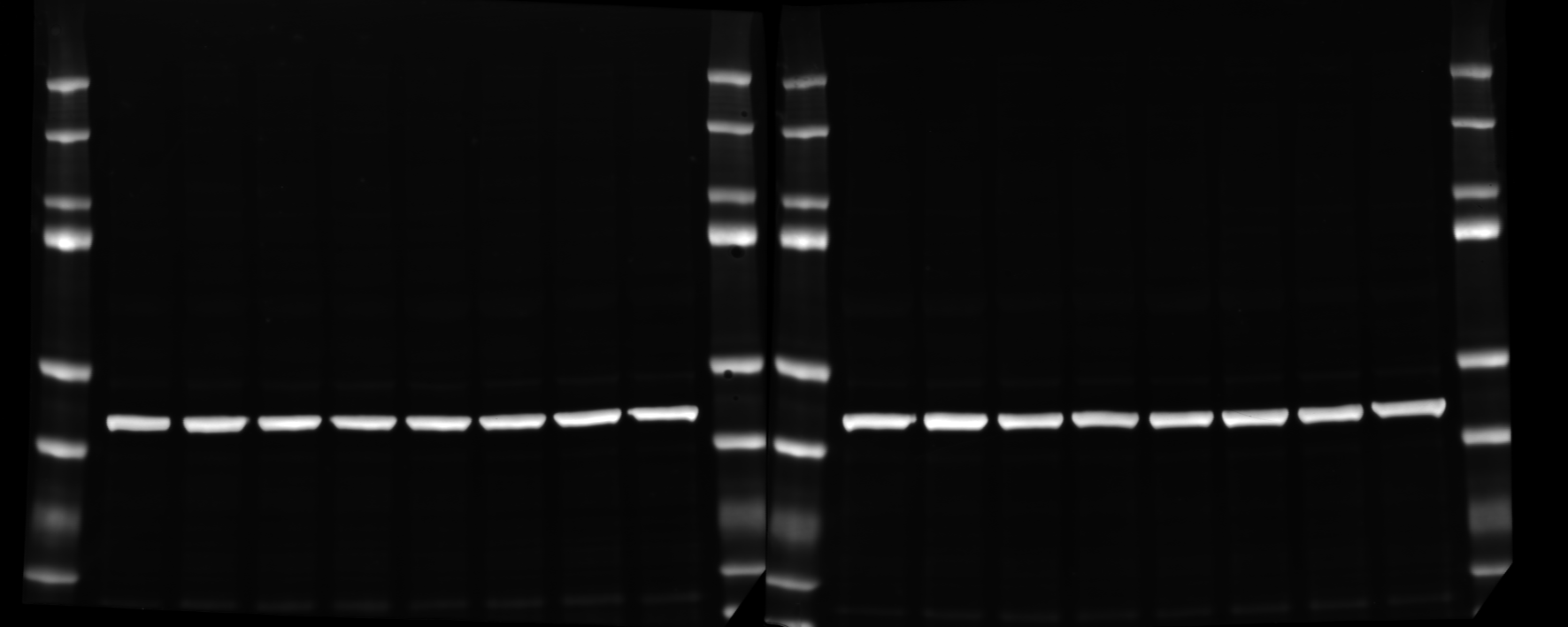

Supplement: Figure 1—source data 2. [file elife-108827-fig1-data2.zip › Figure 1C Arhgap36 reblot Actin 16 bit 700.TIF]

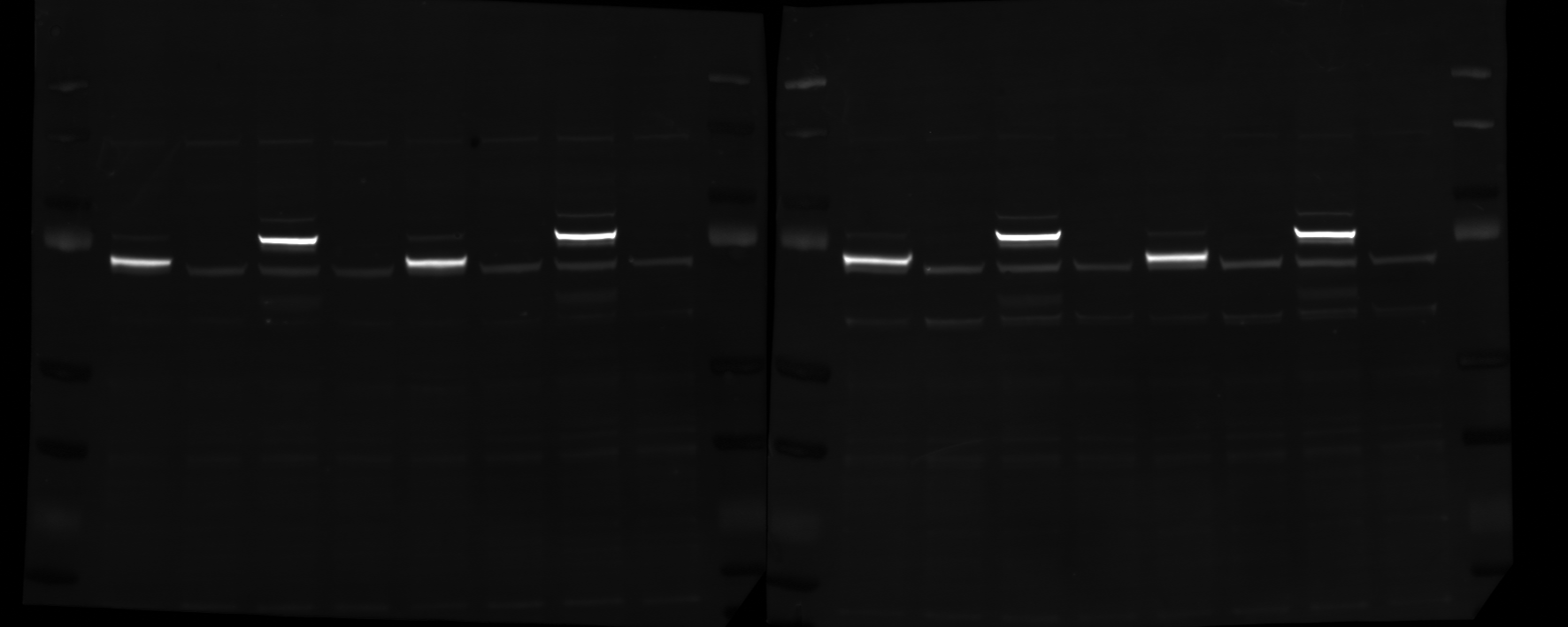

Supplement: Figure 1—source data 2. [file elife-108827-fig1-data2.zip › Figure 1C Arhgap36 reblot Actin 16 bit 800.TIF]

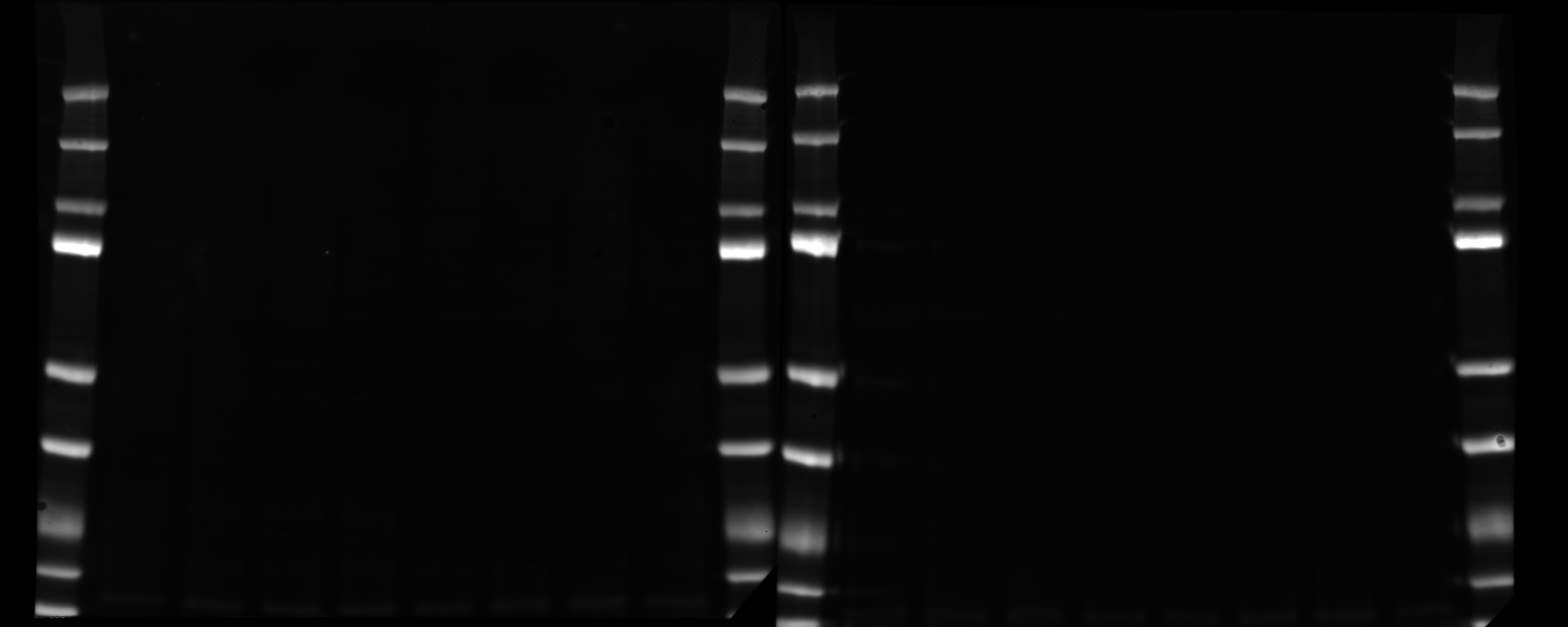

Supplement: Figure 1—source data 2. [file elife-108827-fig1-data2.zip › Figure 1C Foxc1 16 bit 700.TIF]

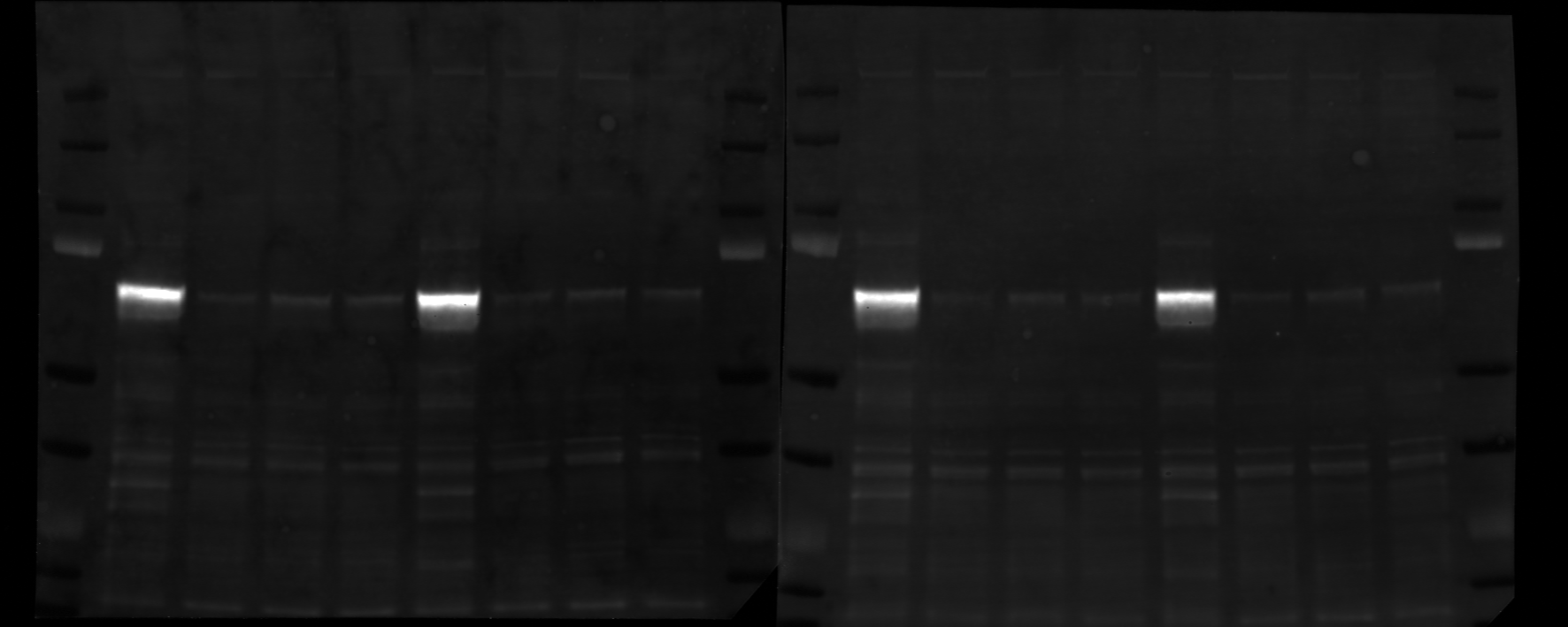

Supplement: Figure 1—source data 2. [file elife-108827-fig1-data2.zip › Figure 1C Foxc1 16 bit 800.TIF]

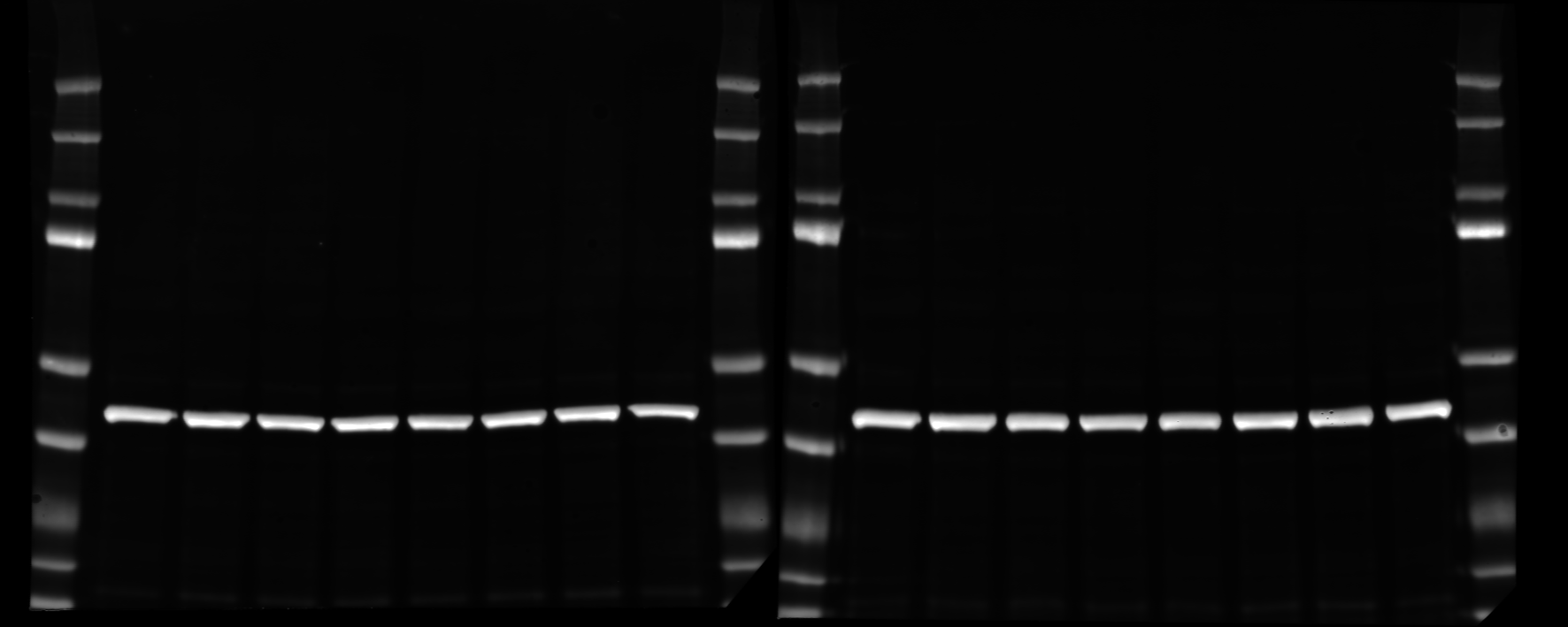

Supplement: Figure 1—source data 2. [file elife-108827-fig1-data2.zip › Figure 1C Foxc1 reblot Actin 16 bit 700.TIF]

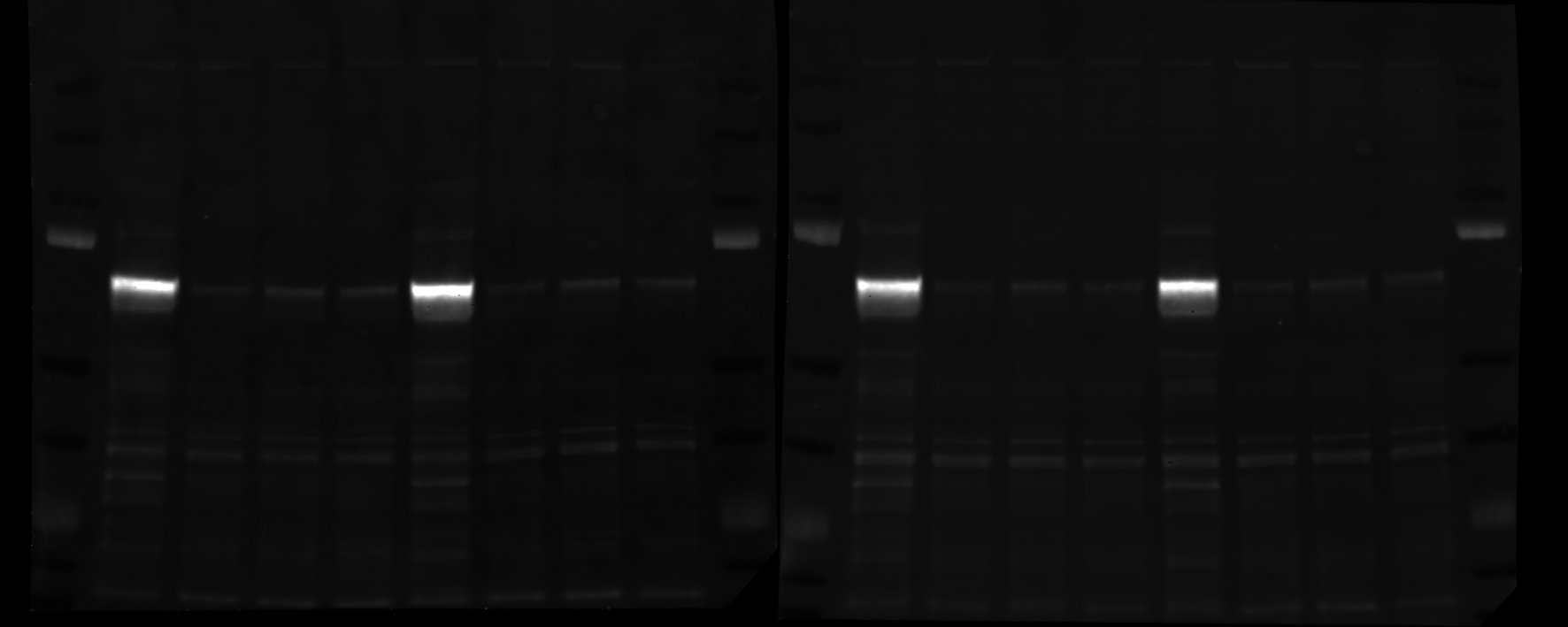

Supplement: Figure 1—source data 2. [file elife-108827-fig1-data2.zip › Figure 1C Foxc1 reblot Actin 16 bit 800.TIF]

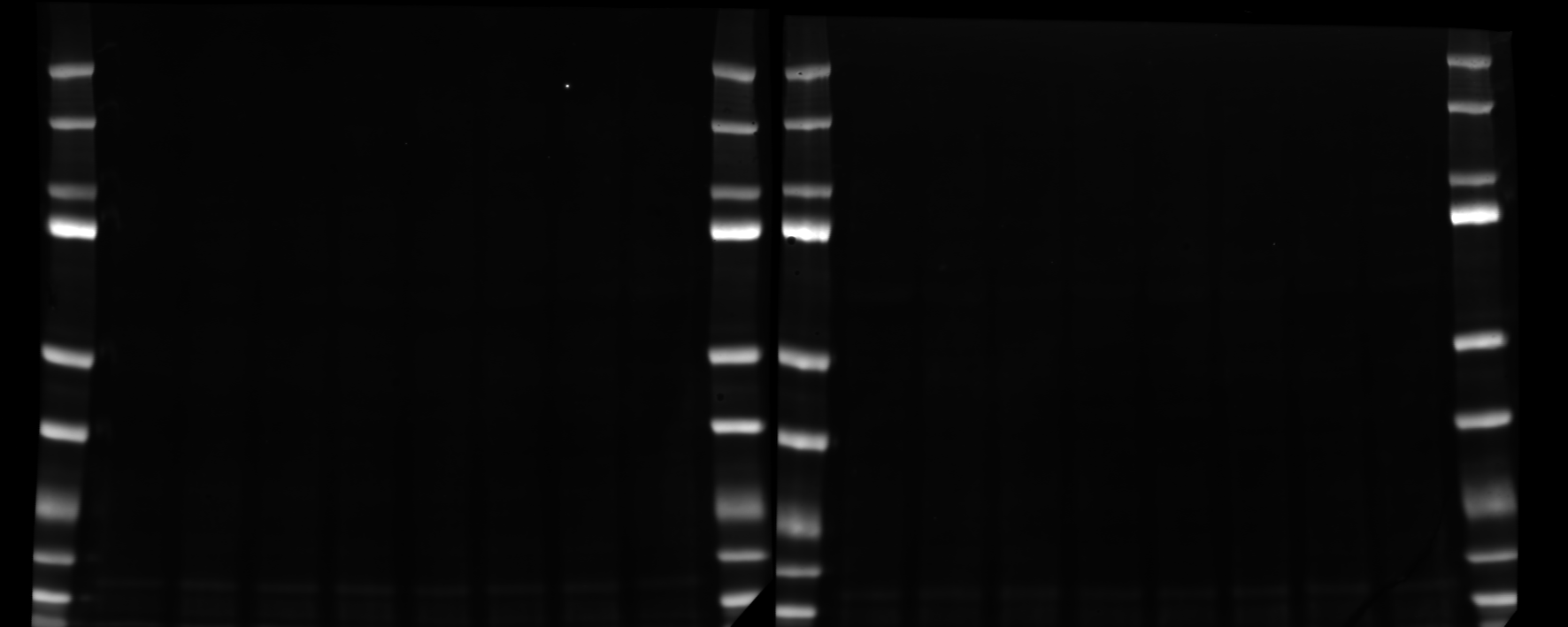

Supplement: Figure 1—source data 2. [file elife-108827-fig1-data2.zip › Figure 1C Gli1 16 bit 700.TIF]

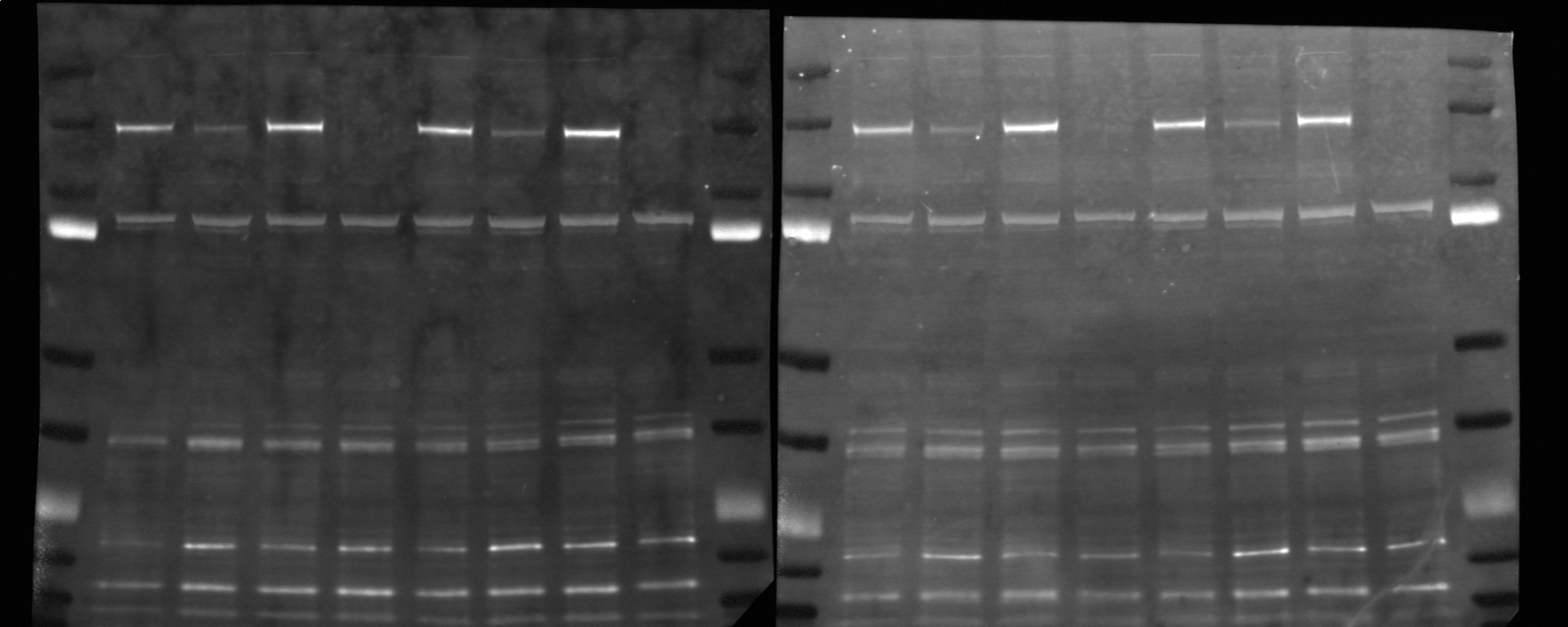

Supplement: Figure 1—source data 2. [file elife-108827-fig1-data2.zip › Figure 1C Gli1 16 bit 800.TIF]

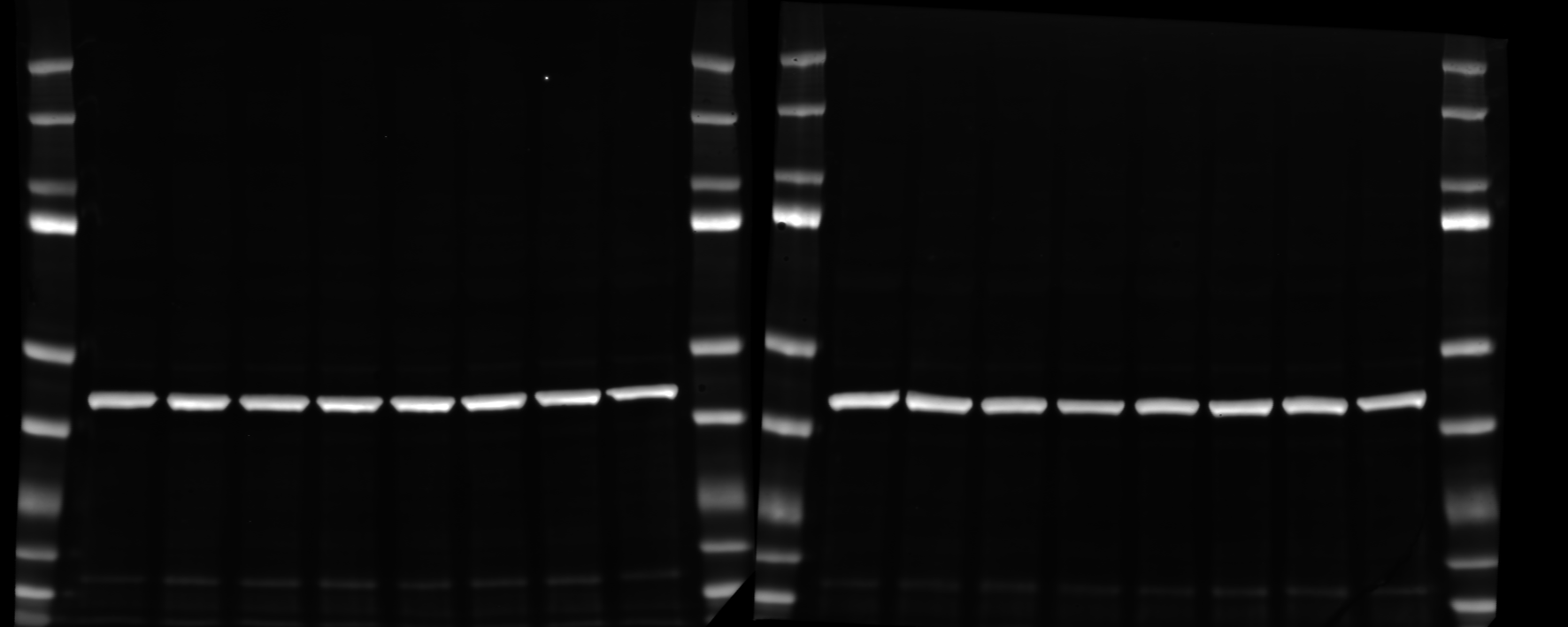

Supplement: Figure 1—source data 2. [file elife-108827-fig1-data2.zip › Figure 1C Gli1 reblot Actin 16 bit 700.TIF]

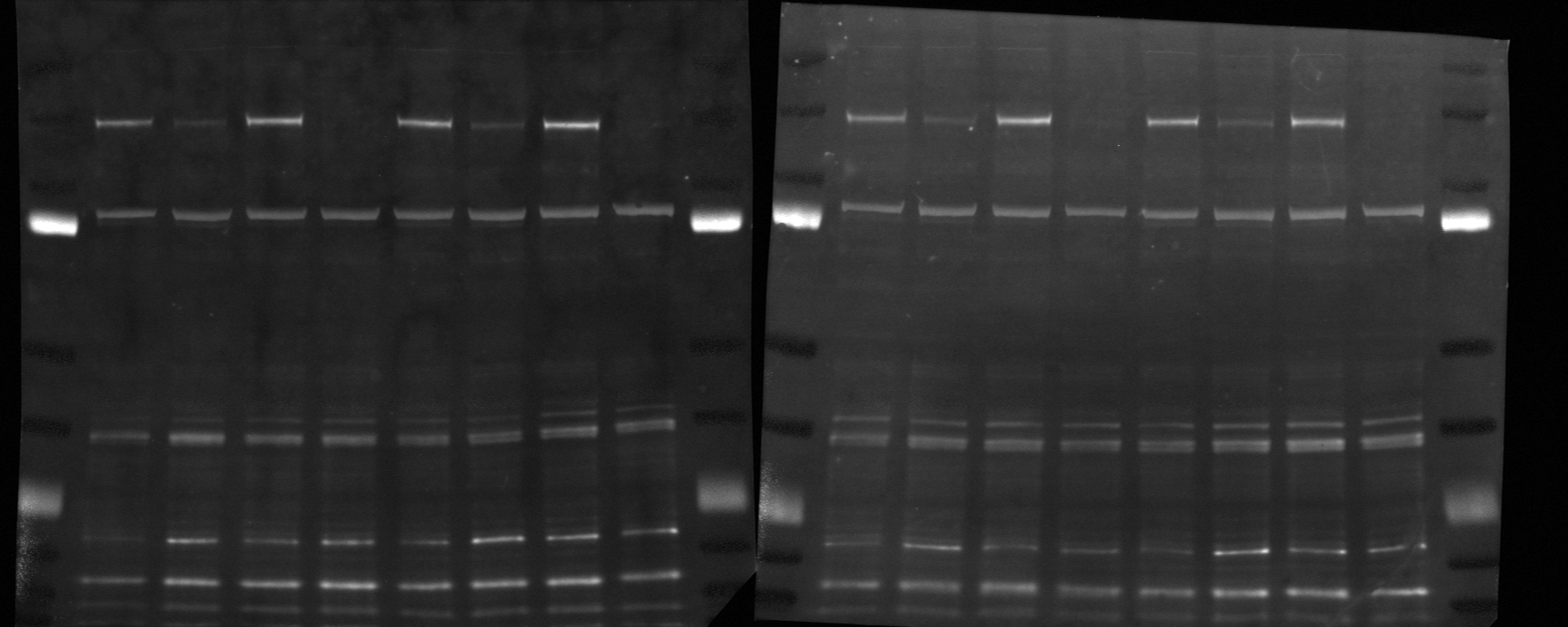

Supplement: Figure 1—source data 2. [file elife-108827-fig1-data2.zip › Figure 1C Gli1 reblot Actin 16 bit 800.TIF]

Figure 3A:

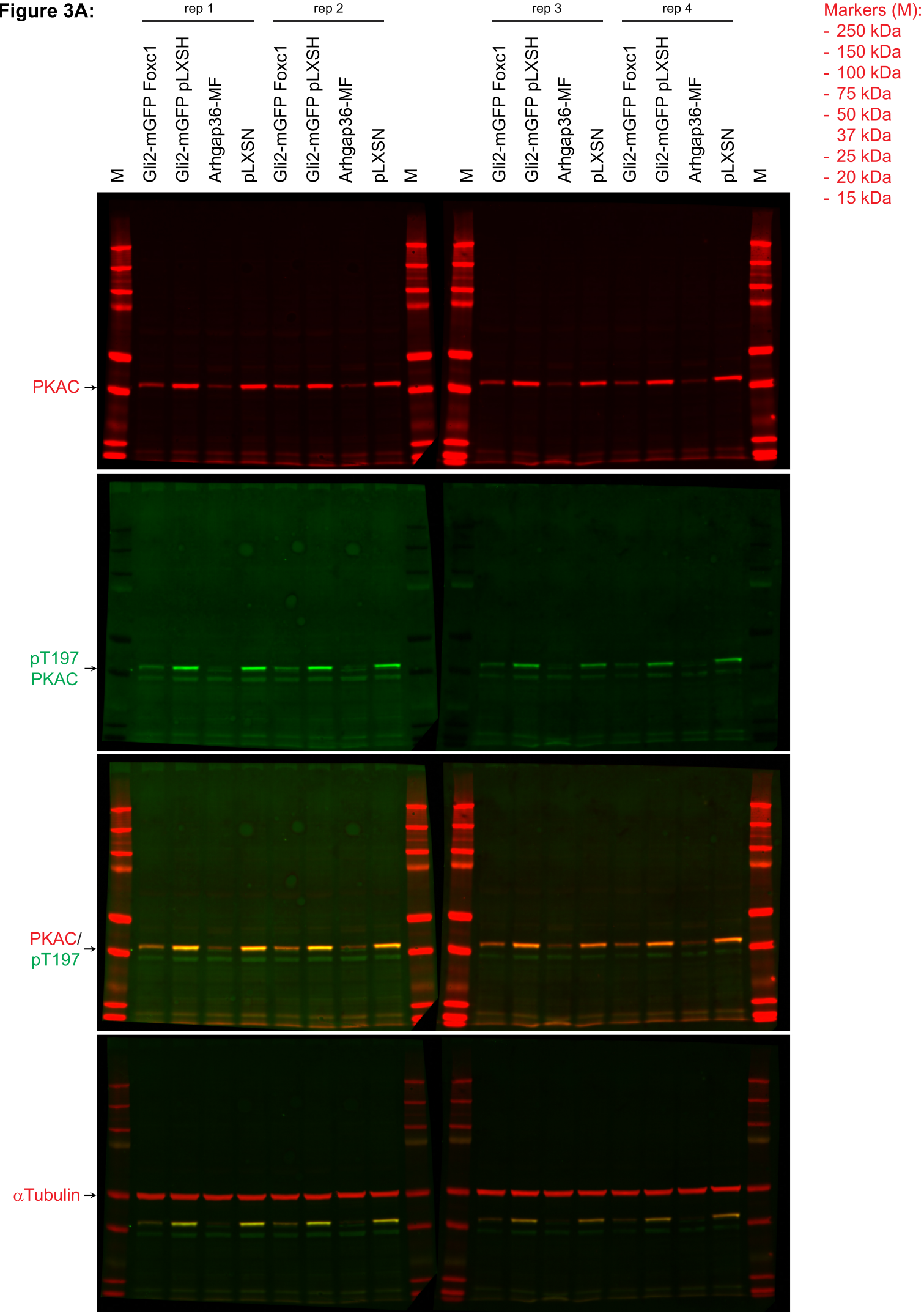

Supplement: Figure 3—source data 1. [file elife-108827-fig3-data1.zip › Figure 3-source data 1.pdf]

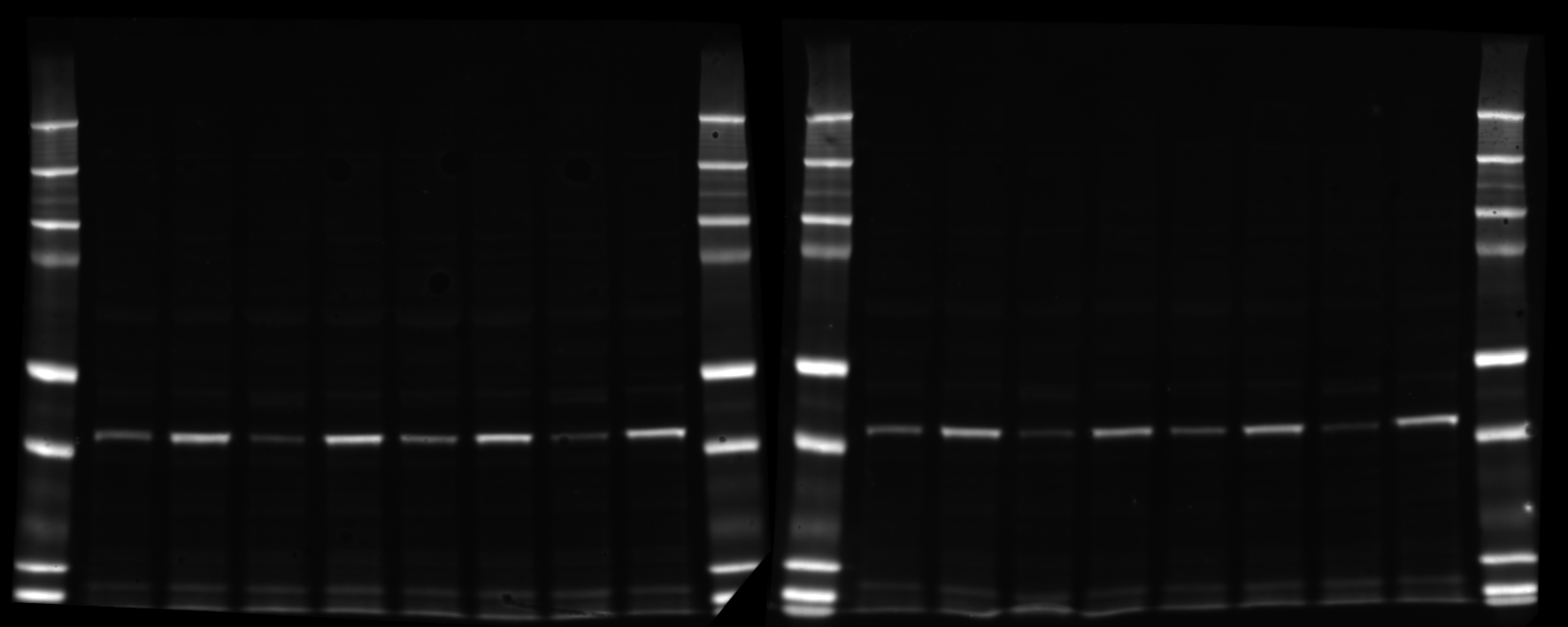

Supplement: Figure 3—source data 2. [file elife-108827-fig3-data2.zip › Figure 3A PKAC pT197 PKAC 16 bit 700.TIF]

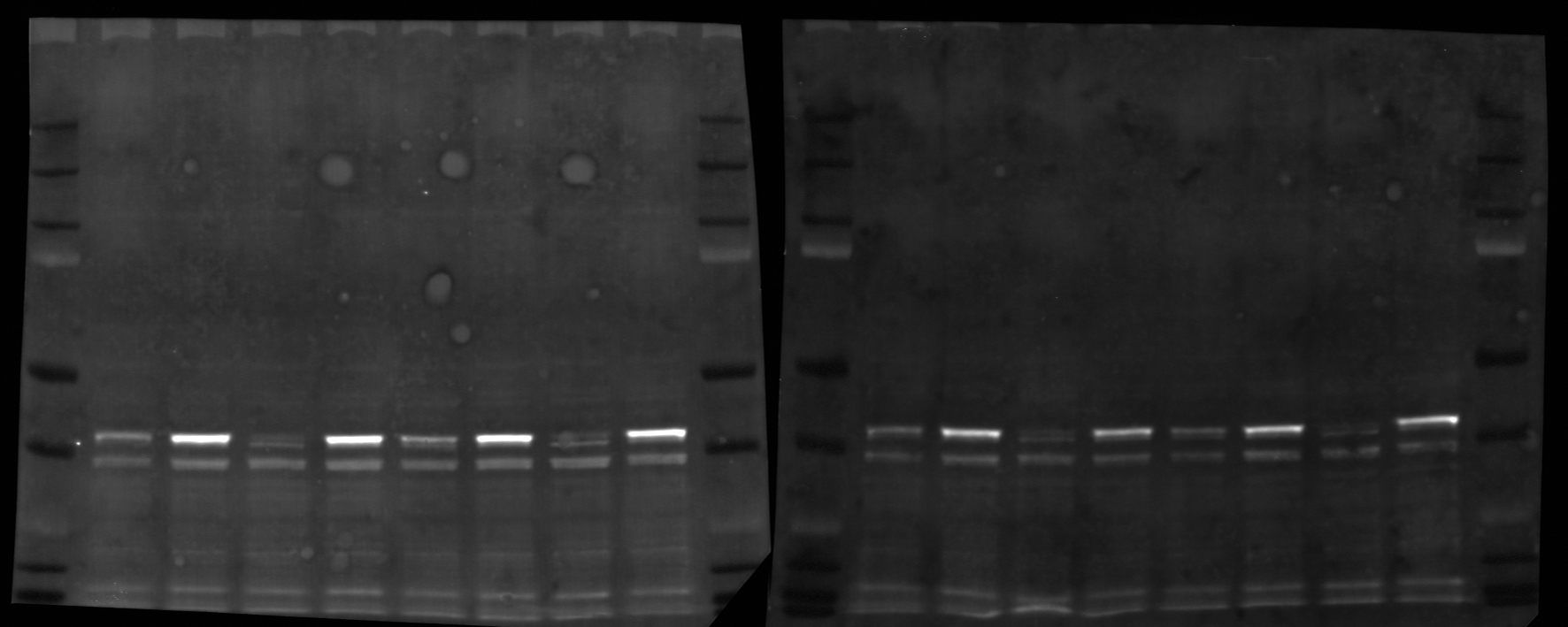

Supplement: Figure 3—source data 2. [file elife-108827-fig3-data2.zip › Figure 3A PKAC pT197 PKAC 16 bit 800.TIF]

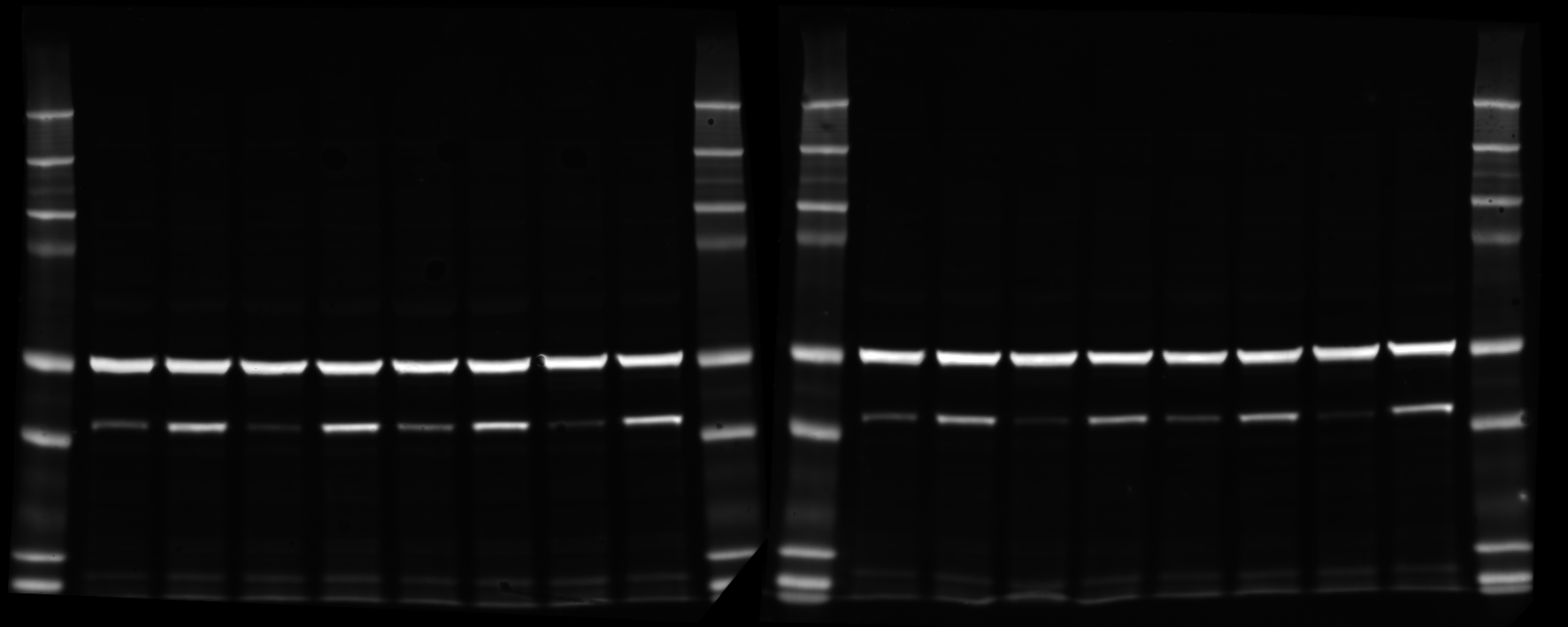

Supplement: Figure 3—source data 2. [file elife-108827-fig3-data2.zip › Figure 3A PKAC pT197 PKAC reblot aTubulin 16 bit 700.TIF]

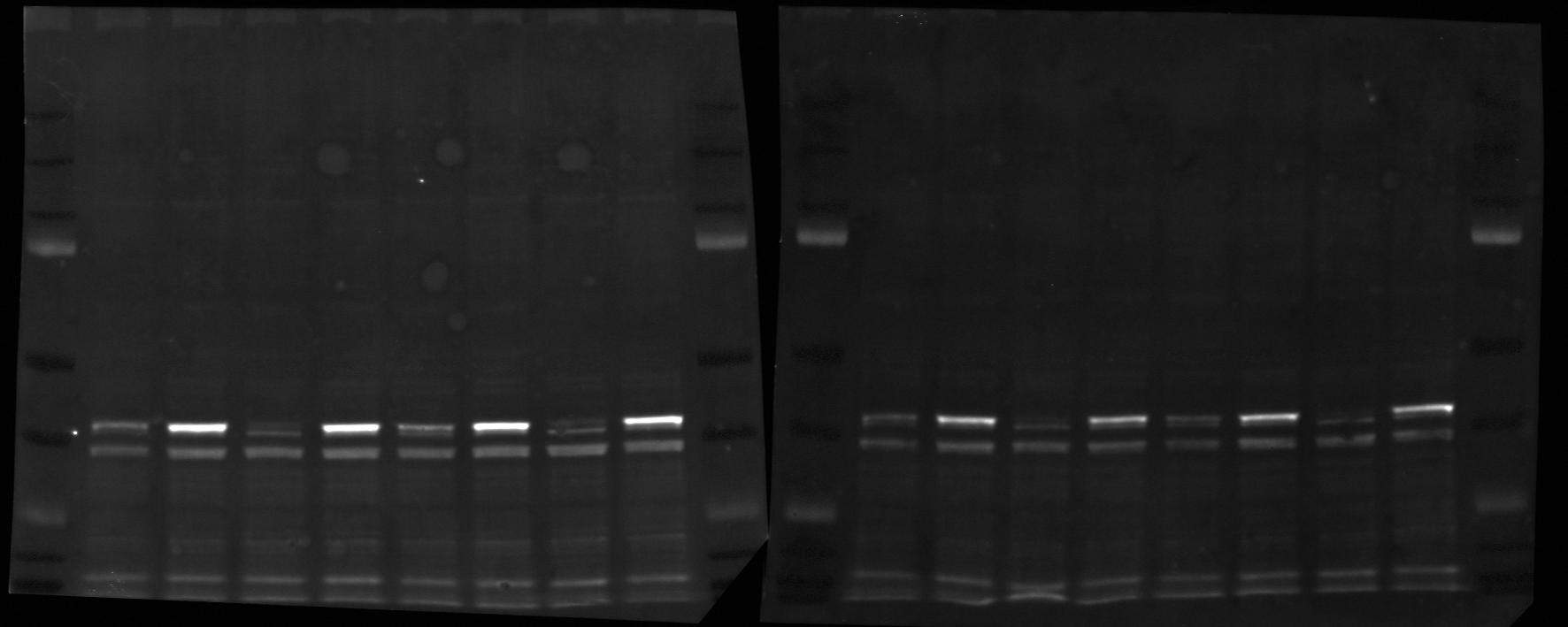

Supplement: Figure 3—source data 2. [file elife-108827-fig3-data2.zip › Figure 3A PKAC pT197 PKAC reblot aTubulin 16 bit 800.TIF]

Figure 4B:

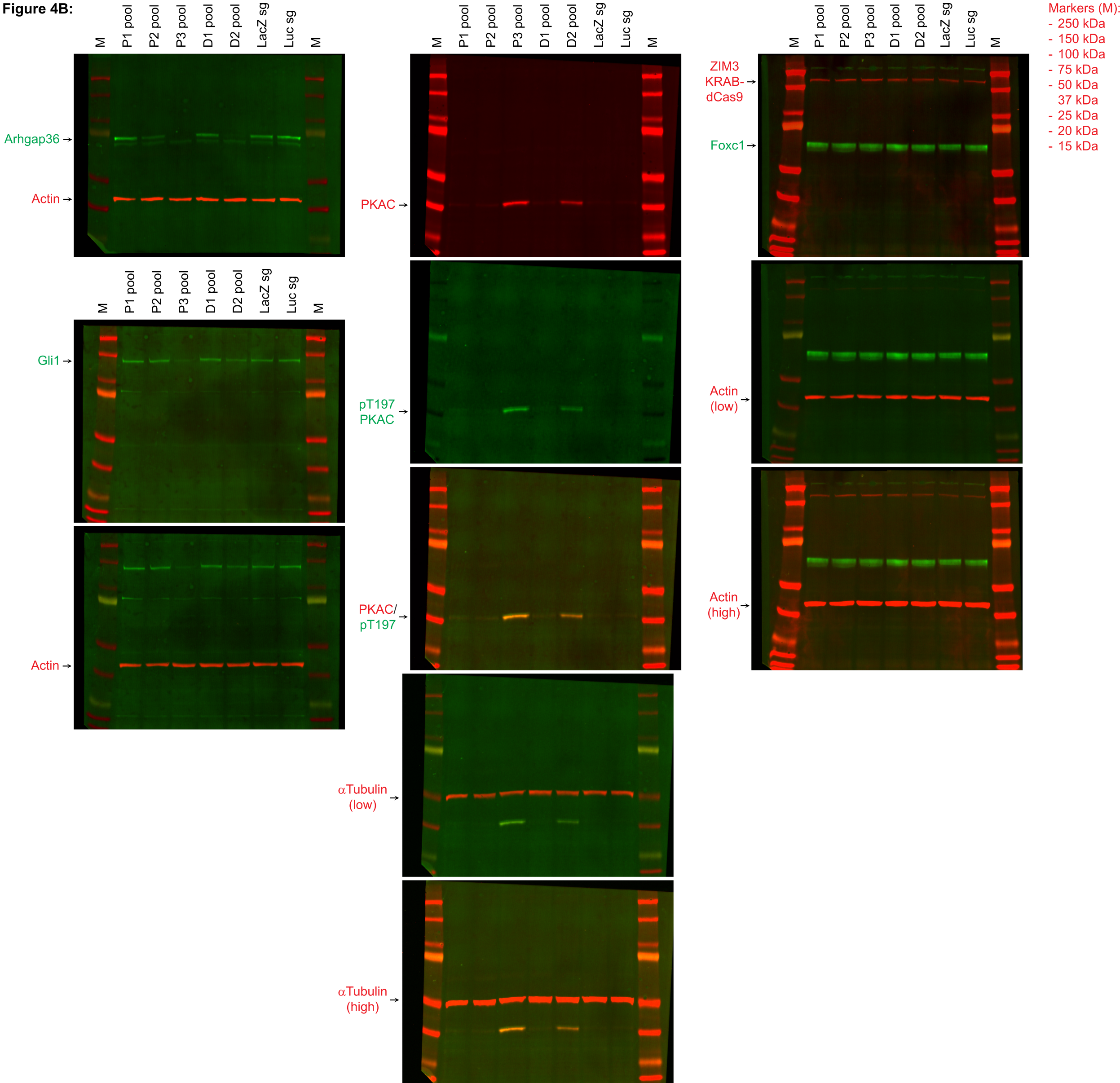

Supplement: Figure 4—source data 1. [file elife-108827-fig4-data1.zip › Figure 4-source data 1.pdf]

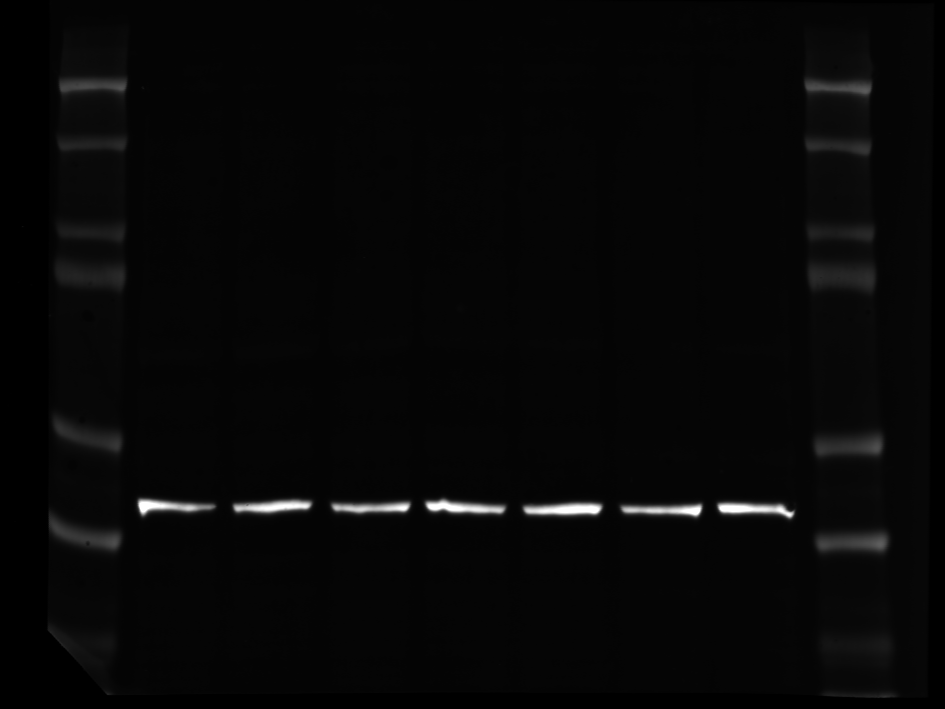

Supplement: Figure 4—source data 2. [file elife-108827-fig4-data2.zip › Figure 4B Arhgap36 Actin 16 bit 700.TIF]

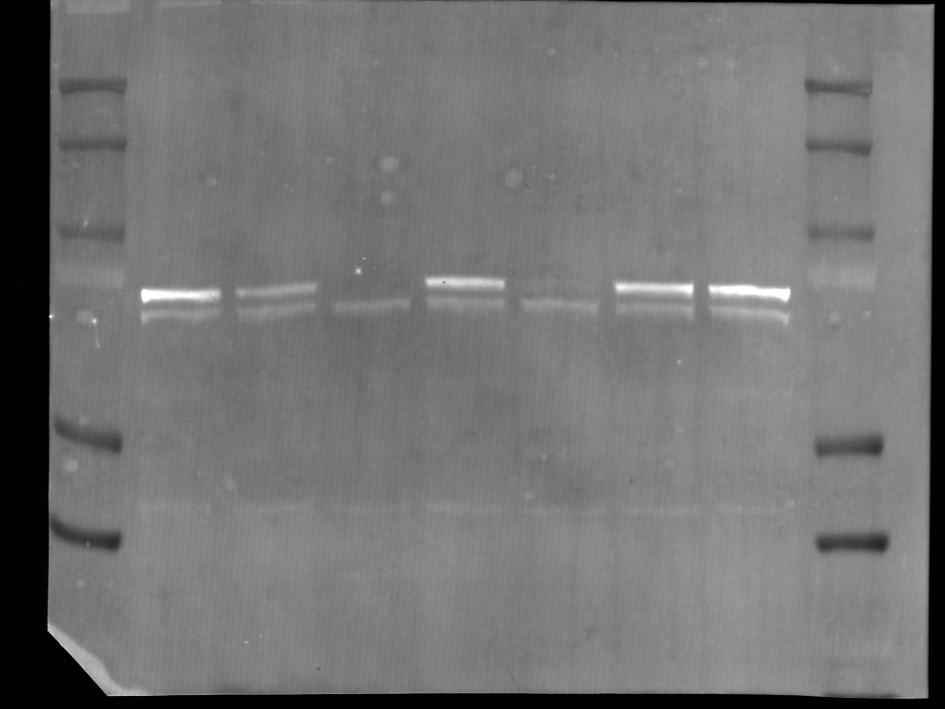

Supplement: Figure 4—source data 2. [file elife-108827-fig4-data2.zip › Figure 4B Arhgap36 Actin 16 bit 800.TIF]

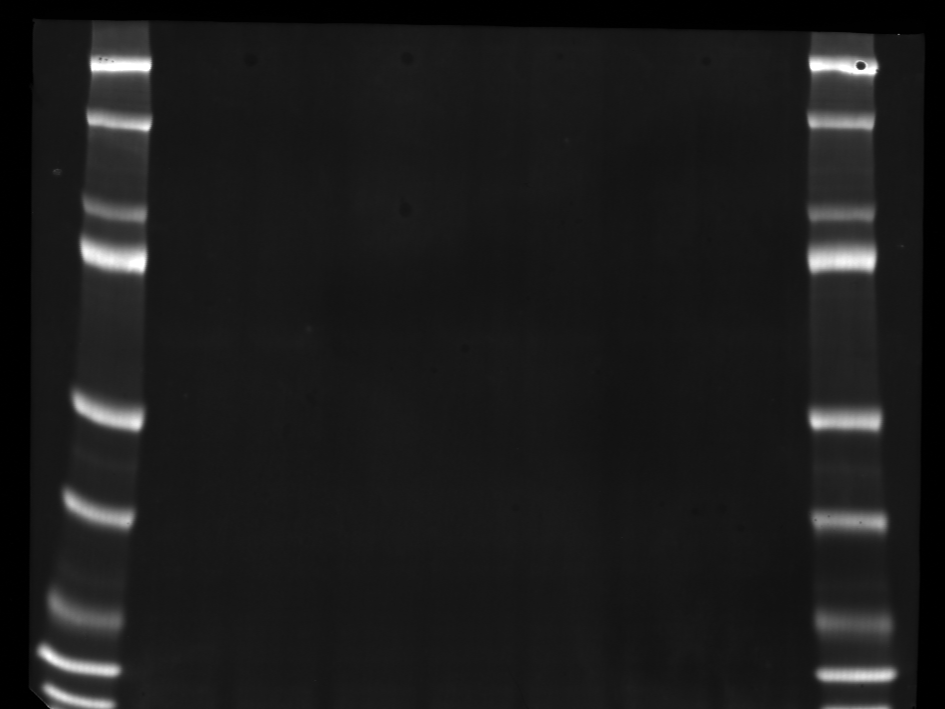

Supplement: Figure 4—source data 2. [file elife-108827-fig4-data2.zip › Figure 4B Gli1 16 bit 700.TIF]

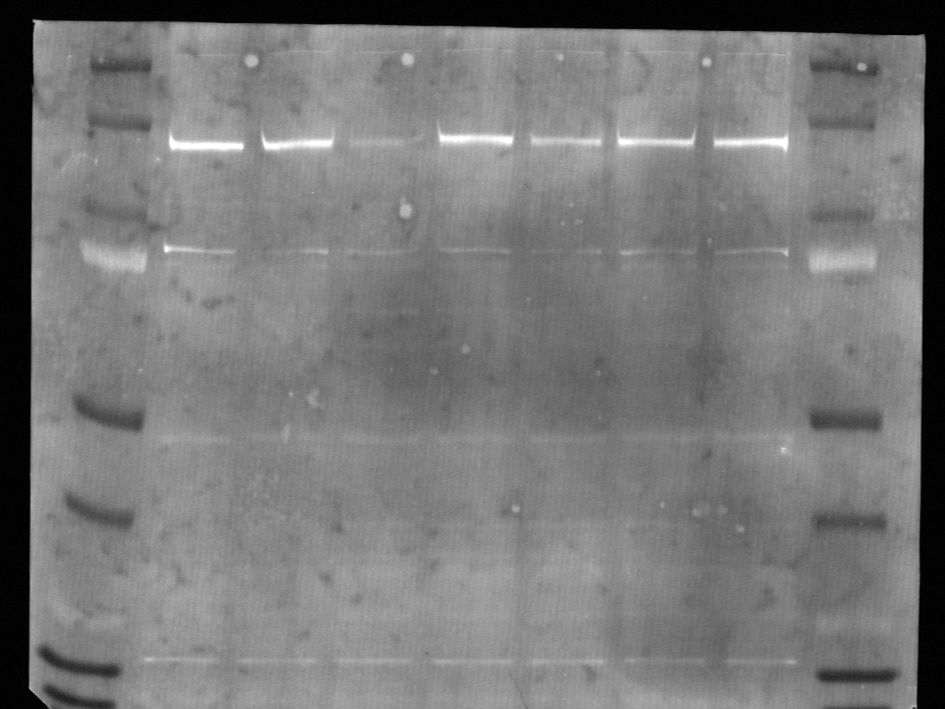

Supplement: Figure 4—source data 2. [file elife-108827-fig4-data2.zip › Figure 4B Gli1 16 bit 800.TIF]

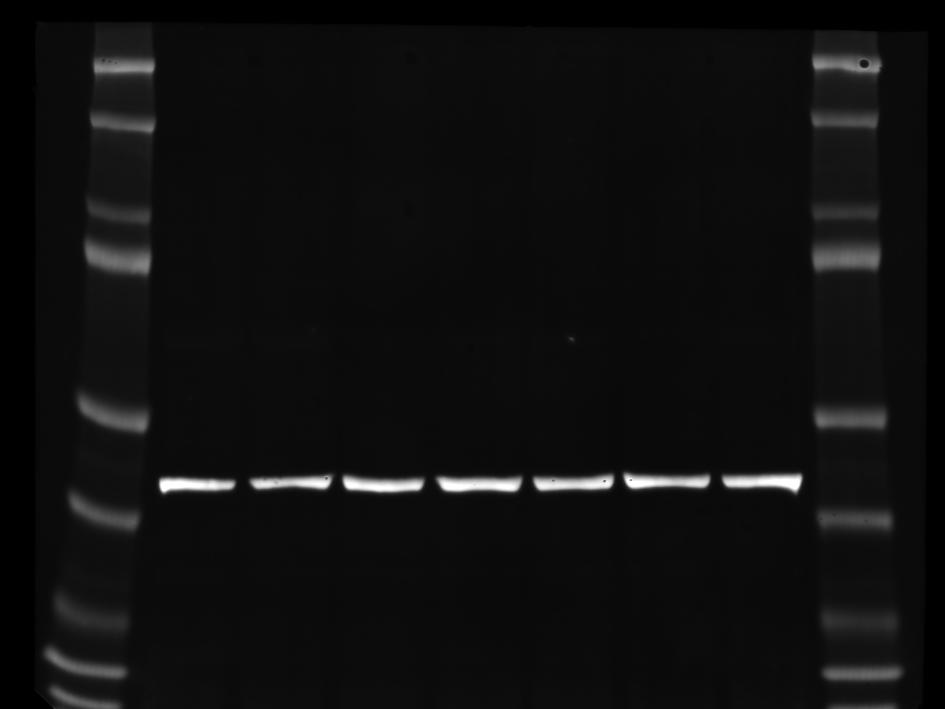

Supplement: Figure 4—source data 2. [file elife-108827-fig4-data2.zip › Figure 4B Gli1 reblot Actin 16 bit 700.TIF]

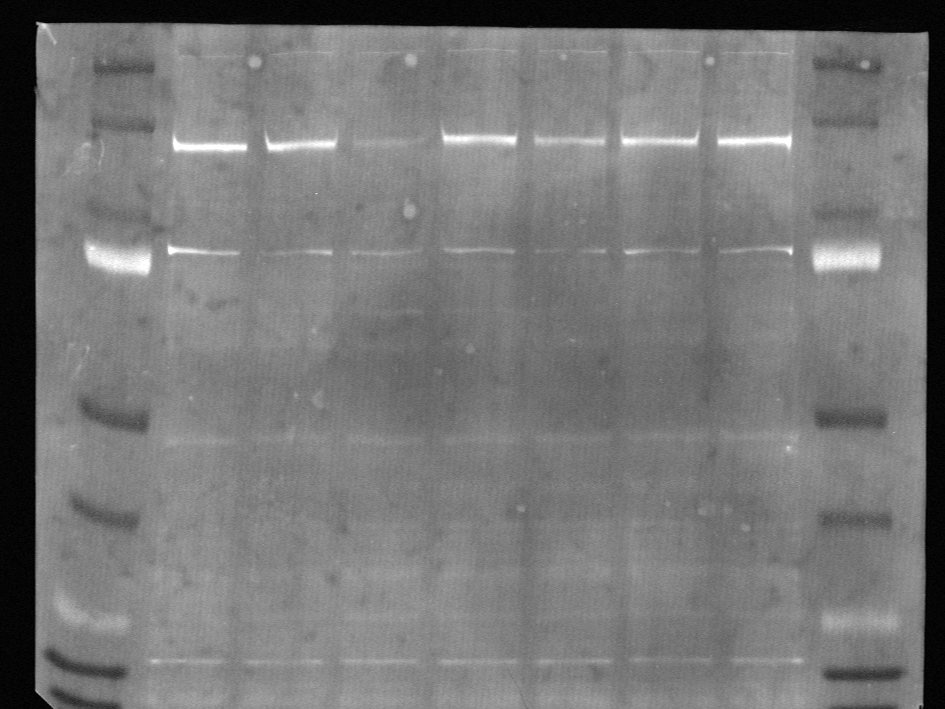

Supplement: Figure 4—source data 2. [file elife-108827-fig4-data2.zip › Figure 4B Gli1 reblot Actin 16 bit 800.TIF]

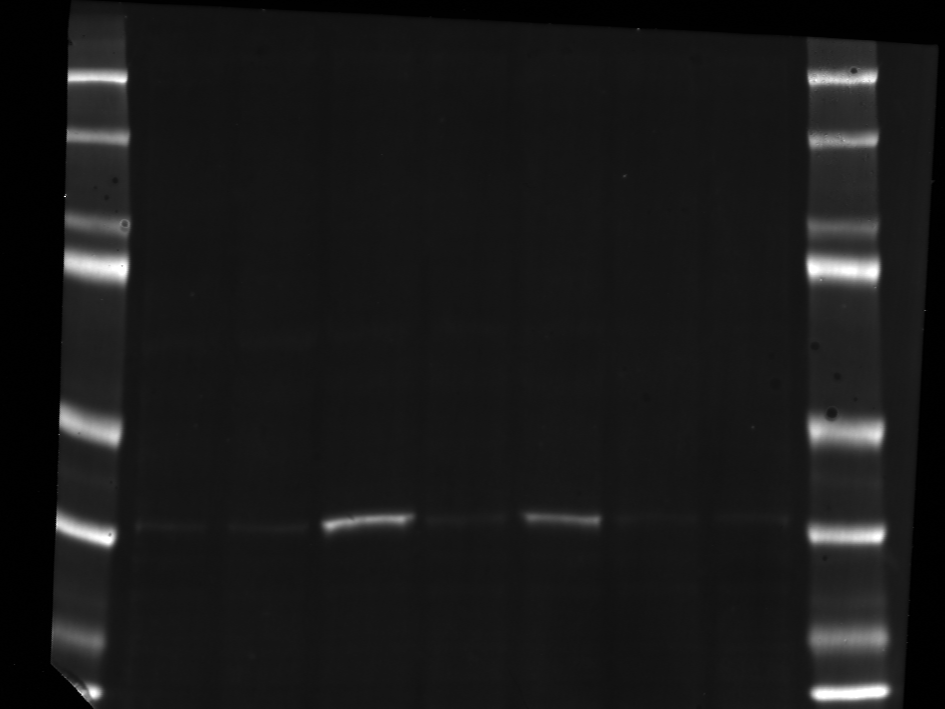

Supplement: Figure 4—source data 2. [file elife-108827-fig4-data2.zip › Figure 4B PKAC pT197 PKAC 16 bit 700.TIF]

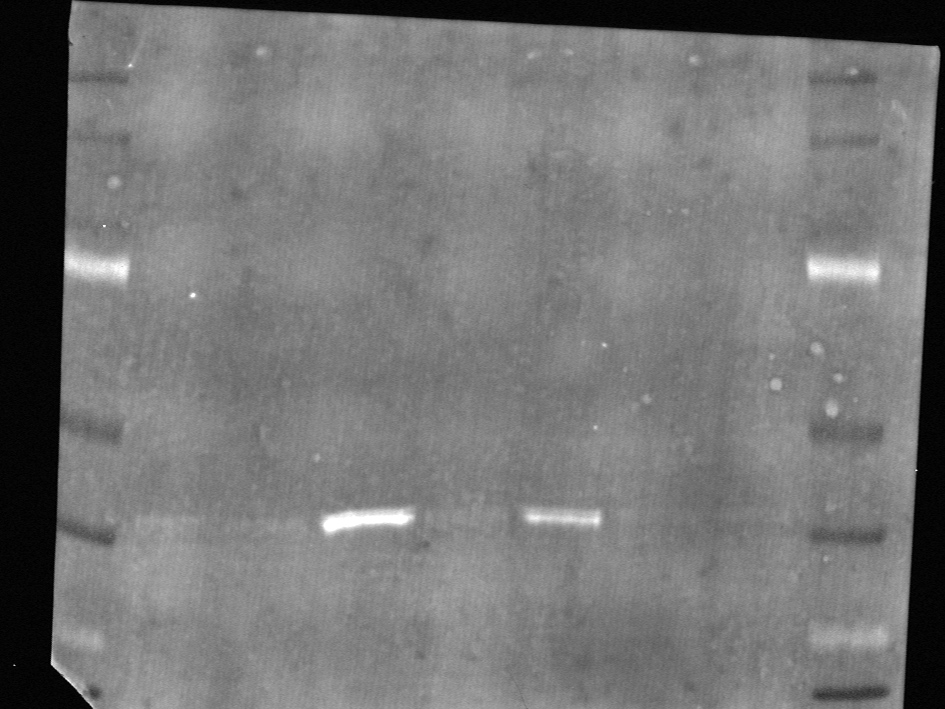

Supplement: Figure 4—source data 2. [file elife-108827-fig4-data2.zip › Figure 4B PKAC pT197 PKAC 16 bit 800.TIF]

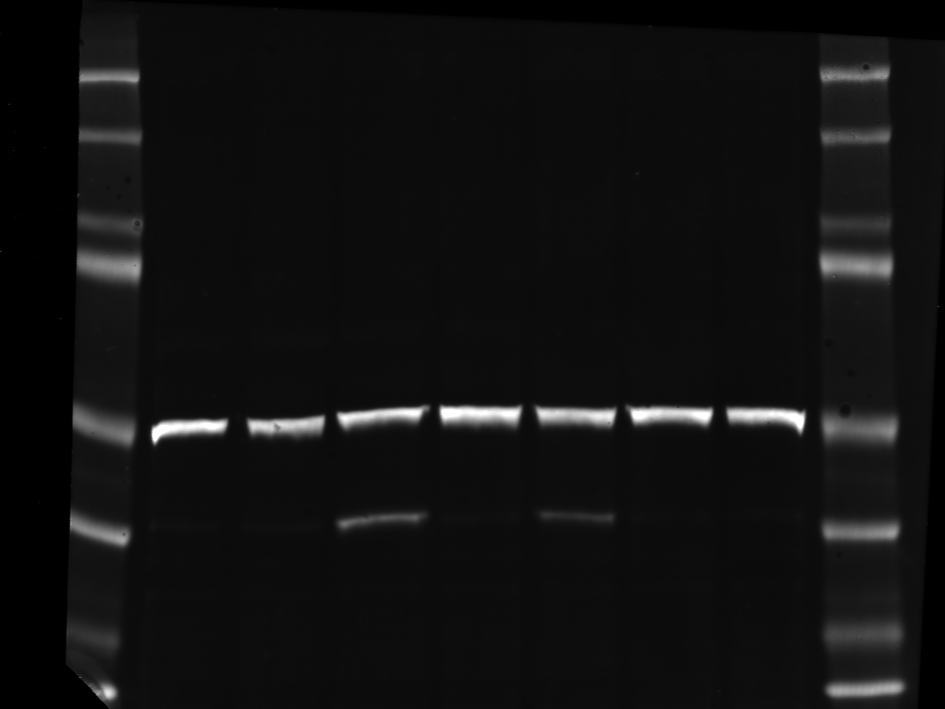

Supplement: Figure 4—source data 2. [file elife-108827-fig4-data2.zip › Figure 4B PKAC pT197 PKAC reblot Tubulin 16 bit 700.TIF]

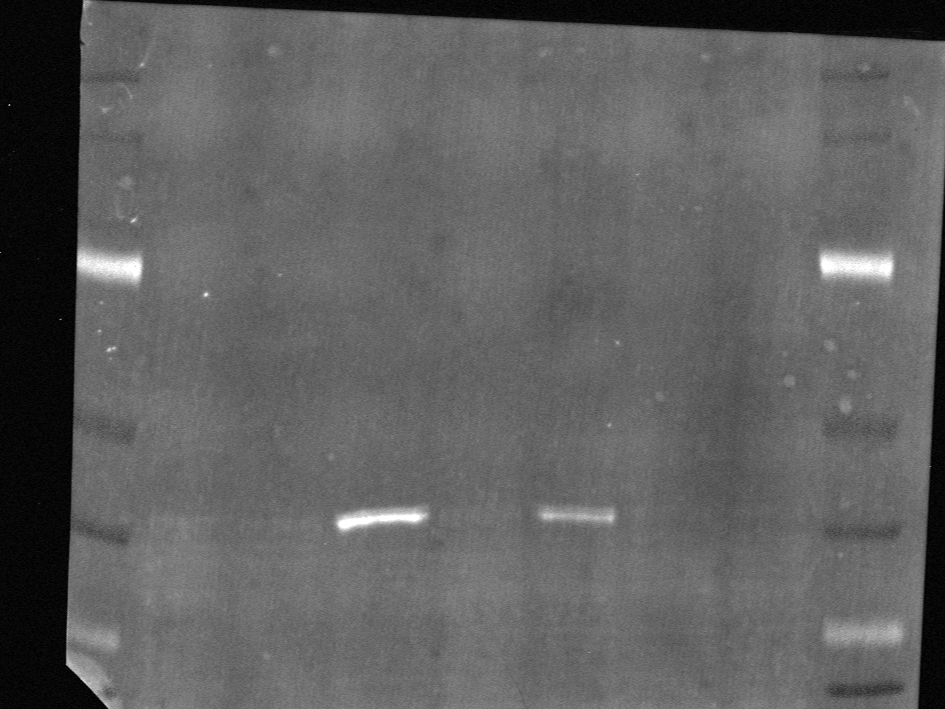

Supplement: Figure 4—source data 2. [file elife-108827-fig4-data2.zip › Figure 4B PKAC pT197 PKAC reblot Tubulin 16 bit 800.TIF]

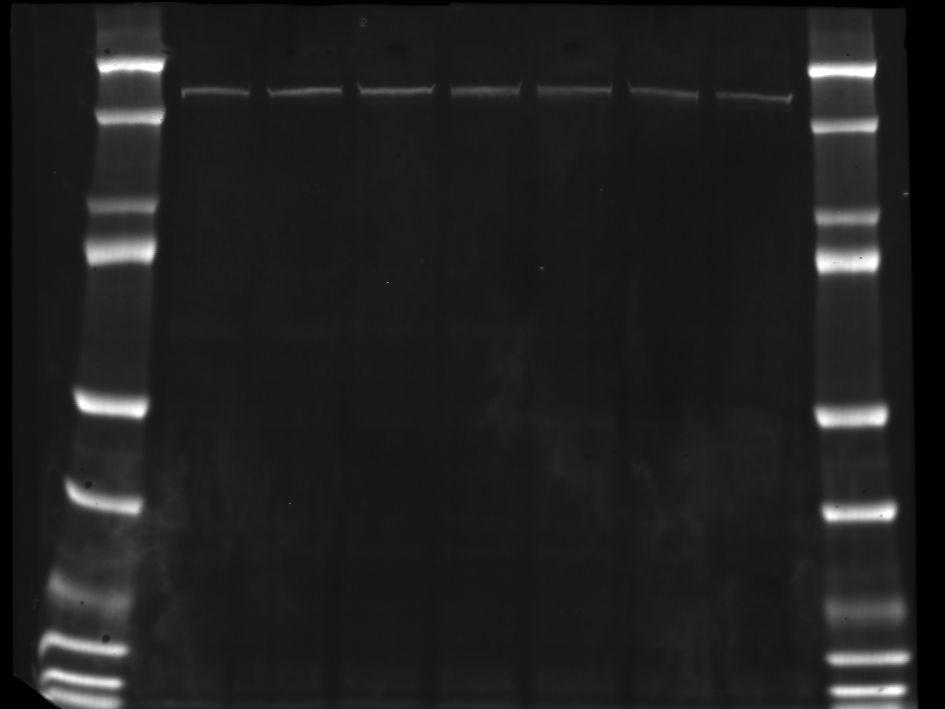

Supplement: Figure 4—source data 2. [file elife-108827-fig4-data2.zip › Figure 4B ZIM3-KRAB-dCas9 Foxc1 16 bit 700.TIF]

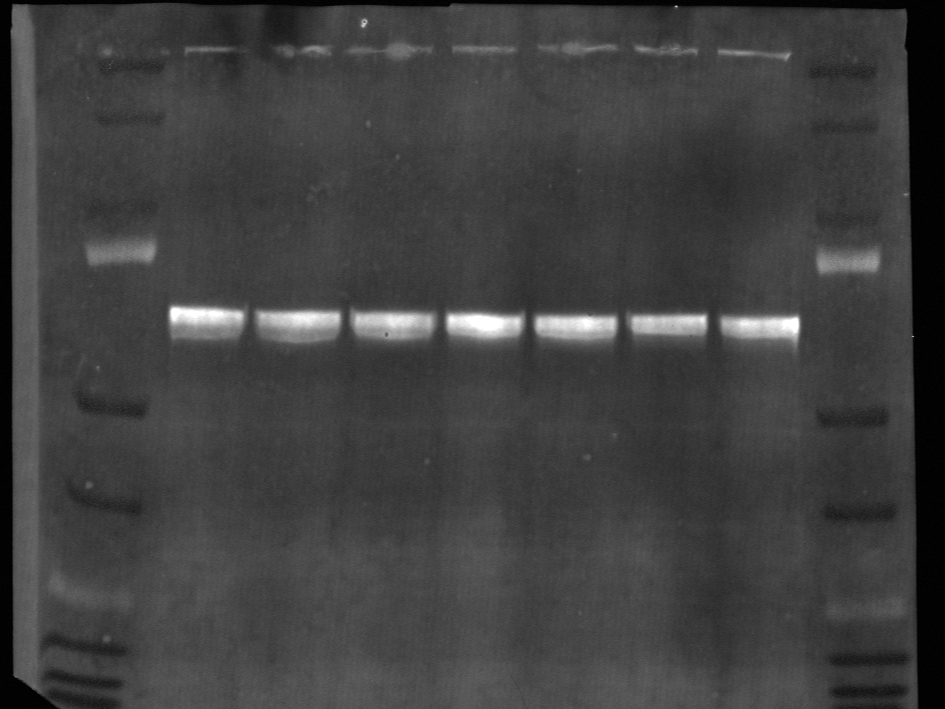

Supplement: Figure 4—source data 2. [file elife-108827-fig4-data2.zip › Figure 4B ZIM3-KRAB-dCas9 Foxc1 16 bit 800.TIF]

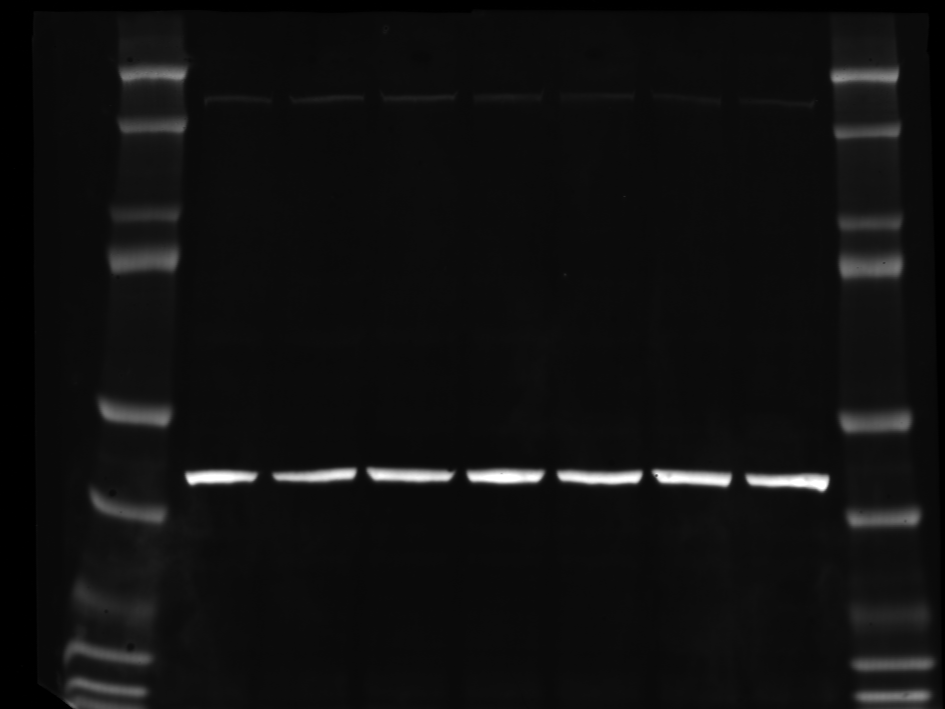

Supplement: Figure 4—source data 2. [file elife-108827-fig4-data2.zip › Figure 4B ZIM3-KRAB-dCas9 Foxc1 reblot Actin 16 bit 700.TIF]

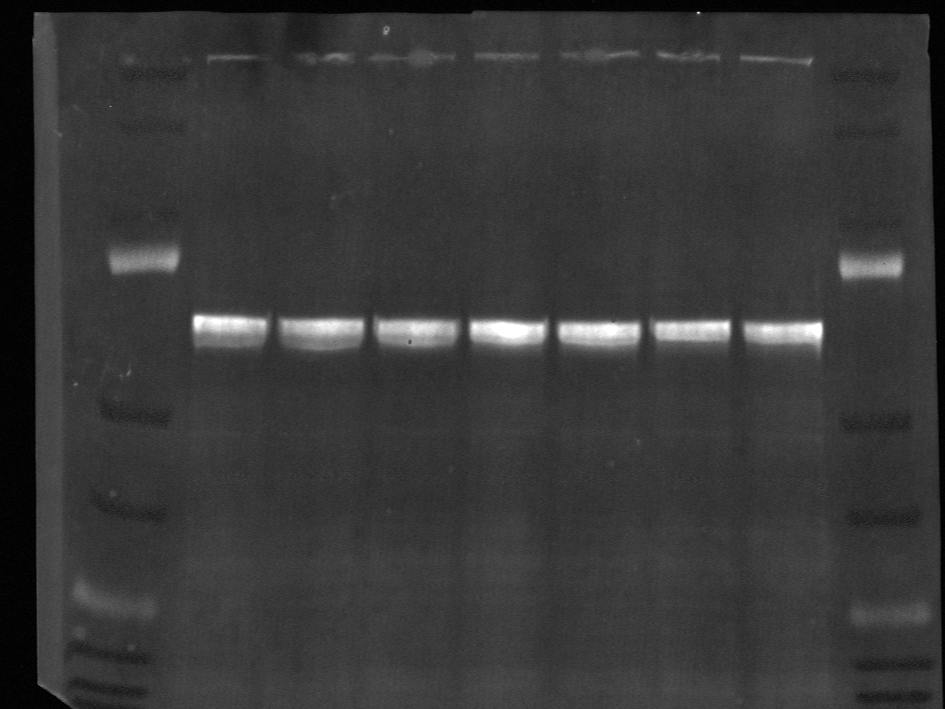

Supplement: Figure 4—source data 2. [file elife-108827-fig4-data2.zip › Figure 4B ZIM3-KRAB-dCas9 Foxc1 reblot Actin 16 bit 800.TIF]

**Figure 5B:**

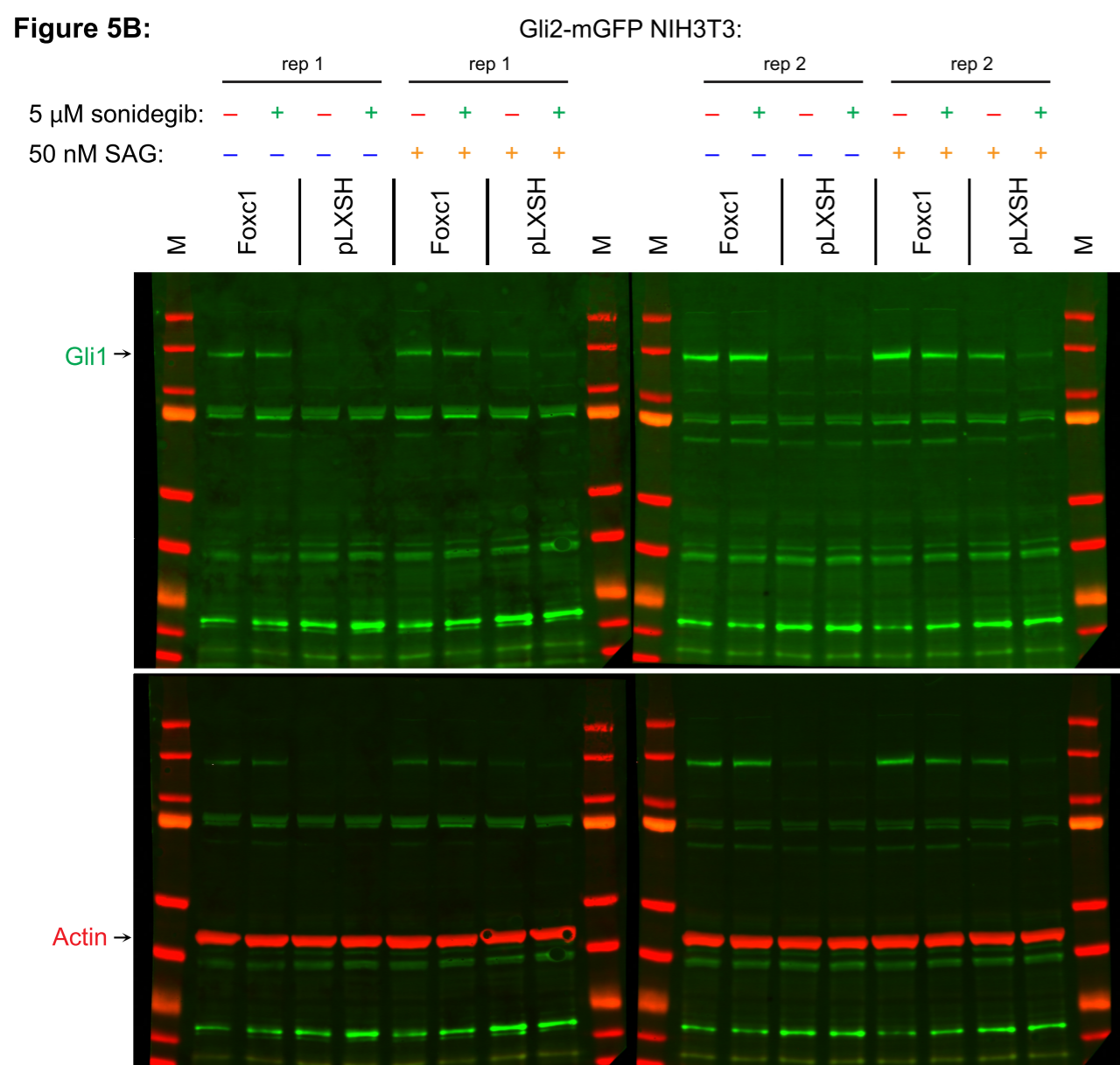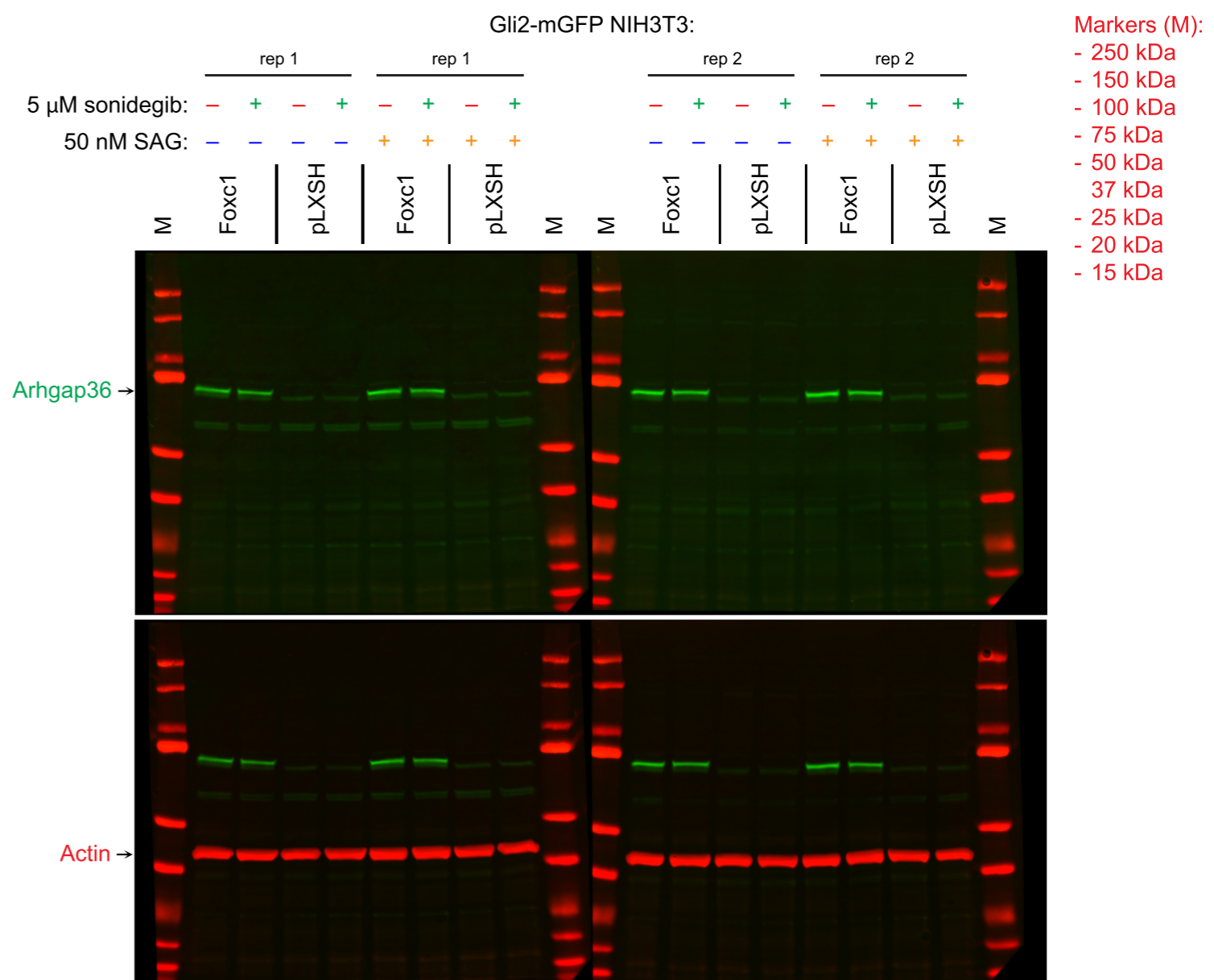

**Figures 5B  
and S9A:**

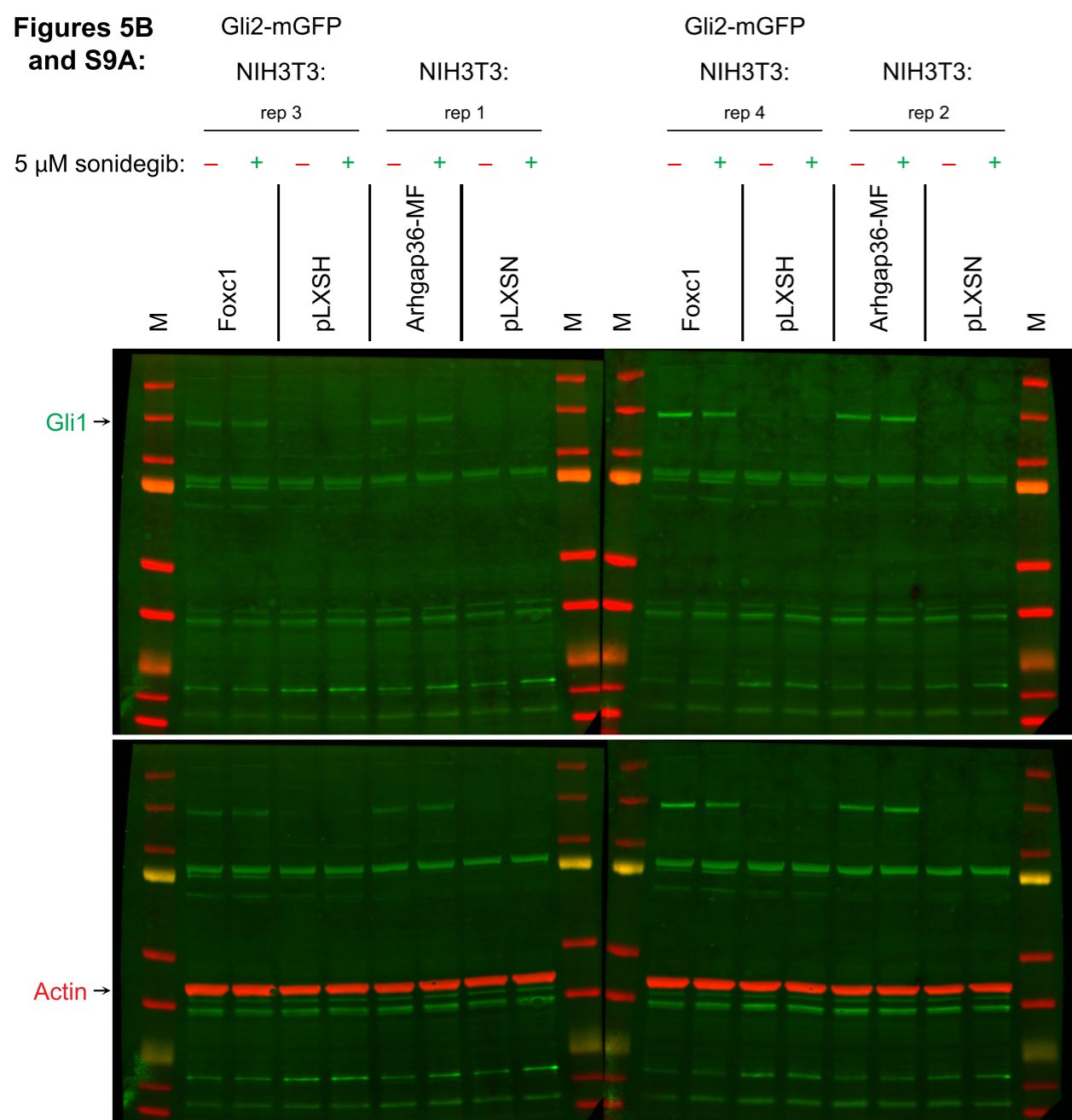

Supplement: Figure 5—source data 1. [file elife-108827-fig5-data1.zip › Figure 5-source data 1.pdf]

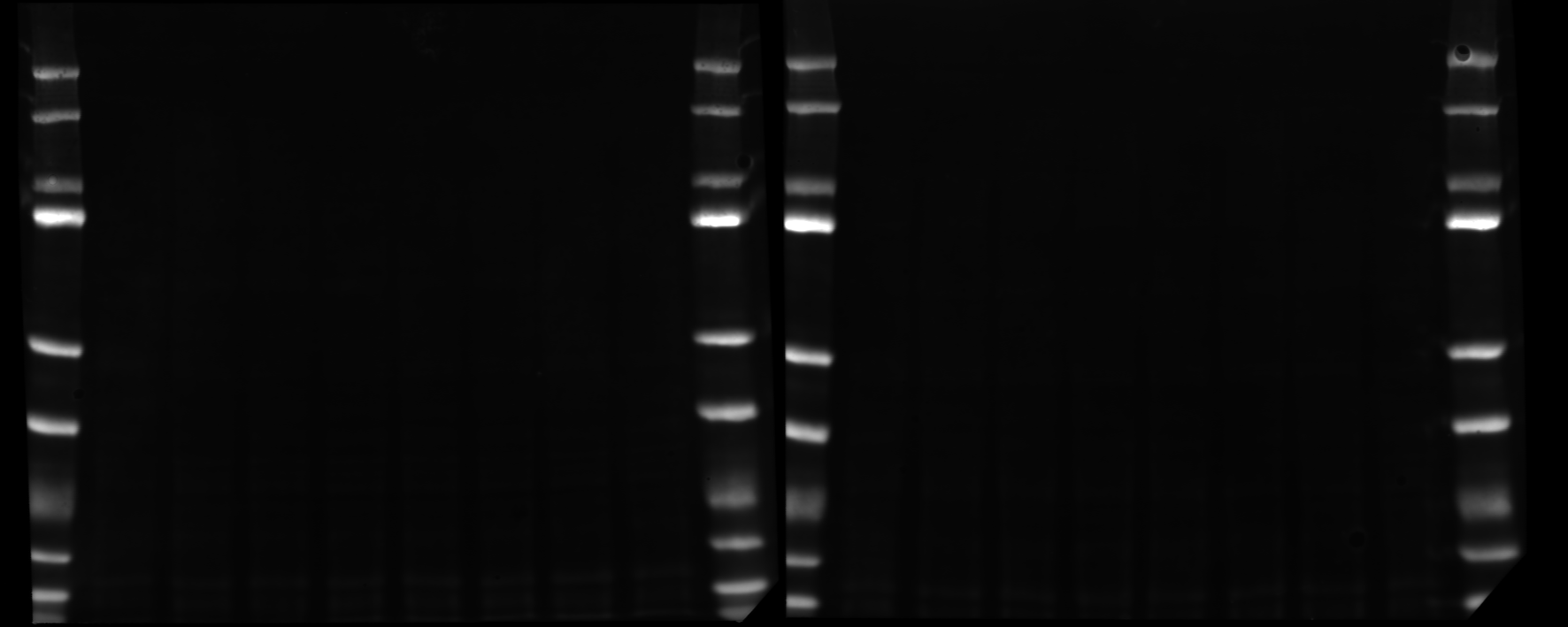

Supplement: Figure 5—source data 2. [file elife-108827-fig5-data2.zip › Figure 5B Arhgap36 16 bit 700.TIF]

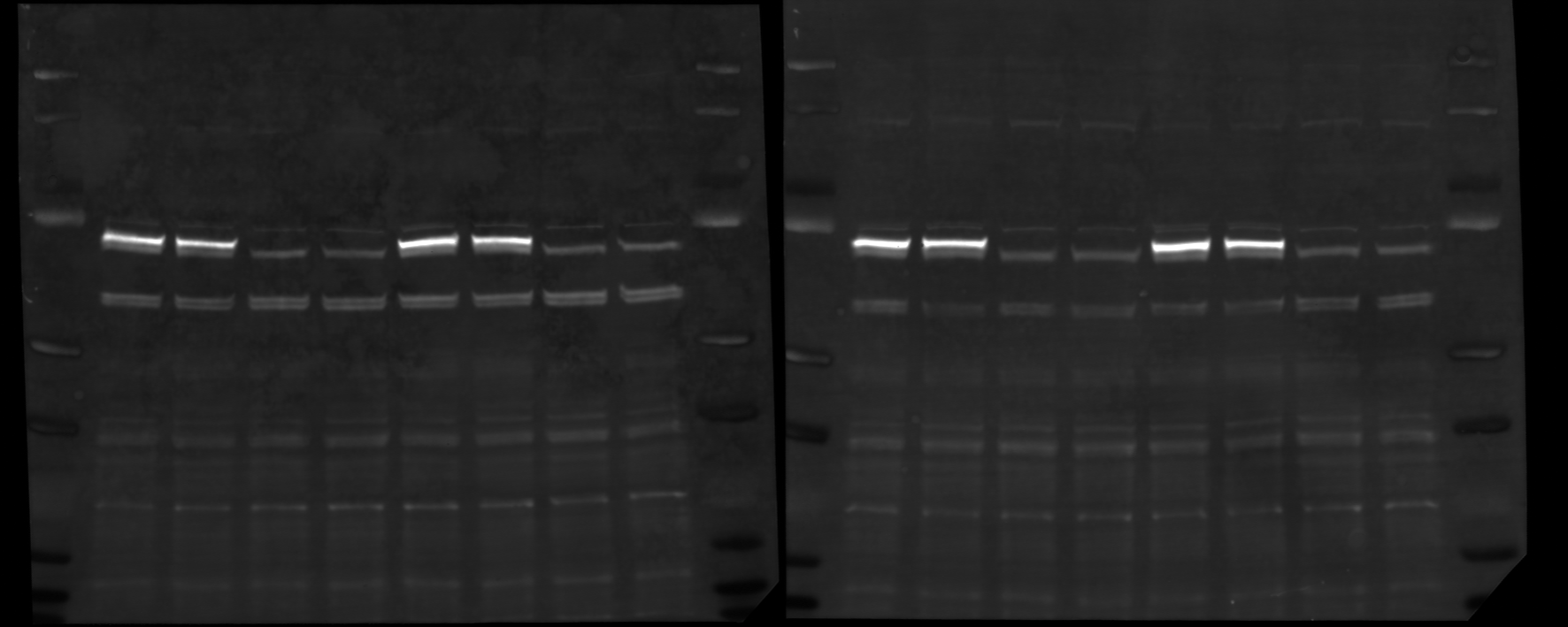

Supplement: Figure 5—source data 2. [file elife-108827-fig5-data2.zip › Figure 5B Arhgap36 16 bit 800.TIF]

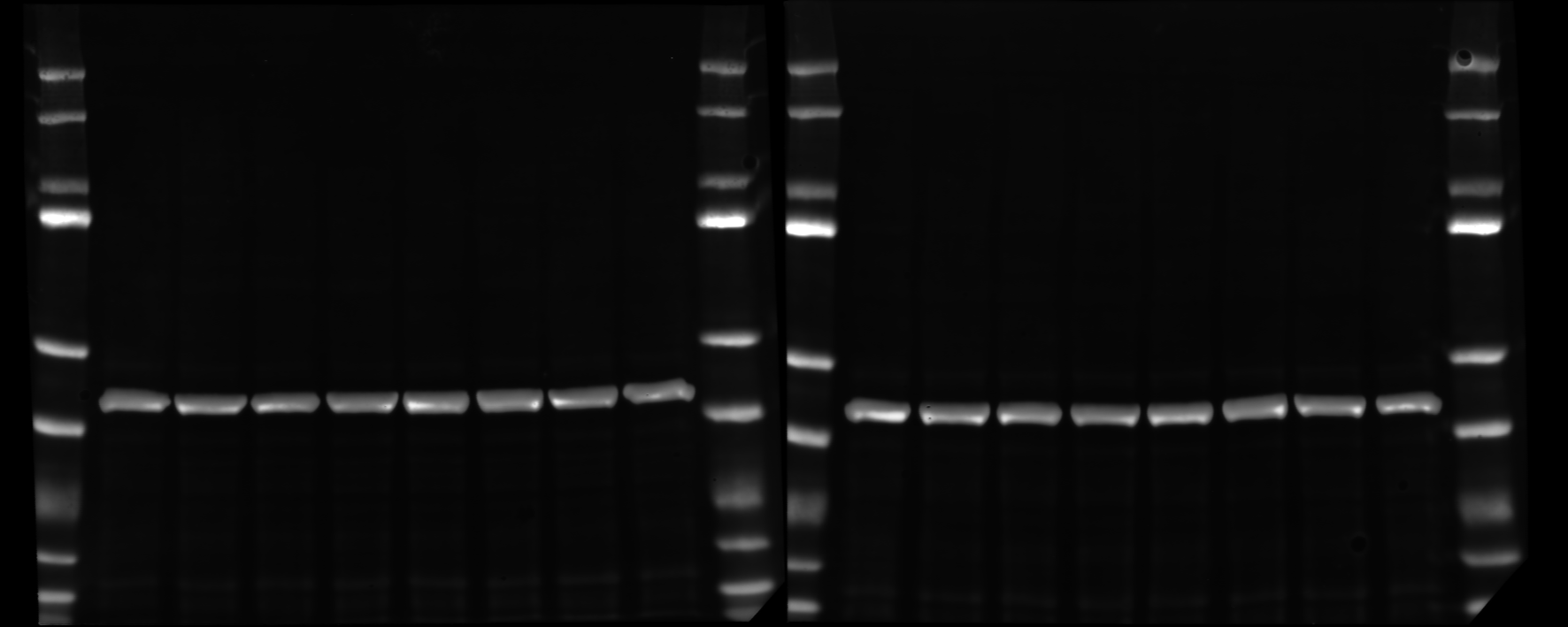

Supplement: Figure 5—source data 2. [file elife-108827-fig5-data2.zip › Figure 5B Arhgap36 reblot Actin 16 bit 700.TIF]

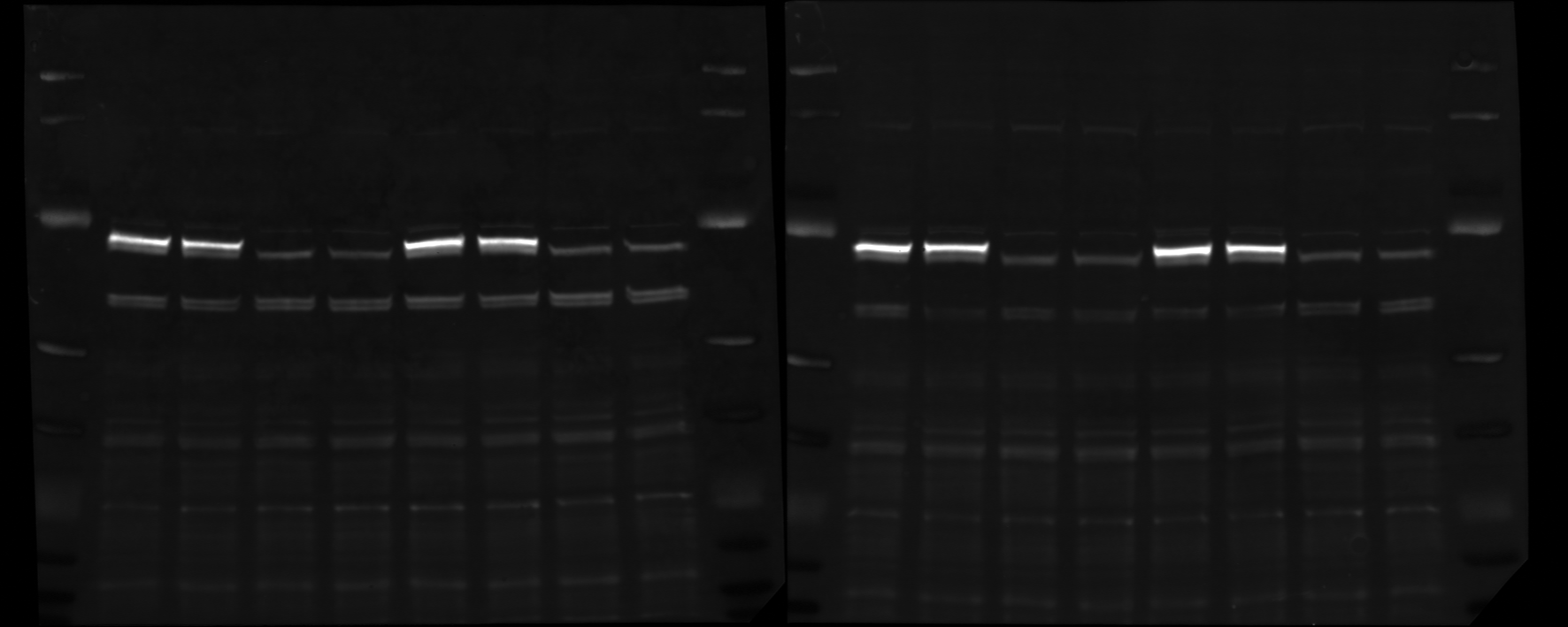

Supplement: Figure 5—source data 2. [file elife-108827-fig5-data2.zip › Figure 5B Arhgap36 reblot Actin 16 bit 800.TIF]

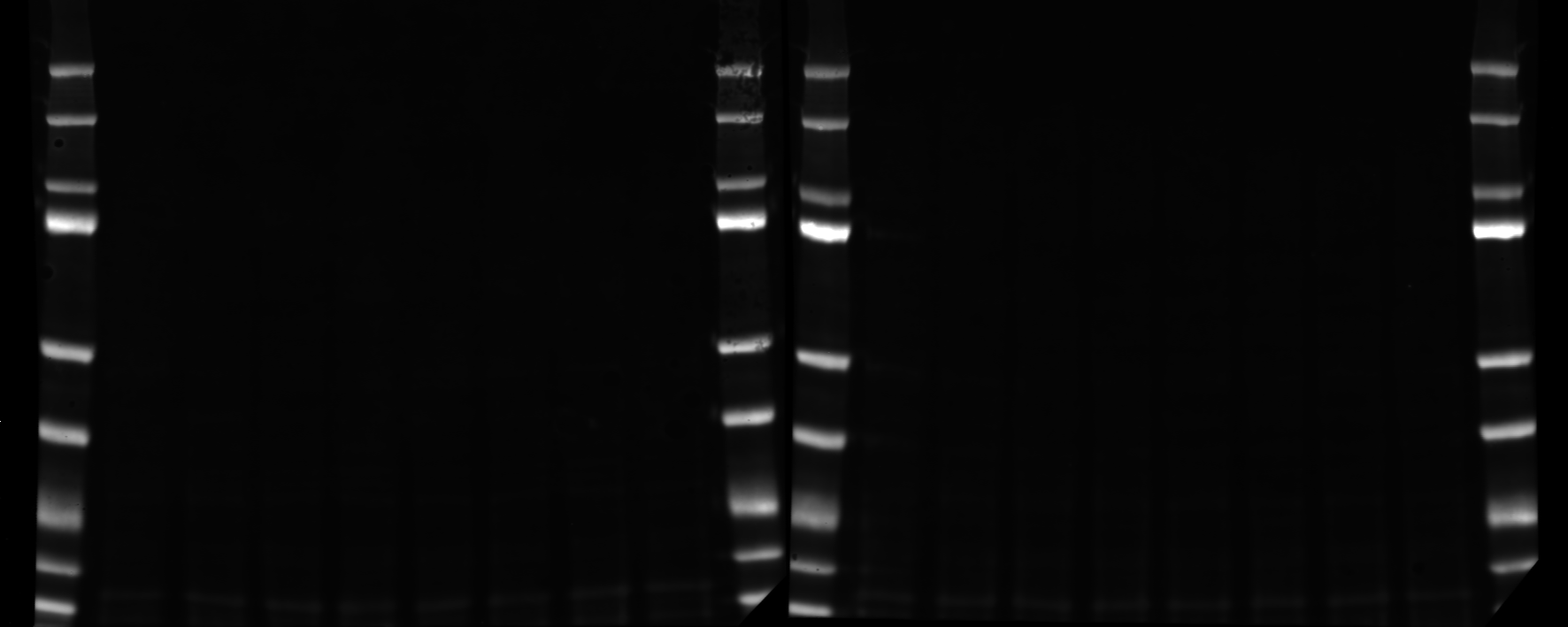

Supplement: Figure 5—source data 2. [file elife-108827-fig5-data2.zip › Figure 5B Gli1 16 bit 700.TIF]

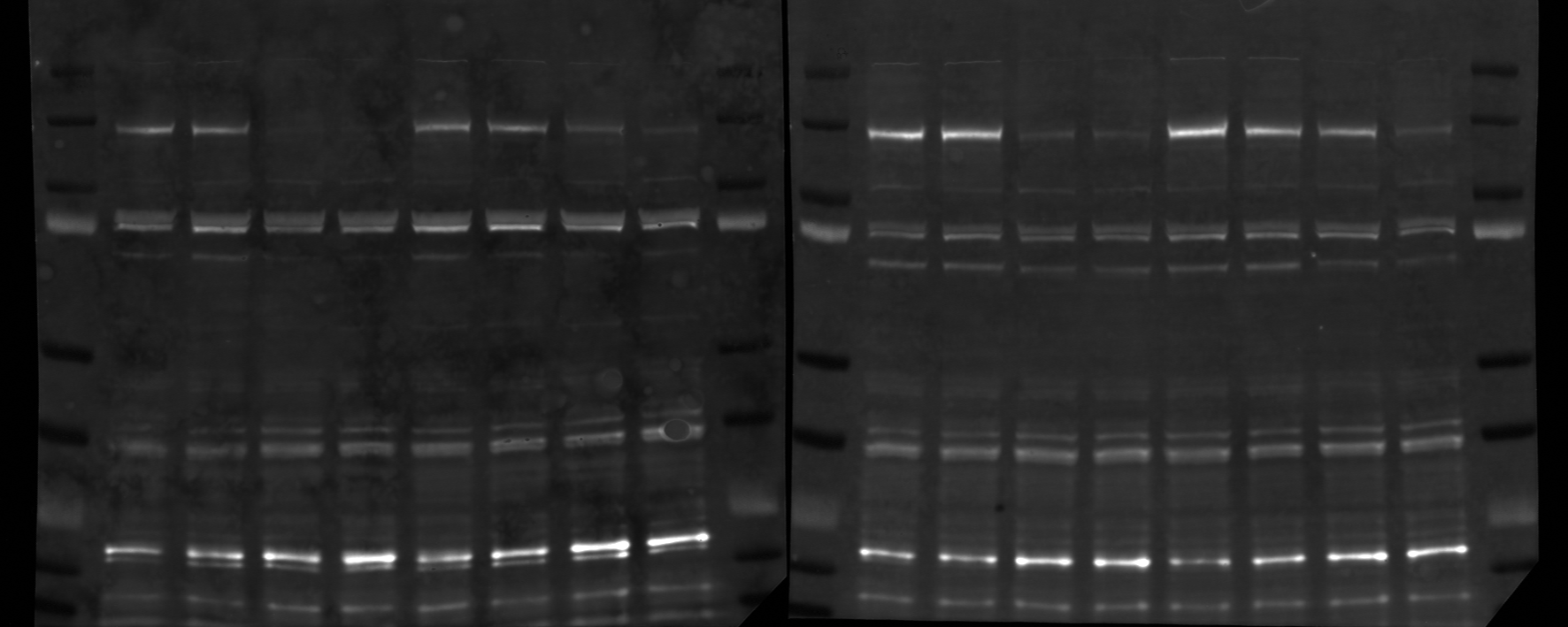

Supplement: Figure 5—source data 2. [file elife-108827-fig5-data2.zip › Figure 5B Gli1 16 bit 800.TIF]

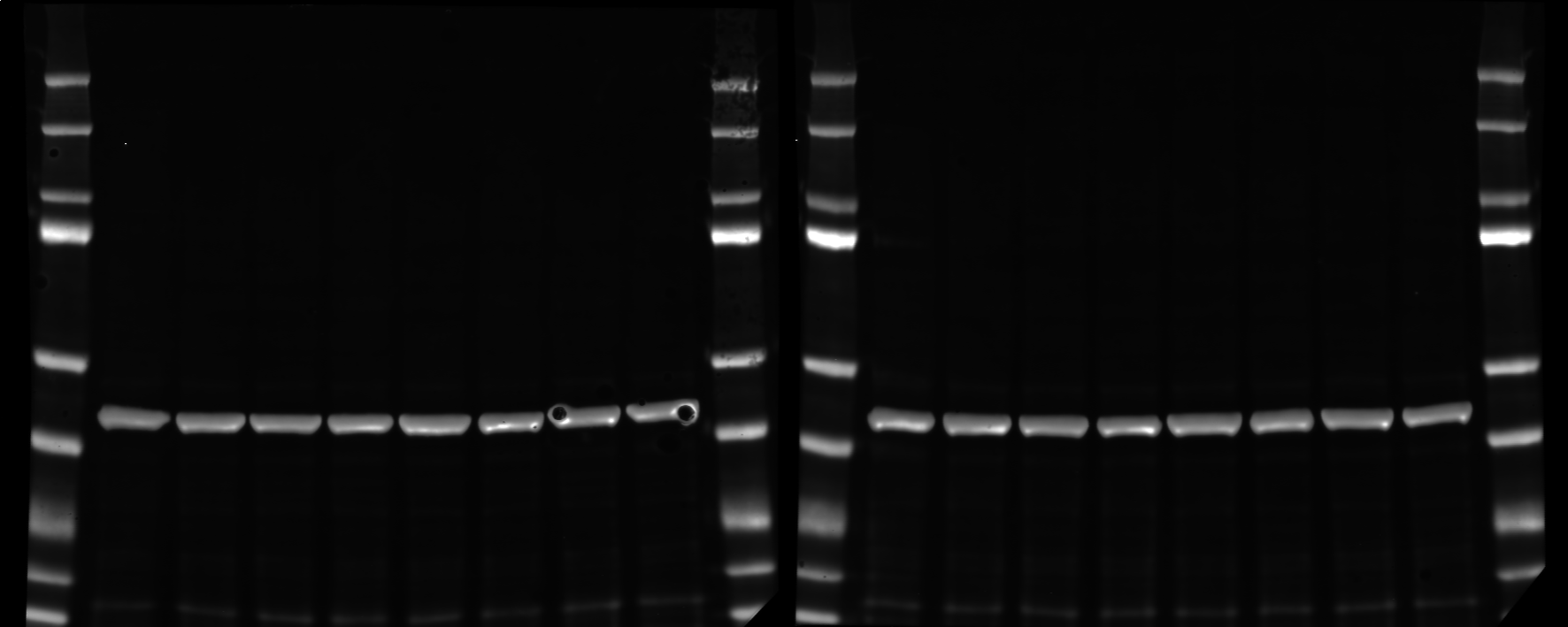

Supplement: Figure 5—source data 2. [file elife-108827-fig5-data2.zip › Figure 5B Gli1 reblot Actin 16 bit 700.TIF]

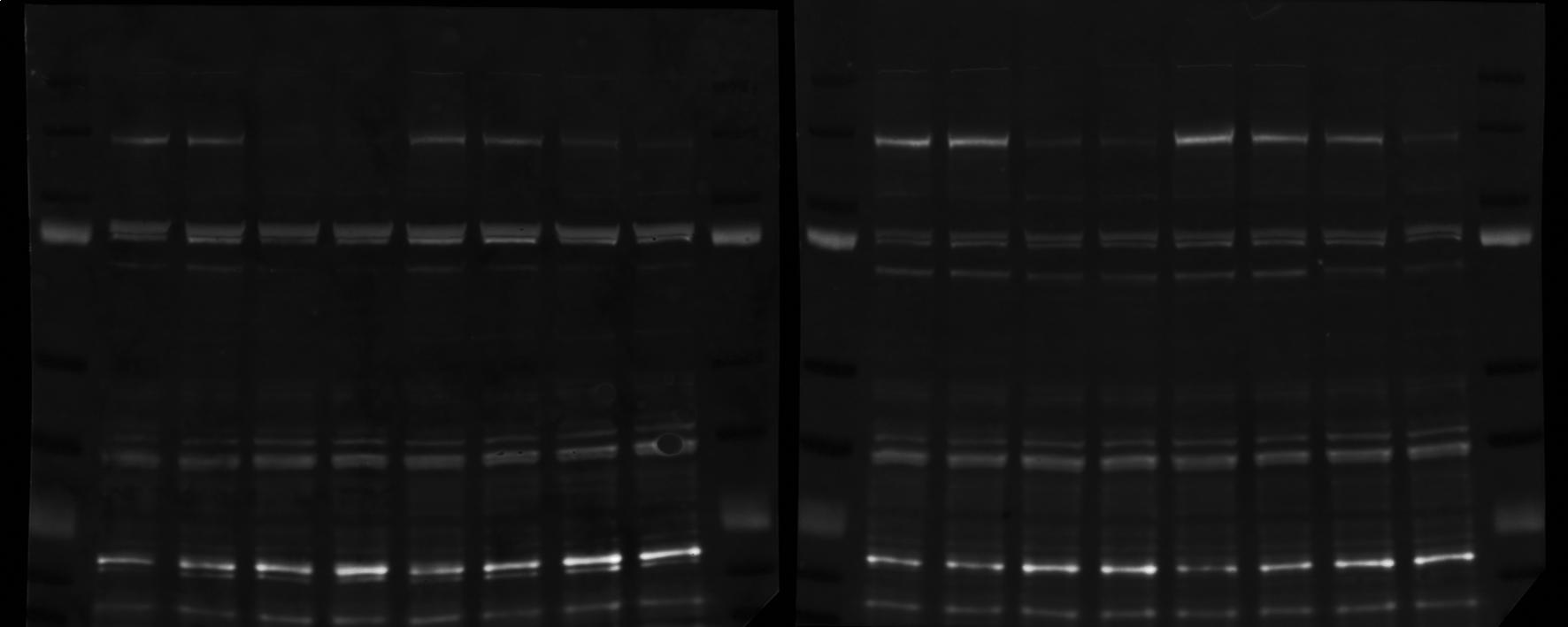

Supplement: Figure 5—source data 2. [file elife-108827-fig5-data2.zip › Figure 5B Gli1 reblot Actin 16 bit 800.TIF]

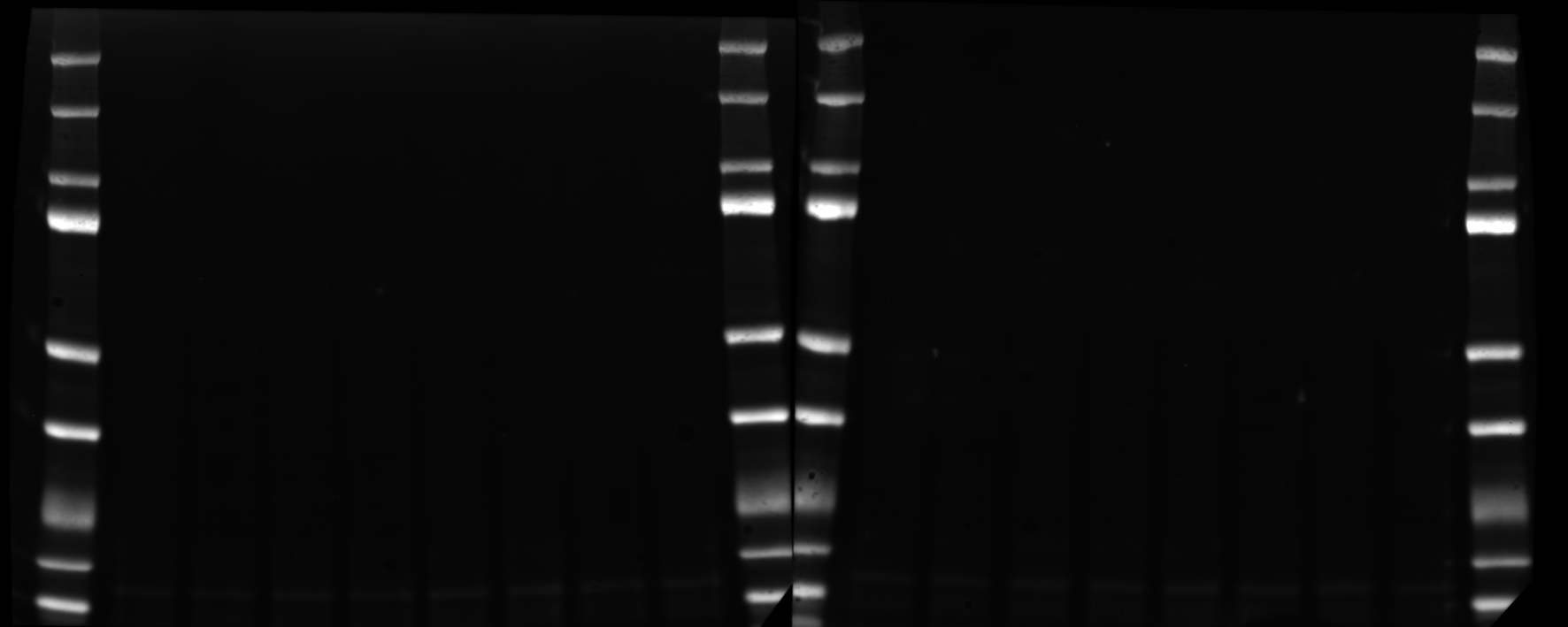

Supplement: Figure 5—source data 2. [file elife-108827-fig5-data2.zip › Figure 5B rep 34 and Figure S9A Gli1 16 bit 700.TIF]

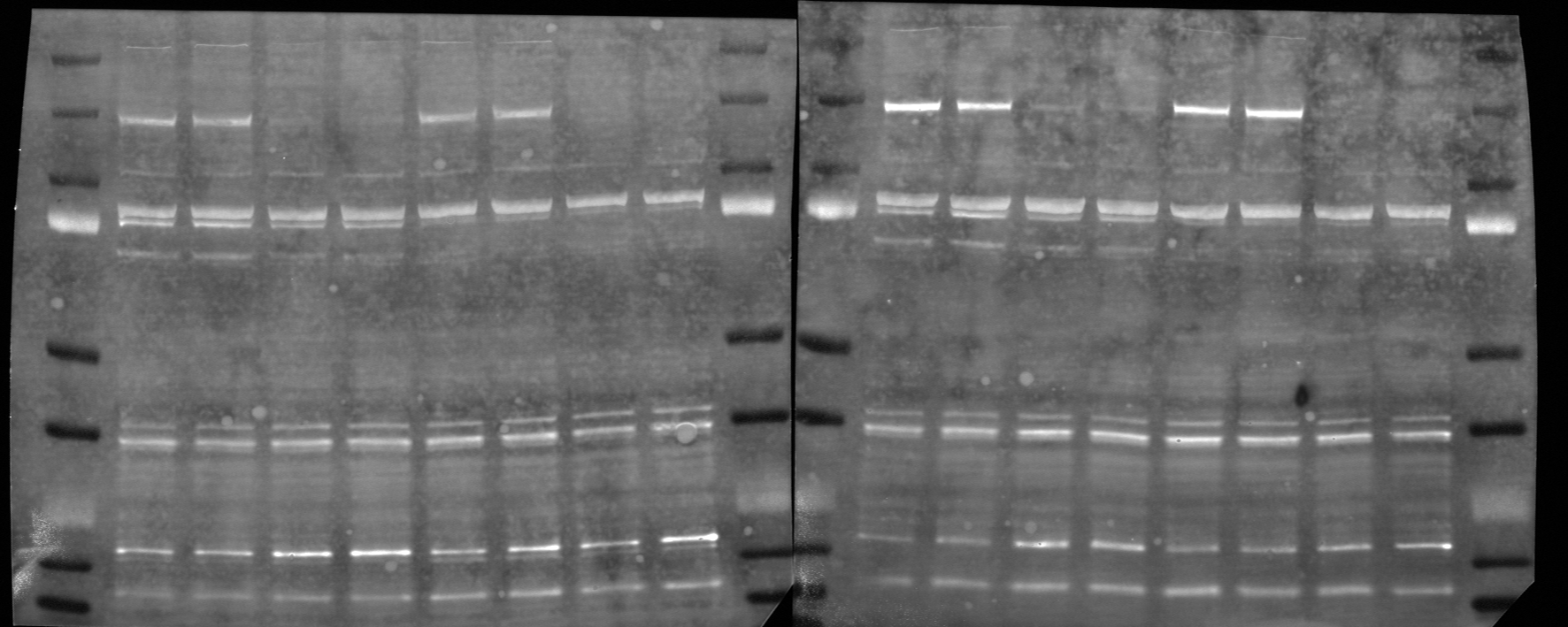

Supplement: Figure 5—source data 2. [file elife-108827-fig5-data2.zip › Figure 5B rep 34 and Figure S9A Gli1 16 bit 800.TIF]

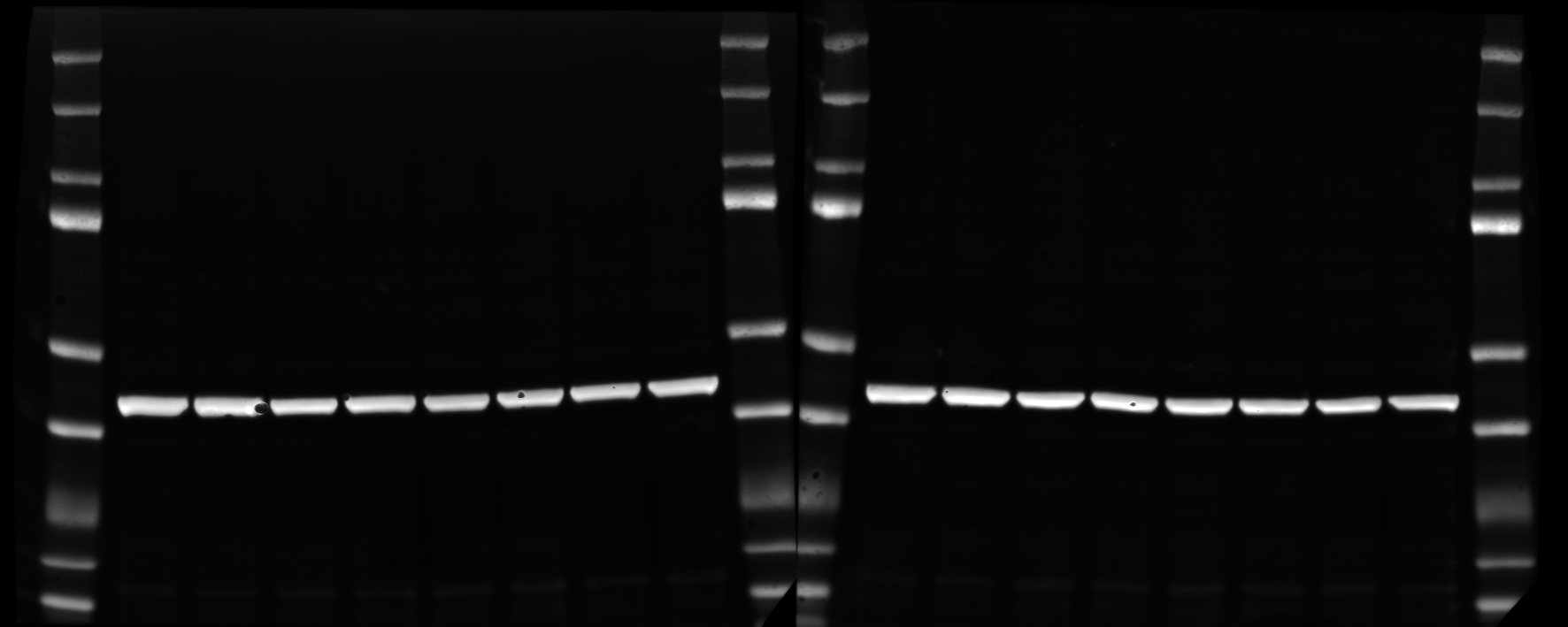

Supplement: Figure 5—source data 2. [file elife-108827-fig5-data2.zip › Figure 5B rep 34 and Figure S9A Gli1 reblot Actin 16 bit 700.TIF]

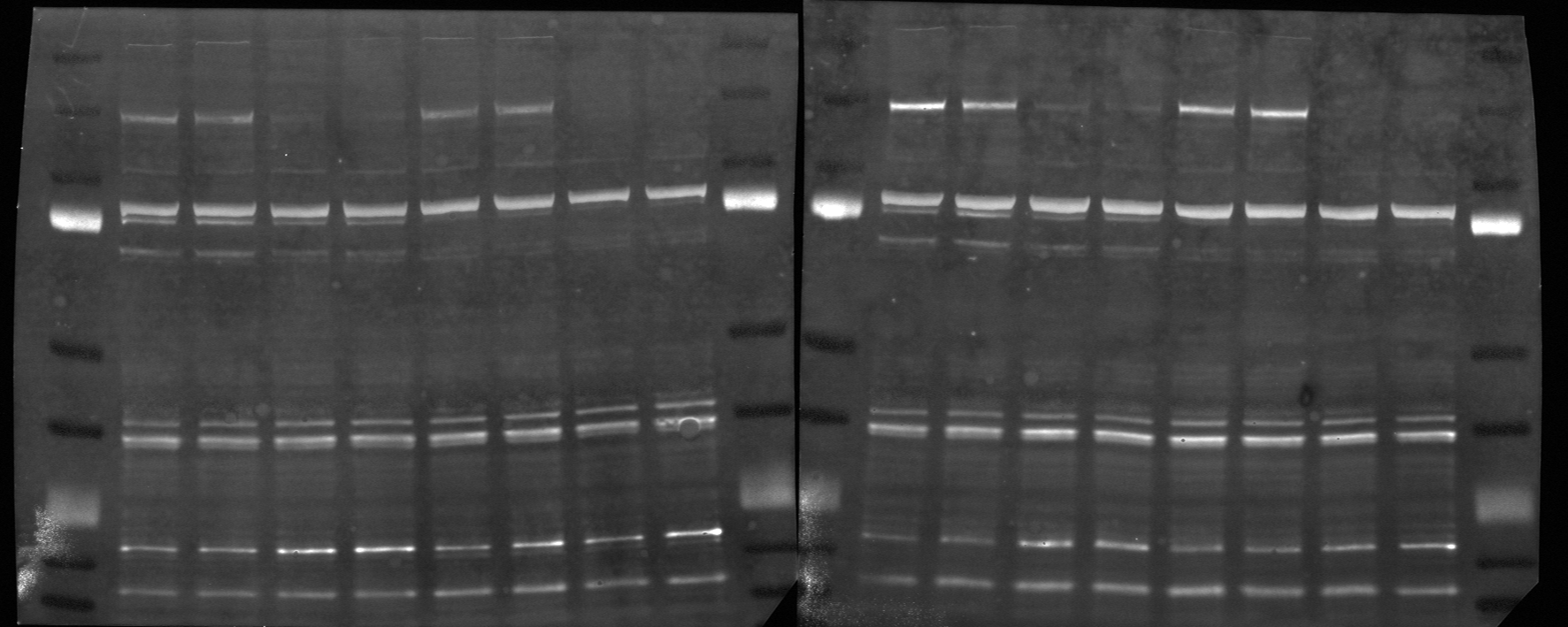

Supplement: Figure 5—source data 2. [file elife-108827-fig5-data2.zip › Figure 5B rep 34 and Figure S9A Gli1 reblot Actin 16 bit 800.TIF]

**Figure 6D:**

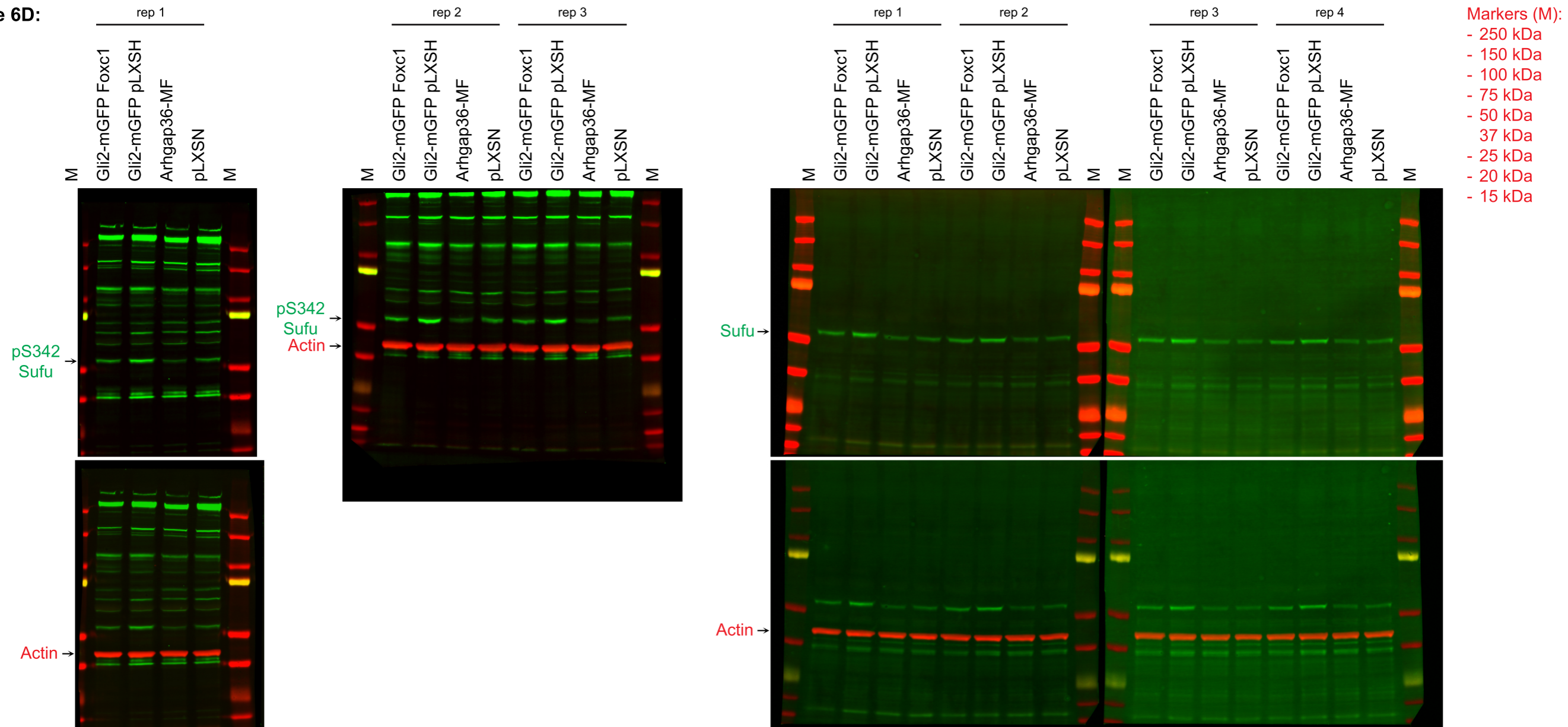

Supplement: Figure 6—source data 1. [file elife-108827-fig6-data1.zip › Figure 6-source data 1.pdf]

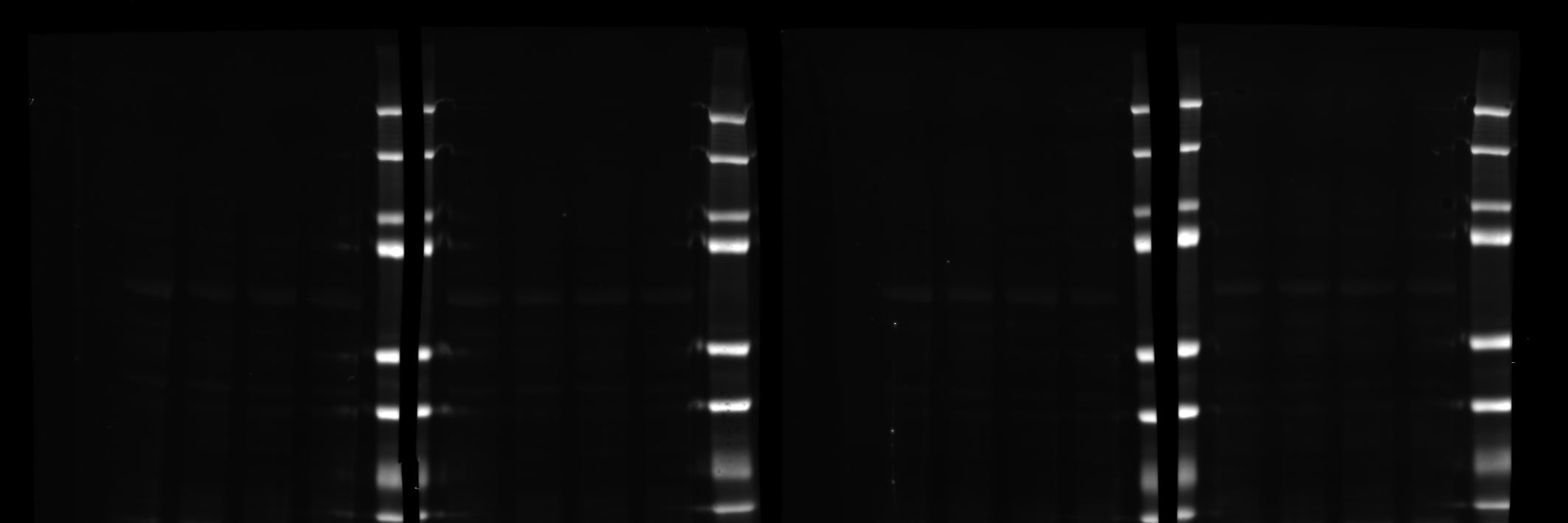

Supplement: Figure 6—source data 2. [file elife-108827-fig6-data2.zip › Figure 6D rep 1 pS342 Sufu 16 bit 700 - second membrane from the left.TIF]

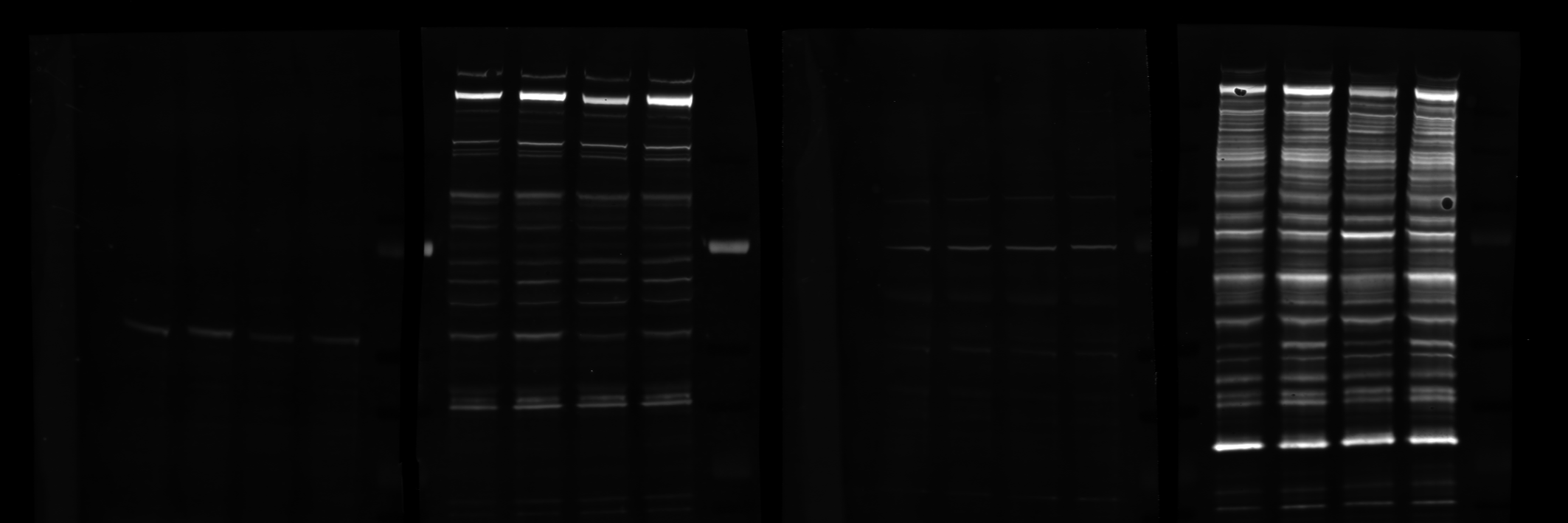

Supplement: Figure 6—source data 2. [file elife-108827-fig6-data2.zip › Figure 6D rep 1 pS342 Sufu 16 bit 800 - second membrane from the left.TIF]

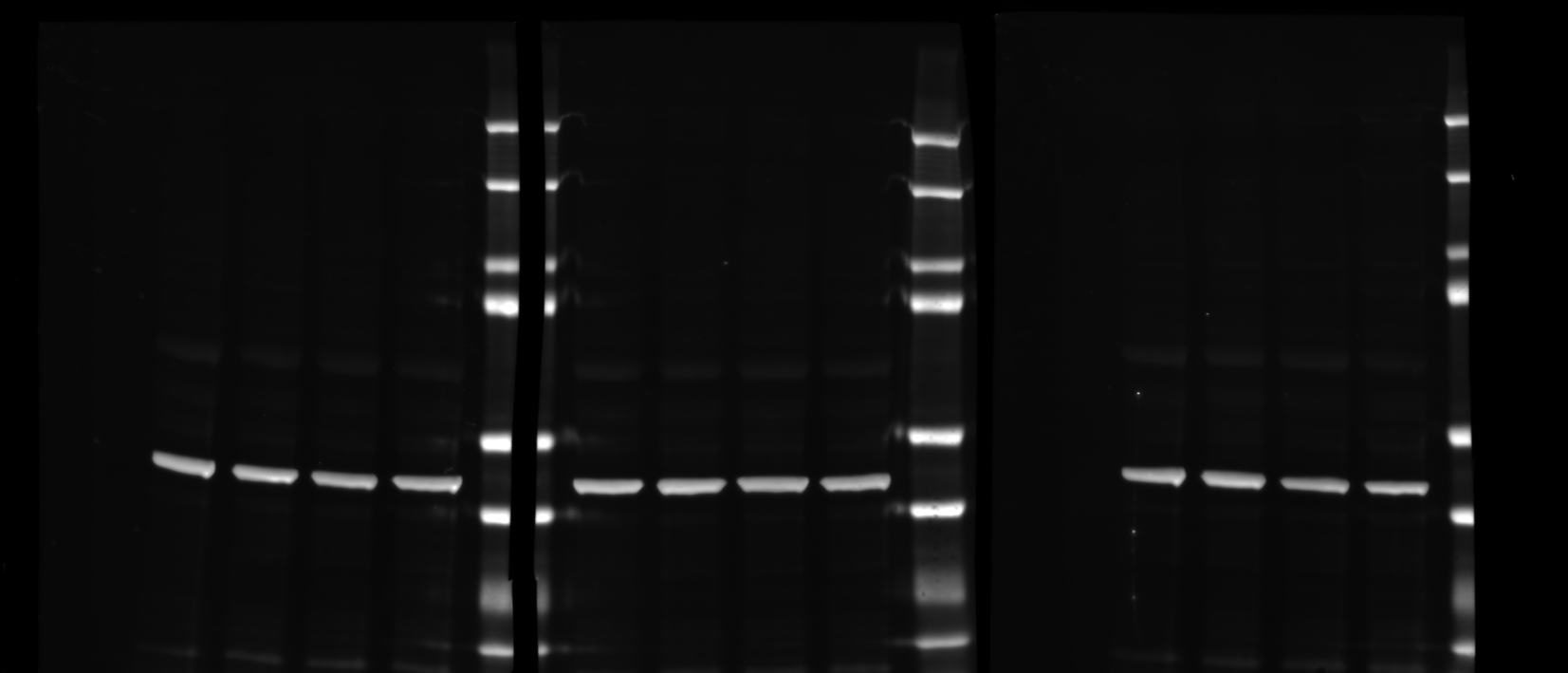

Supplement: Figure 6—source data 2. [file elife-108827-fig6-data2.zip › Figure 6D rep 1 pS342 Sufu reblot Actin 16 bit 700 - second membrane from the left.TIF]

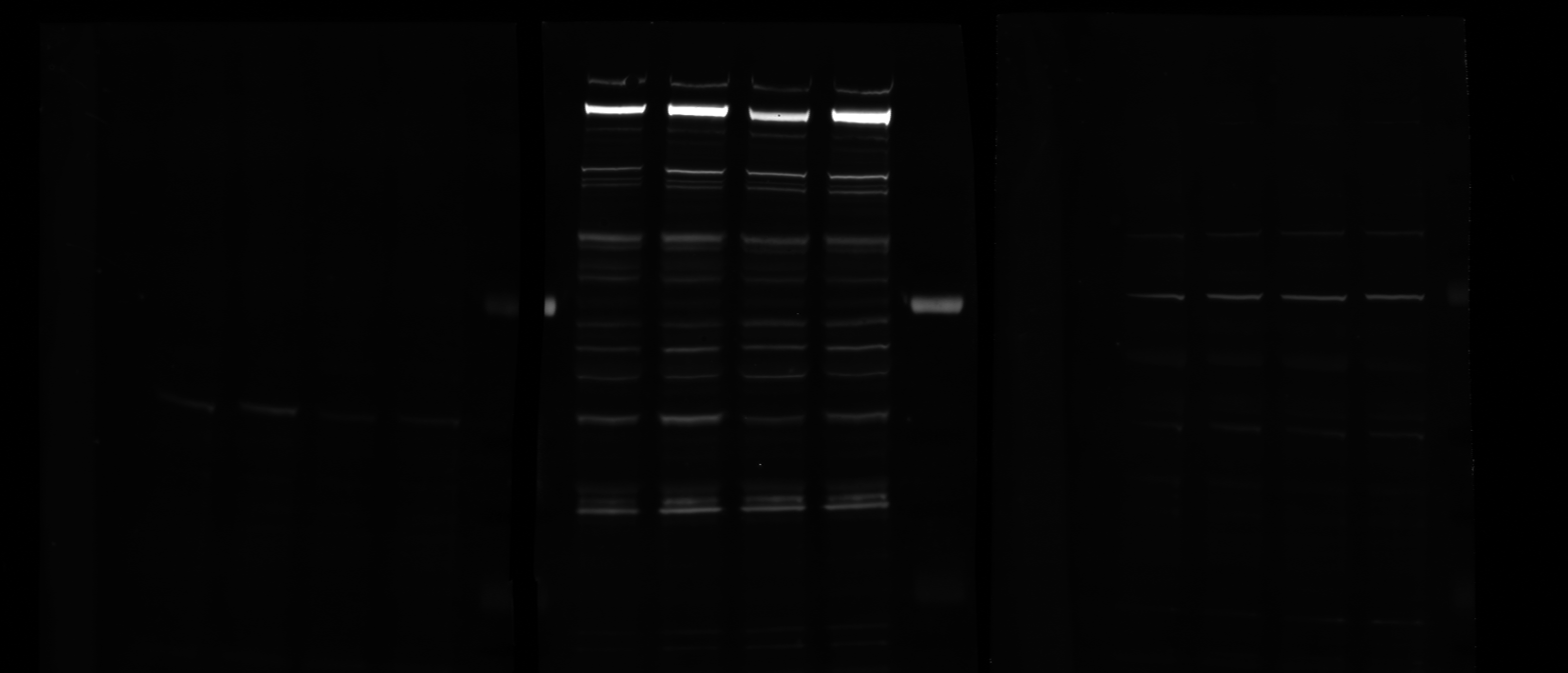

Supplement: Figure 6—source data 2. [file elife-108827-fig6-data2.zip › Figure 6D rep 1 pS342 Sufu reblot Actin 16 bit 800 - second membrane from the left.TIF]

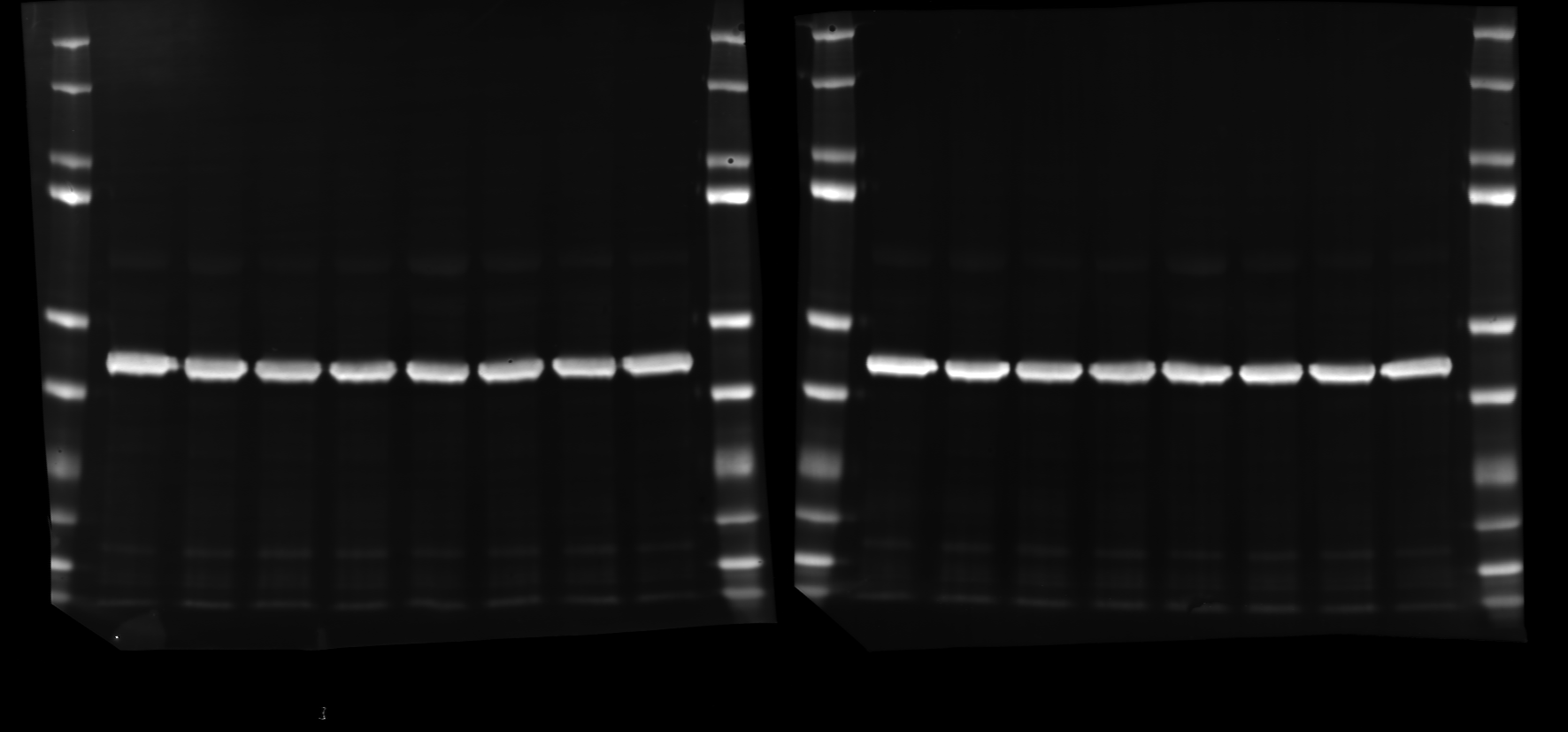

Supplement: Figure 6—source data 2. [file elife-108827-fig6-data2.zip › Figure 6D rep 23 pS342 Sufu Actin 16 bit 700.TIF]

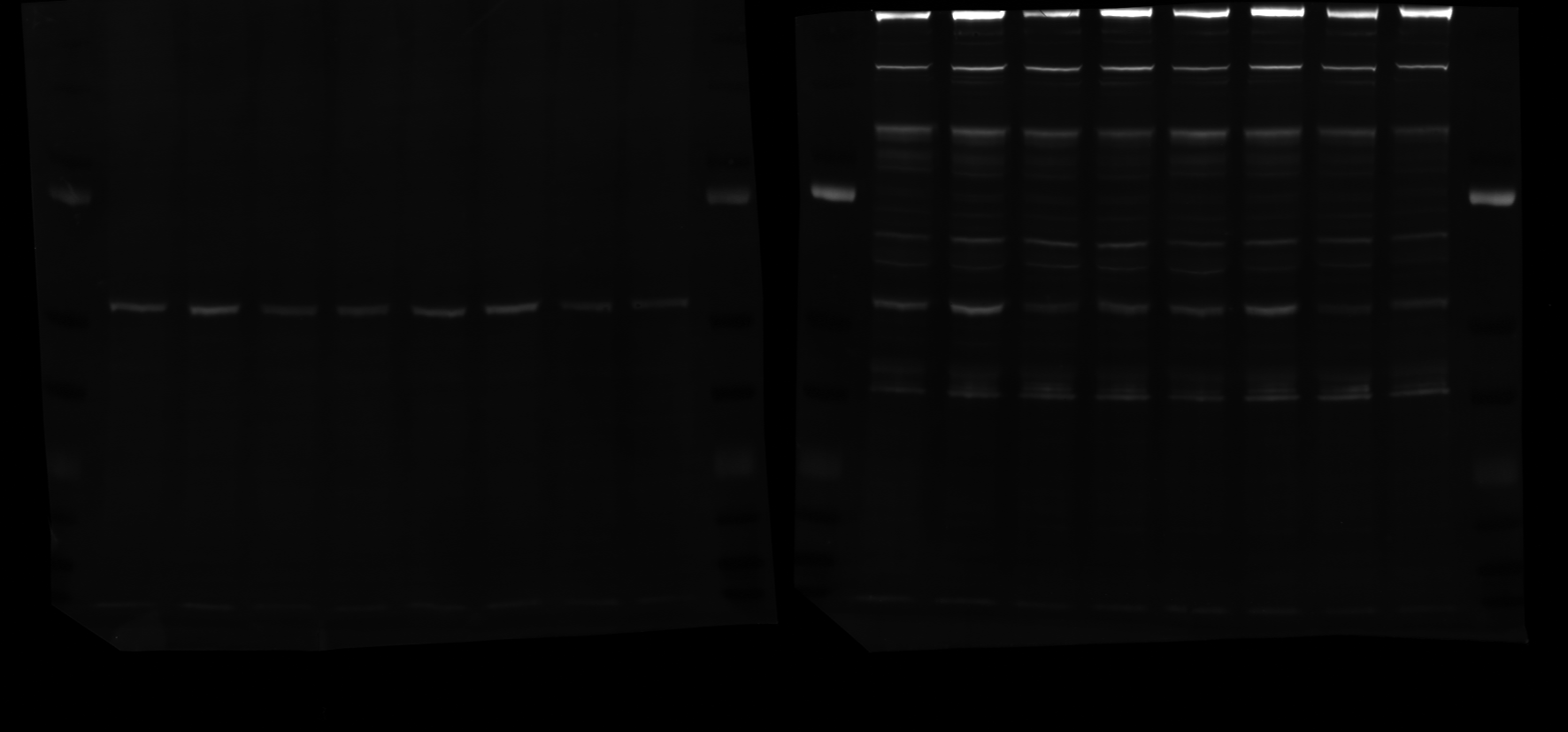

Supplement: Figure 6—source data 2. [file elife-108827-fig6-data2.zip › Figure 6D rep 23 pS342 Sufu Actin 16 bit 800.TIF]

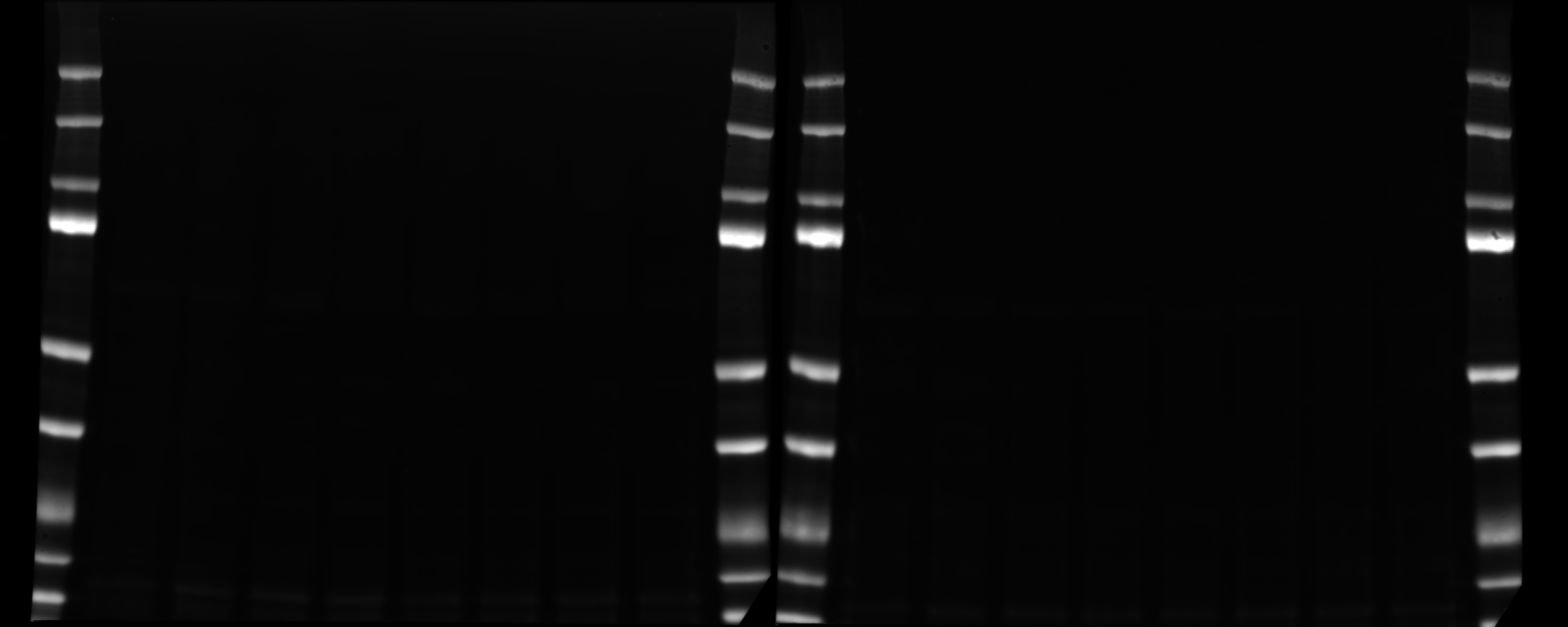

Supplement: Figure 6—source data 2. [file elife-108827-fig6-data2.zip › Figure 6D Sufu 16 bit 700.TIF]

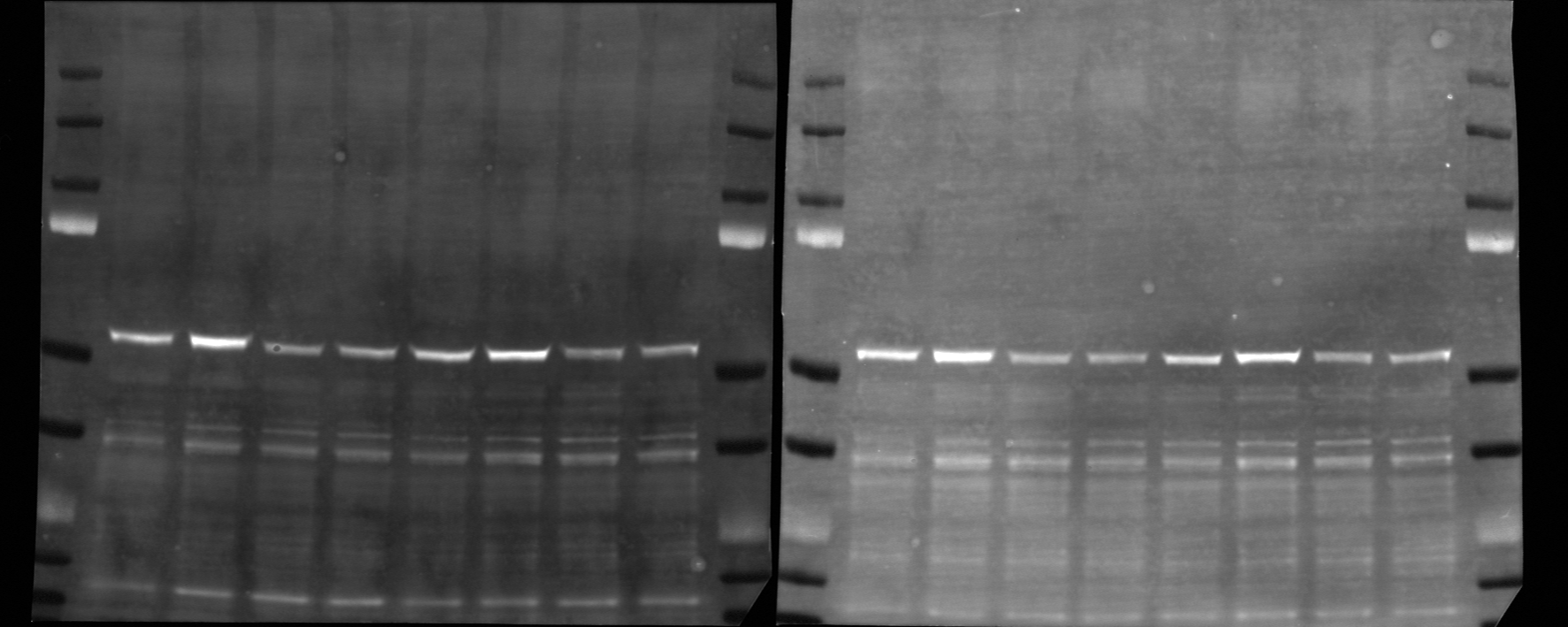

Supplement: Figure 6—source data 2. [file elife-108827-fig6-data2.zip › Figure 6D Sufu 16 bit 800.TIF]

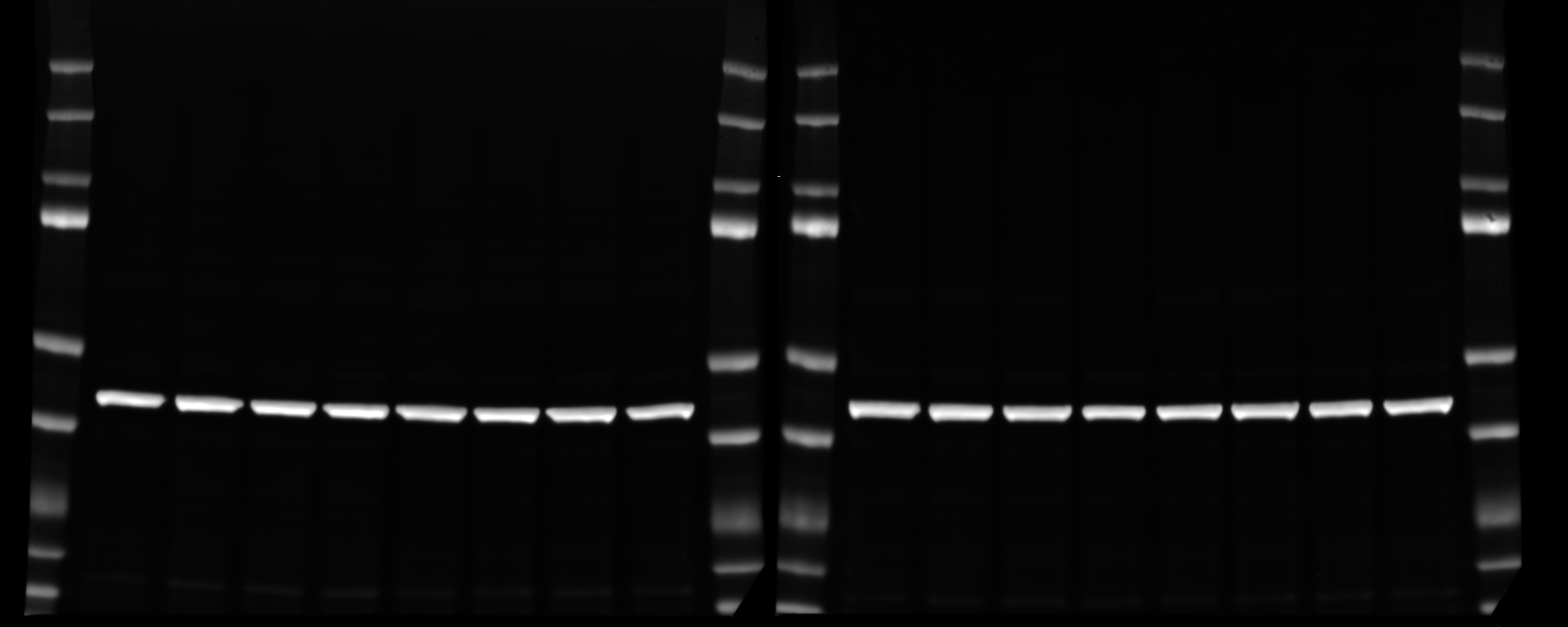

Supplement: Figure 6—source data 2. [file elife-108827-fig6-data2.zip › Figure 6D Sufu reblot Actin 16 bit 700 - second membrane from the left.TIF]

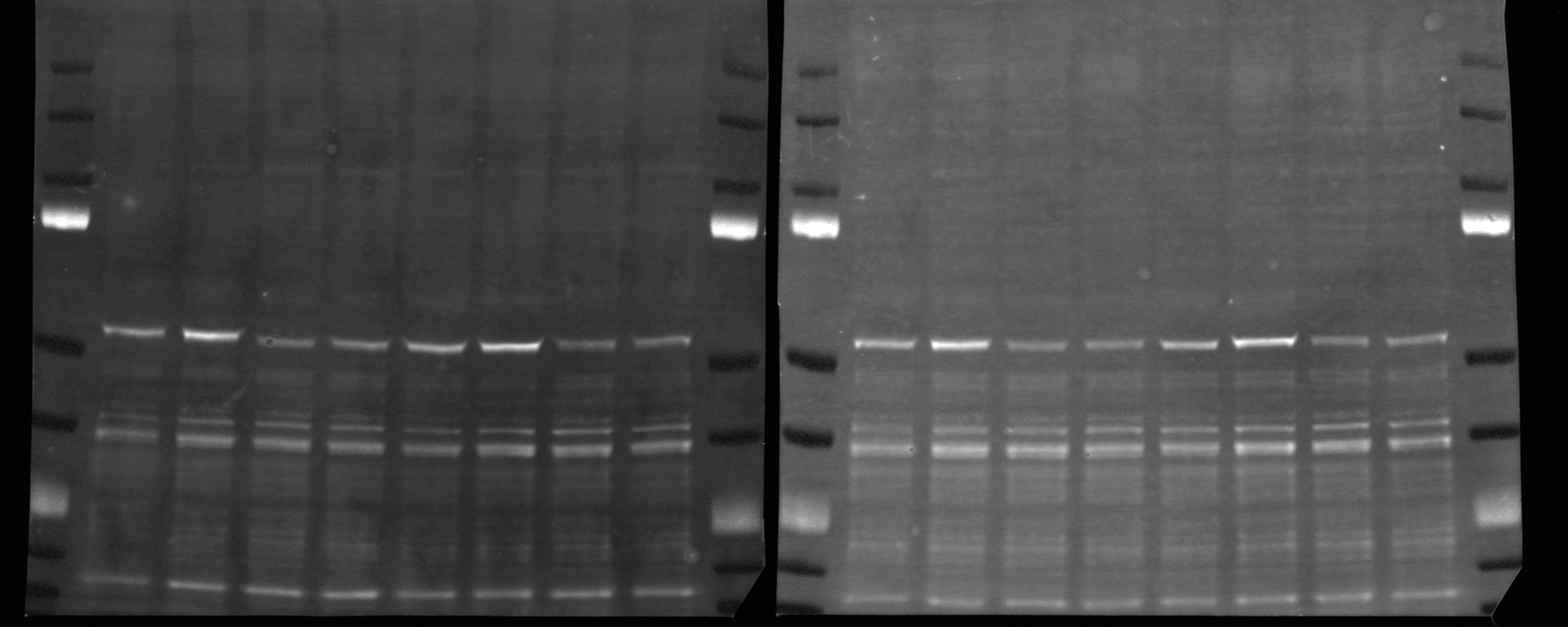

Supplement: Figure 6—source data 2. [file elife-108827-fig6-data2.zip › Figure 6D Sufu reblot Actin 16 bit 800 - second membrane from the left.TIF]

Figure S2C:

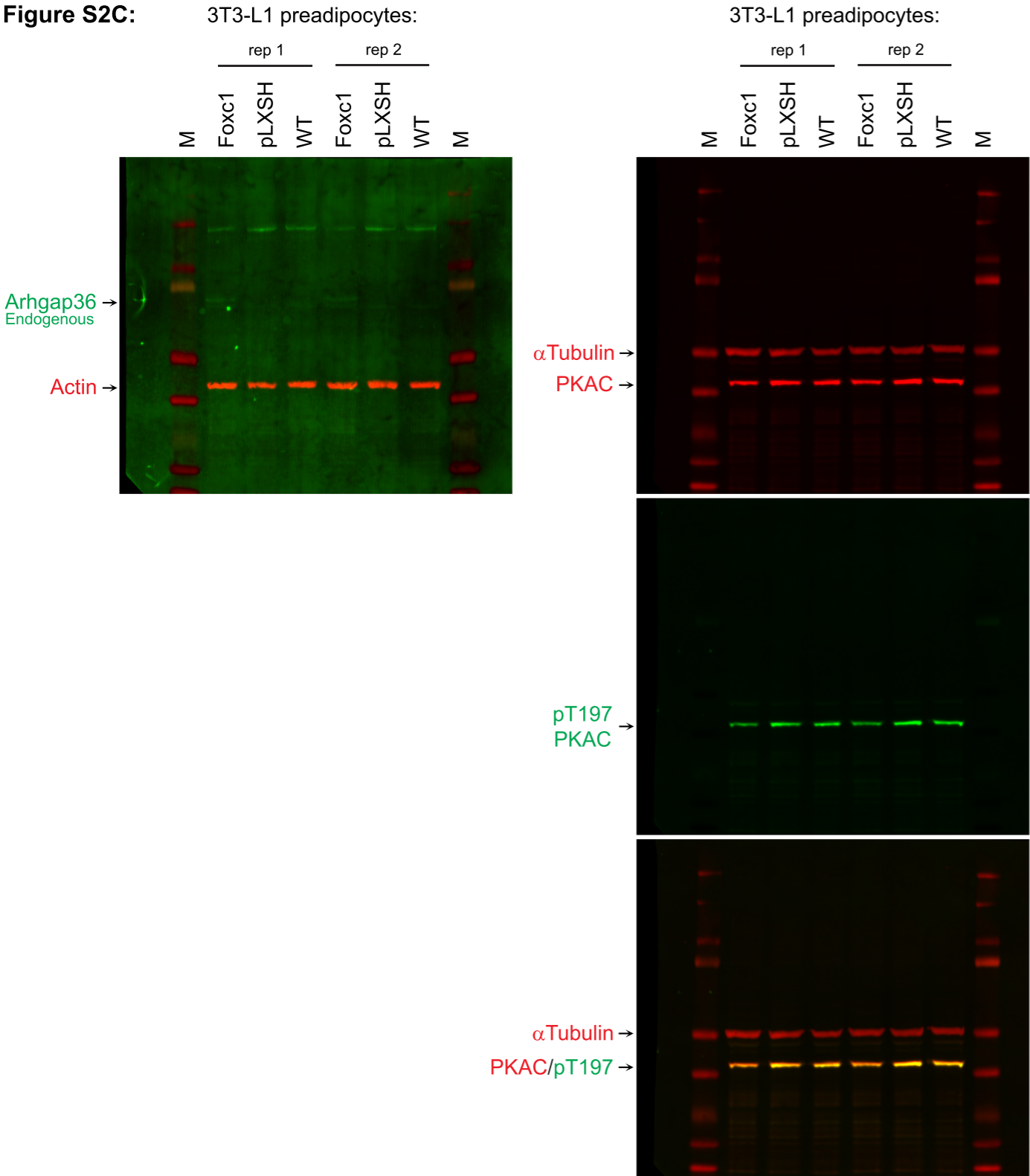

Figure S2D:

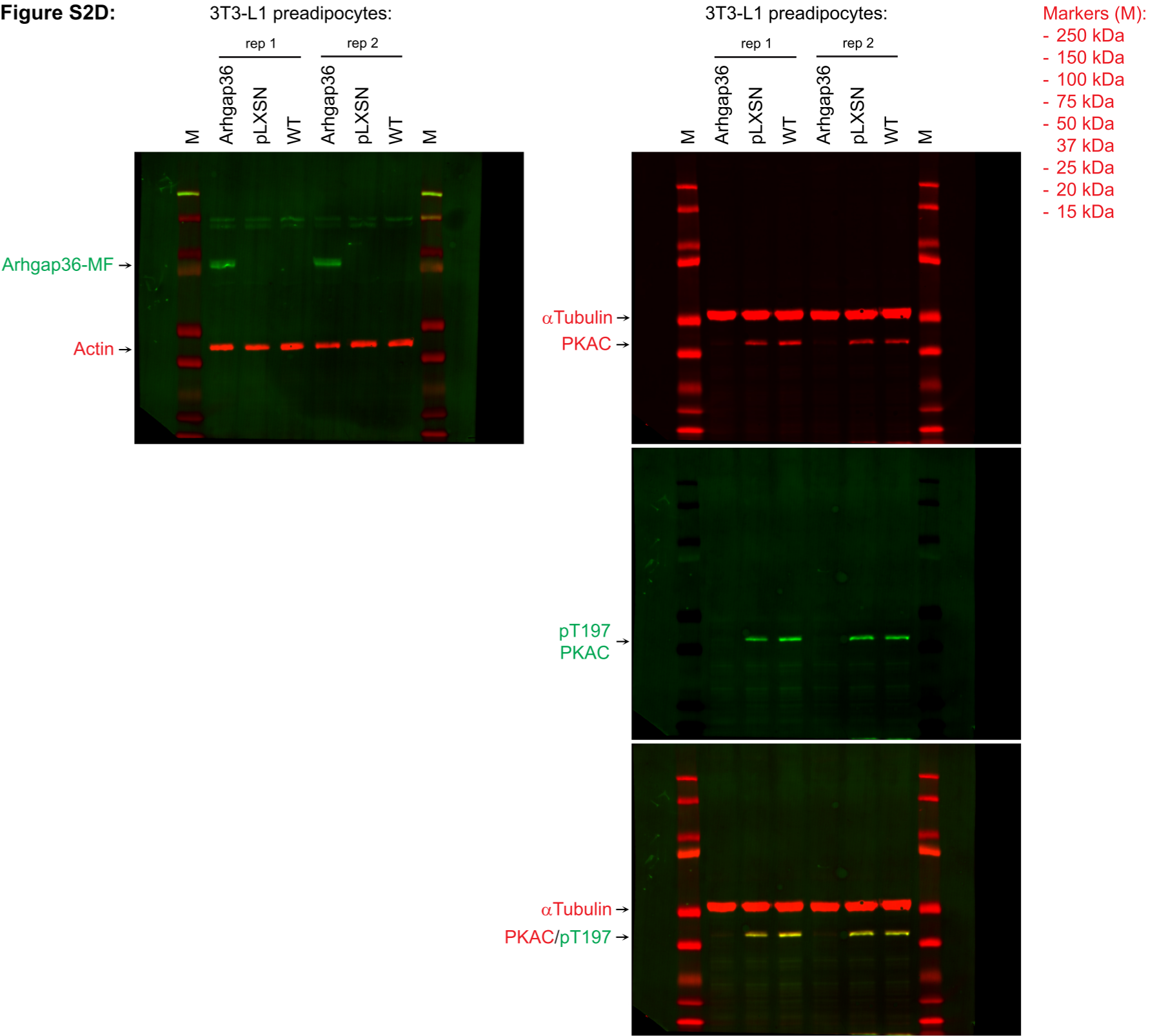

Supplement: Appendix 1—figure 2—source data 1. [file elife-108827-app1-fig2-data1.zip › Figure S2-source data 1.pdf]

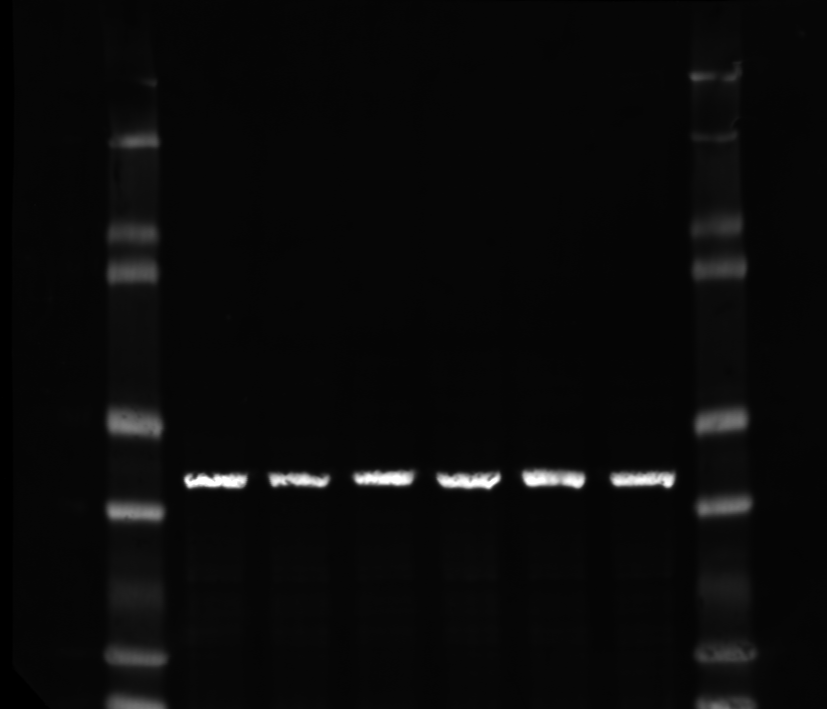

Supplement: Appendix 1—figure 2—source data 2. [file elife-108827-app1-fig2-data2.zip › Figure S2C Arhgap36 Actin 16 bit 700.TIF]

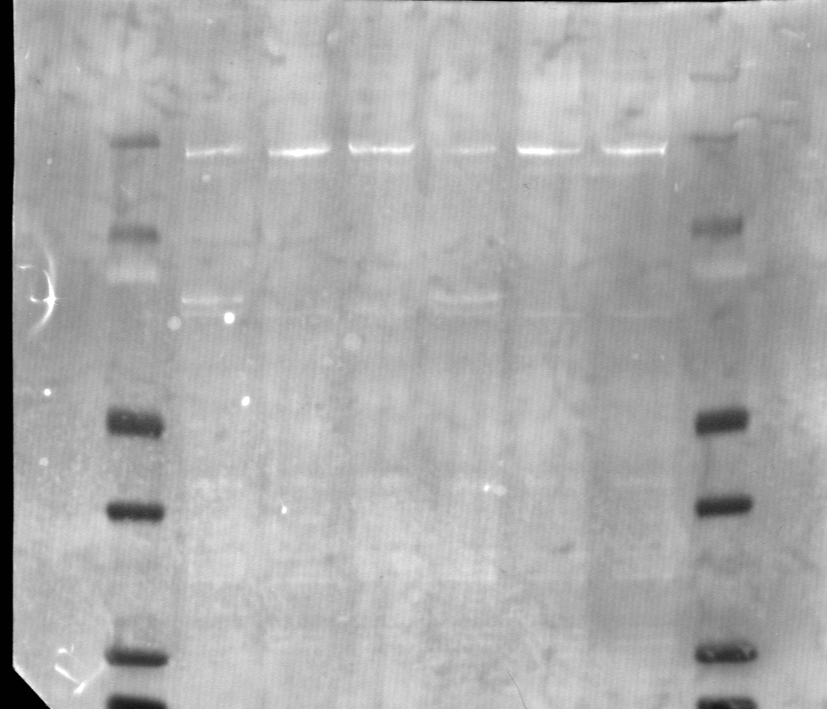

Supplement: Appendix 1—figure 2—source data 2. [file elife-108827-app1-fig2-data2.zip › Figure S2C Arhgap36 Actin 16 bit 800.TIF]

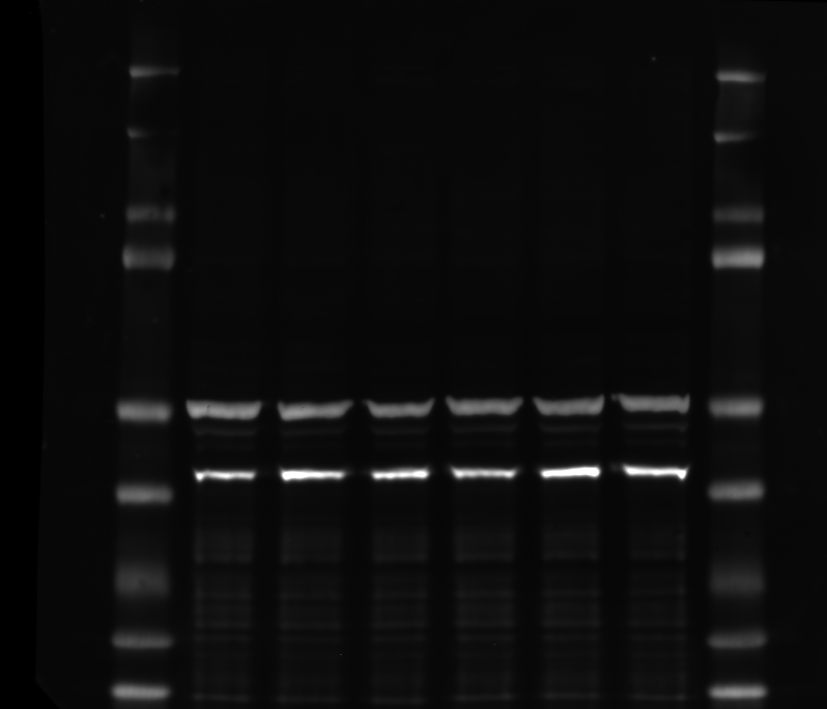

Supplement: Appendix 1—figure 2—source data 2. [file elife-108827-app1-fig2-data2.zip › Figure S2C PKAC pT197 PKAC Tubulin 16 bit 700.TIF]

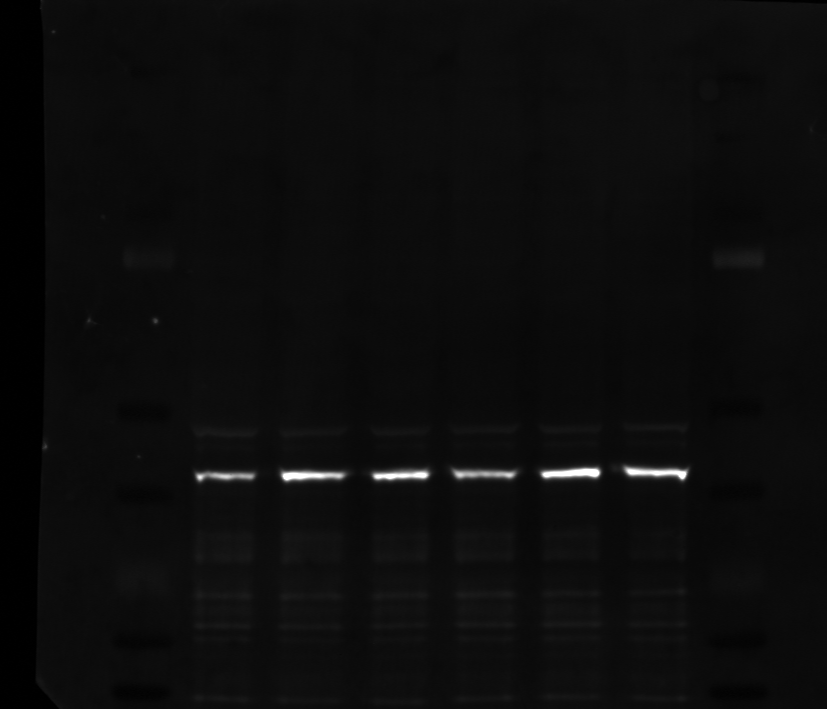

Supplement: Appendix 1—figure 2—source data 2. [file elife-108827-app1-fig2-data2.zip › Figure S2C PKAC pT197 PKAC Tubulin 16 bit 800.TIF]

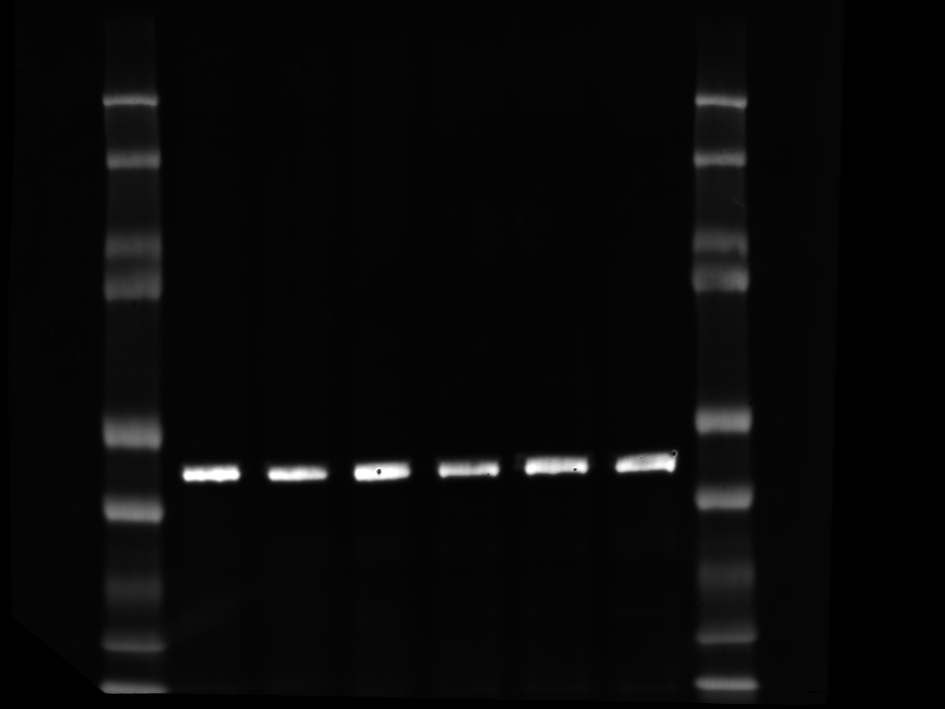

Supplement: Appendix 1—figure 2—source data 2. [file elife-108827-app1-fig2-data2.zip › Figure S2D Arhgap36 Actin 16 bit 700.TIF]

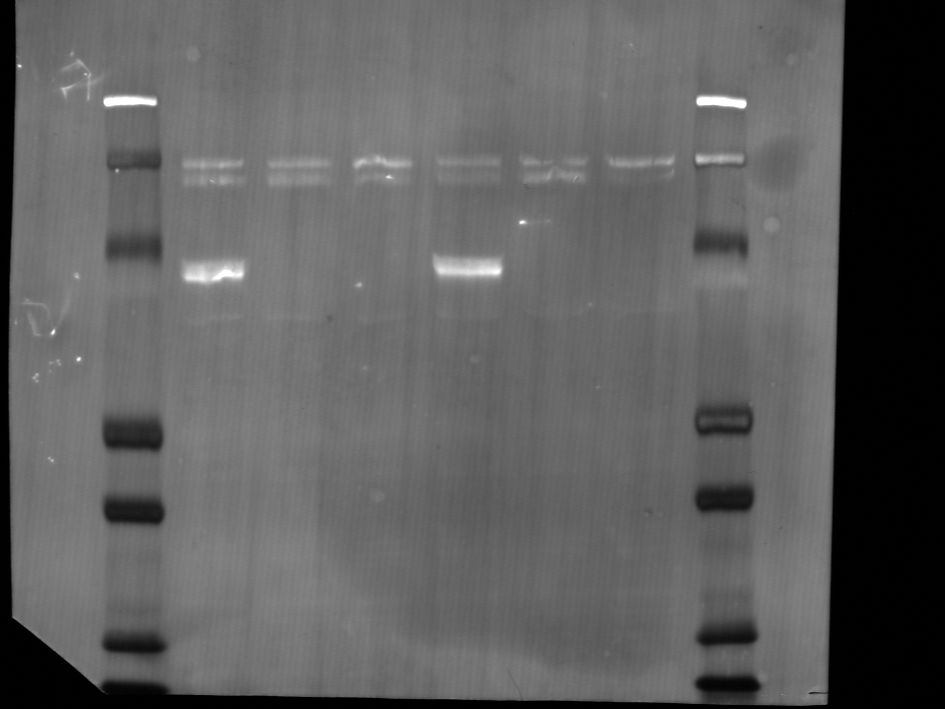

Supplement: Appendix 1—figure 2—source data 2. [file elife-108827-app1-fig2-data2.zip › Figure S2D Arhgap36 Actin 16 bit 800.TIF]

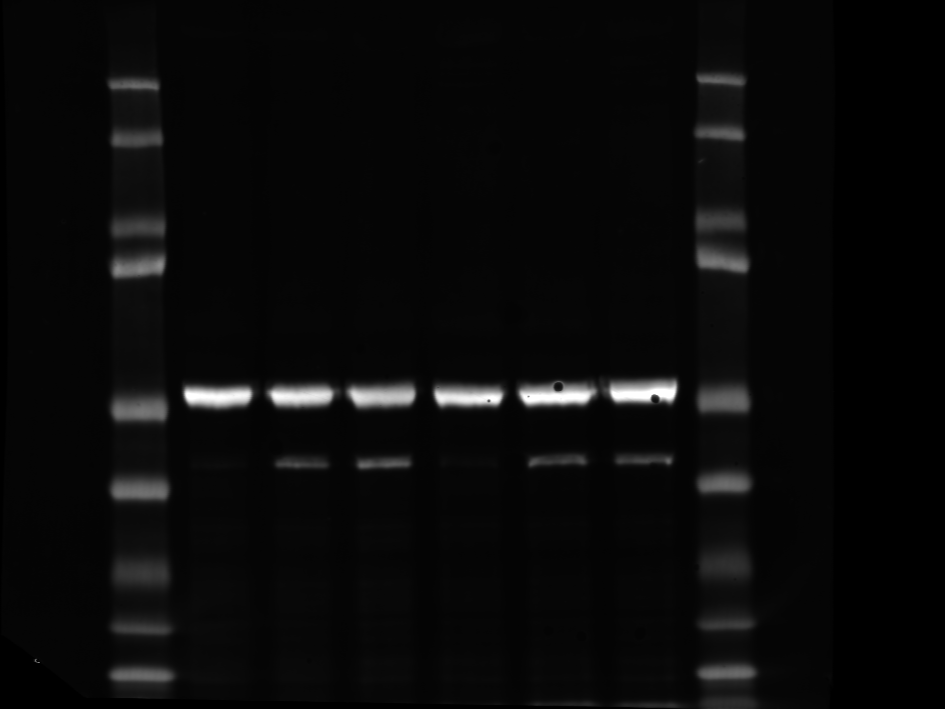

Supplement: Appendix 1—figure 2—source data 2. [file elife-108827-app1-fig2-data2.zip › Figure S2D PKAC pT197 PKAC Tubulin 16 bit 700.TIF]

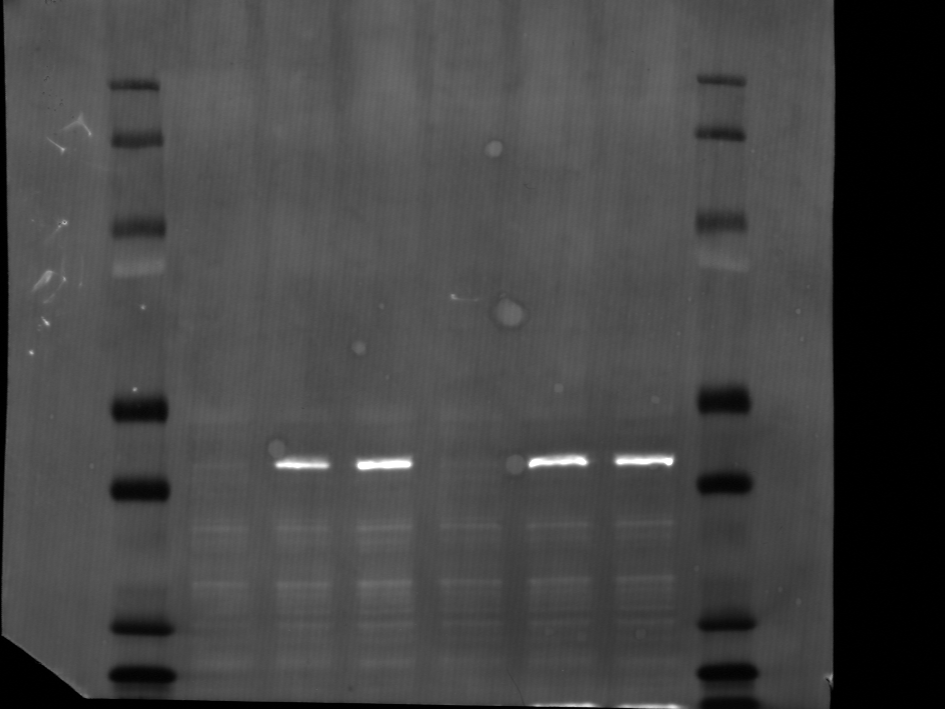

Supplement: Appendix 1—figure 2—source data 2. [file elife-108827-app1-fig2-data2.zip › Figure S2D PKAC pT197 PKAC Tubulin 16 bit 800.TIF]

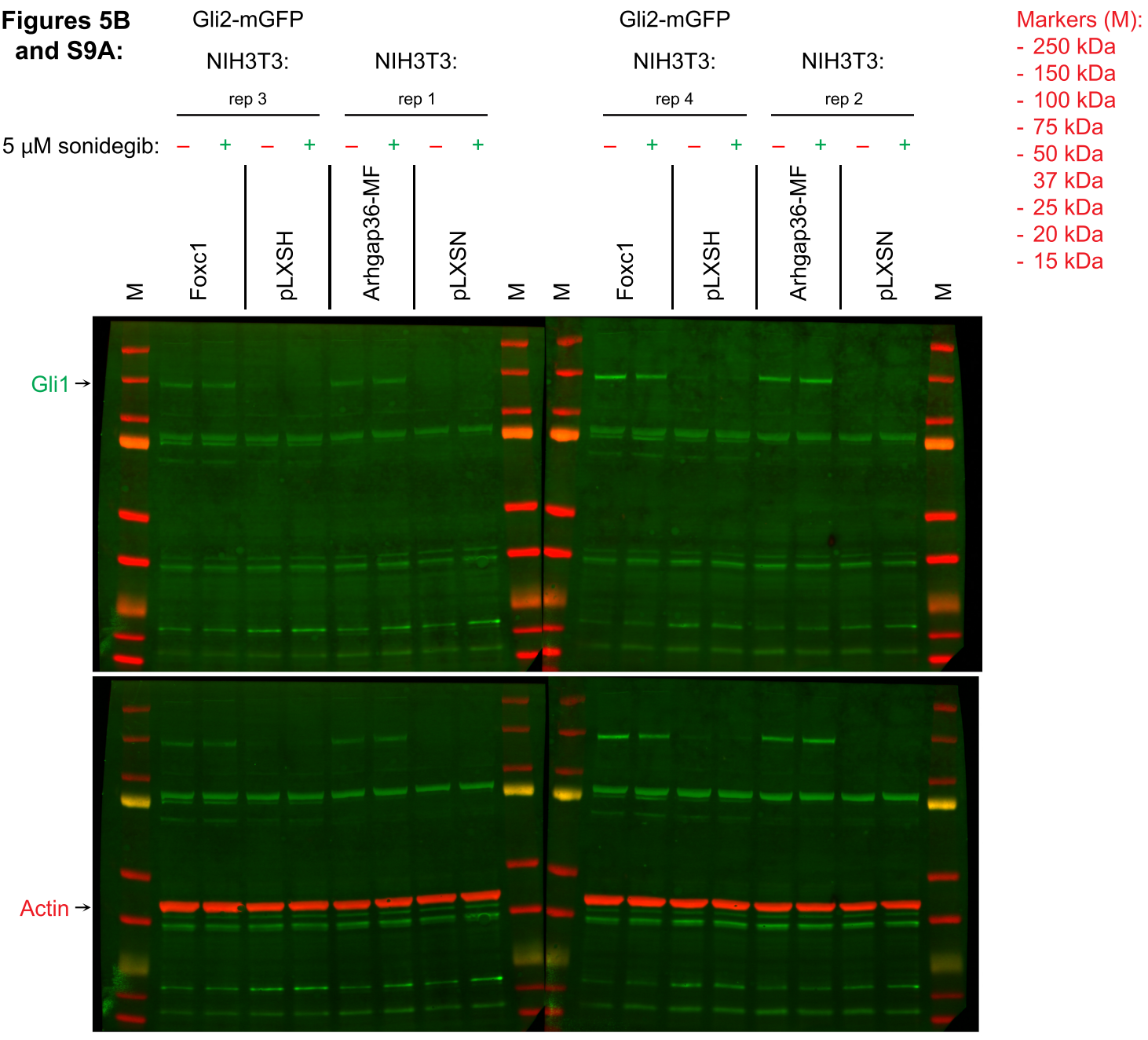

Supplement: Appendix 1—figure 9—source data 1. [file elife-108827-app1-fig9-data1.zip › Figure S9-source data 1.pdf]
