# Supplementary material for: Mechanistic insights into transcriptional regulation of ARHGAP36 expression identify a factor predictive of neuroblastoma survival
Source: eLife. 2026 Jul 6;14:RP108827. doi: 10.7554/eLife.108827 (PMC13336777; doi:10.7554/eLife.108827)
Supplement: Peer review history

# Editor report: eLife Assessment

**DOI:** 10.7554/eLife.108827.3.sa0

**Authors:** Samuel Pleasure

**Affiliations:** University of California, San Francisco United States

**Keywords:** Important, Solid

This **important** study examines the potential role of ARHGAP36 transcriptional regulation by FOXC1 in controlling sonic hedgehog signaling in human neuroblastoma. While there are many **solid** findings that strongly support this signaling pathway, there are some aspects of the study that are underdeveloped, particularly the generalizability in the context of cancer cells.

# Referee report: Reviewer #1 (Public review)

**DOI:** 10.7554/eLife.108827.3.sa1

This thoughtful and thorough mechanistic and functional study reports ARHGAP36 as a direct transcriptional target of FOXC1 which regulates Hedgehog signaling (SUFU, SMO, and GLI family transcription factors) through modulation of PKAC. Clinical outcome data from patients with neuroblastoma, one of the most common extracranial solid malignancies in children, demonstrate that ARHGAP36 expression is associated with improved survival. Although this study largely represents a robust and near-comprehensive set of focused investigations on a novel target of FOXC1 activity, several significant omissions undercut the generalizability of the findings reports.

(1) It is notable that the volcano plot in Fig. 1a does now show evidence of canonical Hedgehog gene regulation even though the subsequent studies in this paper clearly demonstrate that ARHGAP36 regulates Hedgehog signal transduction. Is this because canonical Hedgehog target genes (GLI1, PTCH1, SUFU) simply weren't labeled? Or is there a technical limitation that needs to be clarified? A note about Hedgehog target genes is made in conjunction with Table S1, but the justification or basis of defining these genes as Hedgehog targets is unclear. More broadly, it would be useful to see ontology analyses from these gene expression data to understand FOXC1 target genes more broadly. Ontology analyses are included in a supplementary table, but network visualizations would be much preferred.

(2) Likewise, the ChIP-seq data in Fig. 2 are under-analyzed, focusing only on the ARHGAP36 locus and not more broadly on the FOXC1 gene expression program. This is a missed opportunity that should be remedied with unbiased analyses intersecting differentially expressed FOXC1 peaks with differentially expressed genes from RNA-sequencing data displayed in Fig. 1.

(3) RNA-seq and ChIP-seq data strongly suggest that FOXC1 regulates ARHGAP36 expression, and the authors convincingly identify genomic segments at the ARHGAP36 locus where FOXC1 binds, but they do not test if FOXC1 specifically activates this locus through the creation of a luciferase or similar promoter reporter. Such a reagent and associated experiments would not only strengthen the primary argument of this investigation but could serve as a valuable resource for the community of scientists investigating FOXC1, ARHGAP36, the Hedgehog pathway, and related biological processes. CRISPRi targeting of the identified regions of the ARHGAP locus is a useful step in the right direction, but these experiments are not done in a way to demonstrate FOXC1 dependency.

(4) It would be useful to see individual fluorescence channels in association with images in Fig. 3b.

(5) Perhaps the most significant limitation of this study is the omission of in vivo data, a shortcoming the authors partly mitigate through the incorporation of clinical outcome data from pediatric neuroblastoma patients in the context of ARHGAP36 expression. The authors also mention that high levels of ARHGAP36 expression were also detected in "specific CNS, breast, lung, and neuroendocrine tumors," but do not provide clinical outcome data for these cohorts. Such analyses would be useful to understand the generalizability of their findings across different cancer types. More broadly, how were high, medium, and low levels of ARHGAP36 expression identified? "Terciles" are mentioned, but such an approach is not experimentally rigorous and RPA or related approaches (nested rank statistics, etc) are recommended to find optimal cutpoints for ARHGAP36 expression in the context of neuroblastoma, "specific CNS, breast, lung, and neuroendocrine" tumor outcomes.

Comments on revisions:

I am underwhelmed by this revision, for which I recommended more visualizations of already-generated bioinformatic data that the authors have not provided. Some attempts were made (e.g. network analysis), but other suggestions for improvement were not incorporated (e.g. more comprehensive ChIP-seq analysis). Beyond these relatively straightforward missed opportunities for improvement, there remains a lack of in vivo data and the clinical relevance of these findings are unclear due to potential sources of bias in the data sets analyzed.

# Referee report: Reviewer #2 (Public review)

**DOI:** 10.7554/eLife.108827.3.sa2

FOXC1 is a transcription factor essential for the development of neural crest-derived tissues and has been identified as a key biomarker in various cancers. However, the molecular mechanisms underlying its function remain poorly understood. In this study, the authors used RNA-seq, ChIP-seq, and FOXC1-overexpressing cell models to show that FOXC1 directly activates transcription of ARHGAP36 by binding to specific cis-regulatory elements. Elevated expression of FOXC1 or ARHGAP36 was found to enhance Hedgehog (Hh) signaling and suppress PKA activity. Notably, overexpression of either gene also conferred resistance to Smoothened (SMO) inhibitors, indicating ligand-independent activation of Hh signaling. Analysis of public gene expression datasets further revealed that ARHGAP36 expression correlates with improved 5-year overall survival in neuroblastoma patients. Together, these findings uncover a novel FOXC1-ARHGAP36 regulatory axis that modulates Hh and PKA signaling, offering new insights into both normal development and cancer progression.

Main strengths of the study are:

(1) Identification of a novel signaling pathway involving FOXC1 and ARHGAP36, which may play a critical role in both normal development and cancer biology. (2) Mechanistic investigation using RNA-seq, ChIP-seq, and functional assays to elucidate how FOXC1 regulates ARHGAP36 and how this axis modulates Hh signaling. (3) Clinical relevance demonstrated through analysis of neuroblastoma patient datasets, linking ARHGAP36 expression to improved 5-year overall survival.

Comments on revisions:

Consider adding subsection titles to the Results section to better organize the findings and improve readability.

The authors may consider adding a statement in paragraph 4 of the Results section or in the Discussion noting that ARHGAP36 has been reported to inhibit PKAC activity and promote PKAC degradation.

# Referee report: Reviewer #3 (Public review)

**DOI:** 10.7554/eLife.108827.3.sa3

Summary:

The focus of the research is to understand how transcription factors with high expression in neural crest cell derived cancers (e.g., neuroblastoma) and roles in neural crest cell development function to promote malignancy. The focus is on the transcription factor FOXC1 and using murine cell culture, gain- and loss of function approaches and ChIP profiling, among other techniques, to place PKC inhibitor ARHGAP36 mechanistically between FOXC1 and another pathway associated with malignancy, Sonic Hedgehog (SHH).

Strengths:

Major strengths are the mechanistic approaches to identify FOXC1 direct targets, definitively showing that FOXC1 transcriptional regulation of ARHGAP36 leads to dysregulation of SHH signaling downstream of ARHGAP36 inhibition of PKC. Starting from a screen of Foxc1 OE to get to ARHGAP36 and then using genetic and pharmacological manipulation to work through the mechanism is very well done. There is data that will be of use to others studying FOXC1 in mesenchymal cell types, in particular the FOXC1 ChIP-seq.

Weaknesses:

Work is almost all performed in NIH3T3 or similar cells (mouse cells, not patient or mouse-derived cancer cells) so the link to neuroblastoma that forms the major motivation of the work is not clear. The authors look at ARHGAP36 levels in association the neuroblastoma patient survival however the finding, though interesting and quite compelling, is misaligned with what the literature shows about FOXC1 and SHH, their high expression is associated with increased malignancy (also maybe worse outcomes?). Therefore, ARHGAP36 expression may be more complicated in a tumor cell or may be unrelated to FOXC1 or SHH, leaving one to wonder what the work in NIH3T3 cells, though well done, is telling us about the mechanisms of FOXC1 as an oncogene in neuroblastoma cells or in any type of cancer cell. Does it really function as a SHH activator to drive tumor growth? The 'oncogenic relevance' and 'contribution to malignancy' claimed in the last paragraph of the introduction is currently weakly supported with the data as presented. This could be improved with studying some of these mechanisms in patient-derived neuroblastoma cells with high FOXC1 expression. Does inhibiting FOXC1 change SHH and ARHGAP36 and have any effect on cell proliferation or migration? Alternatively, does OE of FOXC1 in NIH3T3 cells increase their migration or stimulate proliferation in some way and is this dependent on ARHGAP36 or SHH? Application of their mechanistic approaches in cancer cells or looking for hallmarks of cancer phenotypes with FOXC1 OE (and dependent on SHH or ARHGAP36) could help to make a link with cellular phenotypes of malignant cells.

In the revised manuscript, the authors did not add studies in any malignant cell type (mouse or human, neuroblastoma or other) with Foxc1 overexpression to test if the mechanisms they identify in the mouse fibroblasts is present in cancer cells nor if this relates to cellular phenotypes of malignancy (migration or proliferation). Therefore strengths and weaknesses identified by this reviewer in the prior version are the same.

# Author comment: Author response

**DOI:** 10.7554/eLife.108827.3.sa4

**Authors:** Serhiy Havrylov, Armin Gamper, Ordan J Lehmann

**Affiliations:** University of Alberta Edmonton Canada; University of Alberta Edmonton Canada; University of Alberta Edmonton Canada

The following is the authors’ response to the original reviews.

**Public Reviews:**

**Reviewer #1 (Public review):**

This thoughtful and thorough mechanistic and functional study reports ARHGAP36 as a direct transcriptional target of FOXC1…… Although this study largely represents a robust and near-comprehensive set of focused investigations on a novel target of FOXC1 activity, several significant omissions undercut the generalizability of the findings reported.

(1) It is notable that the volcano plot in Figure 1a does now show evidence of canonical Hedgehog gene regulation, even though the subsequent studies in this paper clearly demonstrate that ARHGAP36 regulates Hedgehog signal transduction. Is this because canonical Hedgehog target genes (GLI1, PTCH1, SUFU) simply weren't labeled? Or is there a technical limitation that needs to be clarified? A note about Hedgehog target genes is made in conjunction with Table S1, but the justification or basis of defining these genes as Hedgehog targets is unclear. More broadly, it would be useful to see ontology analyses from these gene expression data to understand FOXC1 target genes more broadly. Ontology analyses are included in a supplementary table, but network visualizations would be much preferred.

Space constraints precluded labelling the Volcano plot with all 285 significantly differentially expressed genes. So rather than just Hedgehog pathway members, the most dysregulated were labelled (those with a 4-fold change: -2 2\> +2) and the full list of DEGs provided in the supplemental excel file. We have added the suggested network analysis, and for additional rigor also included protein interaction partners of Gli1 and Arhgap36 (Fig. S12).

(2) Likewise, the ChIP-seq data in Figure 2 are under-analyzed, focusing only on the ARHGAP36 locus and not more broadly on the FOXC1 gene expression program. This is a missed opportunity that should be remedied with unbiased analyses intersecting differentially expressed FOXC1 peaks with differentially expressed genes from RNA-sequencing data displayed in Figure 1.

We agree that genome-wide analysis of ChIP-seq data from Foxc1 over-expression is worthwhile, not least for diverse malignancies where FOXC1 is over-expressed. We chose to restrict the focus of this paper in order to define, as comprehensively as we could, the FOXC1 - ARHGAP36 relationship. Our ChIP and RNA-seq datasets are freely available to other researchers via GEO (GSE297865/GSE297719). Our future manuscript is integrating ChIP-seq and RNA-seq with ATAC-seq: replicate ATAC-seq experiments permit rigorous characterization of genes transcriptionally regulated by Foxc1 as well as Foxc1’s pioneering abilities. However, these additional assays, and particularly validation of findings, take significant time and so lie beyond the scope of the current manuscript.

(3) RNA-seq and ChIP-seq data strongly suggest that FOXC1 regulates ARHGAP36 expression, and the authors convincingly identify genomic segments at the ARHGAP36 locus where FOXC1 binds, but they do not test if FOXC1 specifically activates this locus through the creation of a luciferase or similar promoter reporter. Such a reagent and associated experiments would not only strengthen the primary argument of this investigation but could serve as a valuable resource for the community of scientists investigating FOXC1, ARHGAP36, the Hedgehog pathway, and related biological processes. CRISPRi targeting of the identified regions of the ARHGAP locus is a useful step in the right direction, but these experiments are not done in a way to demonstrate FOXC1 dependency.

We agree and undertook the suggested luciferase reporter assays. The results demonstrate that transcriptional activity is dependent on Foxc1 and abrogated by mutation of the predicted Foxc1binding motifs (Fig. S8).

(4) It would be useful to see individual fluorescence channels in association with images in Figure 3b.

The figure has been revised to provide individual fluorescence channel data, as suggested.

(5) Perhaps the most significant limitation of this study is the omission of in vivo data, a shortcoming the authors partly mitigate through the incorporation of clinical outcome data from pediatric neuroblastoma patients in the context of ARHGAP36 expression. The authors also mention that high levels of ARHGAP36 expression were also detected in "specific CNS, breast, lung, and neuroendocrine tumors," but do not provide clinical outcome data for these cohorts. Such analyses would be useful to understand the generalizability of their findings across different cancer types. More broadly, how were high, medium, and low levels of ARHGAP36 expression identified? "Terciles" are mentioned, but such an approach is not experimentally rigorous, and RPA or related approaches (nested rank statistics, etc) are recommended to find optimal cutpoints for ARHGAP36 expression in the context of neuroblastoma, "specific CNS, breast, lung, and neuroendocrine" tumor outcomes.

The issue of analyzing in vivo data for neuroblastoma is addressed in more detail below, as it is also raised by the other reviewers. The neuroblastoma data represent the initial findings after the Foxc1Arhgap36 link was defined. There is vastly more that could and should be undertaken to determine mechanism(s) for ARHGAP36’s beneficial association with this tumor’ survival. This is the ongoing focus for the lab.

The original text omitted details of the cancer expression datasets surveyed that revealed high levels of ARHGAP36 expression were also detected in "specific CNS, breast, lung, and neuroendocrine tumors". This oversight has been corrected – when submitting, we omitted to upload a supplemental file (Table S4) that provided these data, which were derived from the following four sites (TCGA, TARGET, PCAWG and CCLE). However, these excellent online resources infrequently provide clinical outcome data.

The three independent neuroblastoma cohorts were analyzed identically. Each was stratified into an ordered dataset for ARHGAP36 expression, and then divided into three equal-sized groups [terciles]. Stratification into smaller subgroups [quartiles/quintiles] would have been equally feasible. The same methodology is used by the UCSC Xena browser for Kaplan-Meier survival analysis, and offers the advantage of avoiding a *priori* assumptions; it is thus agnostic regarding the data. We agree that there is scope for additional approaches, including recursive partitioning analyses, but suggest it may be better to reserve these for the future, not least in analyses that test the reported ARHGAP36-survival association in additional neuroblastoma datasets.

**Reviewer #2 (Public review):**

FOXC1 is a transcription factor essential for the development of neural crest-derived tissues and has been identified as a key biomarker in various cancers. … Together, these findings uncover a novel FOXC1-ARHGAP36 regulatory axis that modulates Hh and PKA signaling, offering new insights into both normal development and cancer progression.

The main strengths of the study are:

(1) Identification of a novel signaling pathway involving FOXC1 and ARHGAP36, which may play a critical role in both normal development and cancer biology.

(2) Mechanistic investigation using RNA-seq, ChIP-seq, and functional assays to elucidate how FOXC1 regulates ARHGAP36 and how this axis modulates Hh signaling.

(3) Clinical relevance demonstrated through analysis of neuroblastoma patient datasets, linking ARHGAP36 expression to improved 5-year overall survival.

The main weaknesses of the study are:

(1) Lack of validation in neuroblastoma models - the study does not directly test its findings in neuroblastoma cell models, limiting translational relevance.

We agree that the mechanisms by which increased ARHGAP36 levels are protective, are important to define. Despite experiments over many months manipulating ARHGAP36 expression, that induce quite rapid death of neuroblastoma cells in vitro, the precise mechanism(s) remain unresolved. Currently, we are endogenously labelling multiple neuroblastoma lines with Histone 2B-mCherry to facilitate live cell imaging and differentiate effects on proliferation and apoptosis. In the interim, we believe publication of the current dataset allows other researchers to independently test our findings for this pediatric malignancy. We are also establishing collaborations to access patient tissue samples, that will facilitate investigation of non cell autonomous mechanisms mediated via the tumor microenvironment.

(2) Incomplete mechanistic insight into PKA regulation - the study does not fully elucidate how FOXC1-ARHGAP36 regulates PKAC activity at the molecular level.

Other laboratories elegantly demonstrated that ARHGAP36’s effect on Hedgehog output is mediated by one motif blocking PKAC activity and the targeting of PKAC for degradation [PMIDs 25024229, 27713425, 30598432]. With these effects well-established, we limited experiments to confirming that Foxc1induced Arhgap36 reduced PKAC, and pT197 PKAC levels, to those of ectopic Arhgap36 expression.

(3) Insufficient discussion of clinical outcome data - while ARHGAP36 expression correlates with improved survival in neuroblastoma, the manuscript lacks a clear interpretation of this unexpected finding, especially given the known oncogenic roles of FOXC1, ARHGAP36, and Hh signaling.

ARHGAP36 expression may influence neuroblastoma survival via multiple mechanisms. Considering just canonical Hedgehog, possibilities include: cell cycle modulation, symmetric vs asymmetric cell division, maintenance of cancer stem cells, EMT, metastasis… Others include Hedgehog’s anti-apoptotic roles and the diverse mechanisms by which PKA influences cell function and survival. Faced with such diversity, we focused the discussion on what the presented data demonstrate.

**Reviewer #3 (Public review):**

Summary:

The focus of the research is to understand how transcription factors with high expression in neural crest cell-derived cancers (e.g., neuroblastoma) and roles in neural crest cell development function to promote malignancy. The focus is on the transcription factor FOXC1 and using murine cell culture, gain- and loss-of-function approaches, and ChIP profiling, among other techniques, to place PKC inhibitor ARHGAP36 mechanistically between FOXC1 and another pathway associated with malignancy, Sonic Hedgehog (SHH).

Strengths:

Major strengths are the mechanistic approaches to identify FOXC1 direct targets, definitively showing that FOXC1 transcriptional regulation of ARHGAP36 leads to dysregulation of SHH signaling downstream of ARHGAP36 inhibition of PKC. Starting from a screen of Foxc1 OE to get to ARHGAP36 and then using genetic and pharmacological manipulation to work through the mechanism is very well done. There is data that will be of use to others studying FOXC1 in mesenchymal cell types, in particular, the FOXC1 ChIP-seq.

Weaknesses:

Work is almost all performed in NIH3T3 or similar cells (mouse cells, not patient or mouse-derived cancer cells), so the link to neuroblastoma that forms the major motivation of the work is not clear. The authors look at ARHGAP36 levels in association with the neuroblastoma patient survival; however, the finding, though interesting and quite compelling, is misaligned with what the literature shows about FOXC1 and SHH, their high expression is associated with increased malignancy (also maybe worse outcomes?). Therefore, ARHGAP36 expression may be more complicated in a tumor cell or may be unrelated to FOXC1 or SHH, leaving one to wonder what the work in NIH3T3 cells, though well done, is telling us about the mechanisms of FOXC1 as an oncogene in neuroblastoma cells or in any type of cancer cell. Does it really function as an SHH activator to drive tumor growth? The 'oncogenic relevance' and 'contribution to malignancy' claimed in the last paragraph of the introduction are currently weakly supported by the data as presented. This could be improved by studying some of these mechanisms in patient-derived neuroblastoma cells with high FOXC1 expression. Does inhibiting FOXC1 change SHH and ARHGAP36 and have any effect on cell proliferation or migration? Alternatively, does OE of FOXC1 in NIH3T3 cells increase their migration or stimulate proliferation in some way, and is this dependent on ARHGAP36 or SHH? Application of their mechanistic approaches in cancer cells or looking for hallmarks of cancer phenotypes with FOXC1 OE (and dependent on SHH or ARHGAP36) could help to make a link with cellular phenotypes of malignant cells.

The manuscript stems from the lab’s findings that Foxc1 influences cilia-mediated signaling (Hedgehog and PDGFRalpha), offering an explanation for FOXC1’s pleiotropic phenotypes. Due to FOXC1’s largely unexplained roles in malignancy, the effects on Hedgehog prompted investigation of differential gene expression in NIH3T3 cells when Foxc1 was over-expressed. This identified Arhgap36 as a prime candidate for the Hedgehog pathway alterations, and most of the paper reports the characterization of this relationship. The final, small component of the paper, tests the relevance in neural crest derived cells, where Foxc1 has key roles. Neuroblastoma’s frequent lethality has created a network of highly supportive researchers with shared datasets, and these survival data were assayed. This in turn revealed that high levels of ARHGAP36 expression were associated with a favorable survival outcome.

Defining the underlying molecular mechanisms for this novel association is clearly important. As outlined above, one challenge reflects the diversity of potential mechanisms, coupled with the requirement to validate those identified from 2-D culture in patient-derived tumor explants as well as immuno-deficient model organisms. Such experiments take significant time, and our present focus is on manipulating ARHGAP36 expression directly, rather than by altering FOXC1 expression, which inevitably has even more diverse effects.

**Recommendations for the authors:**

**Reviewer #2 (Recommendations for the authors):**

The study would be strengthened by validating key findings, such as the resistance to Hh inhibition, in neuroblastoma cell lines to enhance disease relevance.

Planned future experiments include in vitro evaluation of PKA antagonists and agonists on neuroblastoma survival.

The authors show that FOXC1/ARHGAP36 reduces PKAC protein levels; however, it is unclear whether this regulation occurs at the transcriptional level. Assessing PKAC mRNA expression would help explain the mechanism. Additionally, if PKAC is transcriptionally downregulated, overexpression of PKAC can be used to test whether it reverses the FOXC1/ARHGAP36induced activation of Hh signaling.

The RNA-sequencing data exclude this possibility at the transcriptional level, since PKA is not significantly differentially expressed (Table S1). Instead, Figures 1&3 support Foxc1 inducing Arhgap36 expression, with elevated Arhgap36 protein levels reducing those of PKAC and catalytically active pT197 PKAC, in both the cytoplasm and adjacent to the basal body.

The Discussion should address the potential effects of ARHGAP36 overexpression on other signaling pathways-particularly Hh and PKA signaling and PKA in neuroblastoma. These effects may help interpret the observed association between ARHGAP36 expression and clinical outcomes in patients. Of note, it has been reported that Hh may correlate with better survival in neuroblastoma (Cancers, 2021 Apr 15;13(8):1908; J Pediatr Surg. 2010 Dec;45(12):2299).

Both Hedgehog signaling and protein kinase A have broad effects on normal cell biology, that are likely more extensive in malignant cells. Consequently, although tempting to propose why ARHAGP36 overexpression is associated with enhanced survival, it may be better to wait until the causative mechanisms have been defined.

If treatment information for the patient cohorts is available, it should be included as it may enhance the interpretability of the survival analyses.

This is an excellent suggestion, although at present this information is not available to us. As the manuscript moves forward to publication, we will be liaising with the corresponding authors of the three datasets [GSE49711, E-MTAB-178191 and TARGET] to explore such additional clinical possibilities.

The 'A' label in Figures S9 and S10 should be removed, as neither figure contains sub-panels.

This has been corrected, as suggested.

**Reviewer #3 (Recommendations for the authors):**

Other comments:

(1) Figure 5A, B: Unclear how meaningful the inhibitor experiments are in the absence of SHH (presumable none in the media or made by NIH3T3 cells?), other than as a control for the FOXC1 OE treated with Smo antagonists. A potentially better experiment could be to take malignant cells with high FOXC1 and high SHH signaling and put on Smo inhibitors.

Figure 5A demonstrates Foxc1’s induction of GLI1 expression is not dependent on Hedgehog ligand. While certainly feasible to repeat in malignant cells strongly expressing FOXC1, doing this comprehensively would require testing lines from many or all of the ~15 malignancies where FOXC1 has a defined contribution.

(2) Figure 6: the Gli2-mGFP seem to have higher levels of ciliary Sufu, they also have higher levels of Gli1 (see Figure 1C), does the Gli2-mGFP expression change SHH signaling? What controls have the authors done to test if this is a serious confound in their studies? They use it for most experiments, this is important to address.

Although Gli2-mGFP expression affects Hedgehog signaling, in the absence of Gli2 (e.g. untransformed NIH3T3) Foxc1 induces Arhgap36 expression. The scope for interaction between Foxc1 and Gli2 represents an additional motivation for the ATAC-seq experiments described above to better determine if these two transcription factors have synergistic effects.

(3) Figure 3B: (1) Please use color-blind friendly LUTs for the signals (same comment for other figures), (2) The Gli2-mGFP line with the current color scheme is confusing; it looks like only 647 and 555 secondaries were used, did they not image with the mGFP? Why not? (3) What is the evidence that these are basal bodies? (4) Why did the authors use cycloheximide in these IF experiments? Was this also done in other methods? The reasoning behind this is missing.

For now, we have included separate channels for Figure 3. In future manuscripts we will adopt the suggestion of moving to either magenta and green, or cyan and magenta combinations for depicting immunofluorescence.
